# Urolithin A activates aryl hydrocarbon receptor-NLRP6-mediated pathways in intestinal epithelial cells to modulate mucosal immunity and strengthen gut barrier integrity

**DOI:** 10.1038/s41467-026-73760-3

**Published:** 2026-06-23

**Authors:** Sweta Ghosh, Zachary M. Vanwinkle, Sobha Rani Bodduluri, Subir Kumar Juin, Mahendar Kadari, Ankita Singh, Gerald W. Dryden, Matthew B. Lawrenz, Thirumala-Devi Kanneganti, Shesh N. Rai, Misty Good, Pawan Kumar, Bodduluri Haribabu, Venkatakrishna Rao Jala

**Affiliations:** 1 Department of Microbiology and Immunology, UofL-Brown Cancer Center, Center for Microbiomics, Inflammation and Pathogenicity, University of Louisville, Louisville, KY USA; 2 Department of Microbiology and Immunology, Center for Predictive Medicine for Biodefense and Emerging Infectious Diseases, University of Louisville, Louisville, KY USA; 3 Department of Microbiology and Immunology, Stony Brook University, Stony Brook, NY USA; 4 Department of Medicine, University of Louisville, Louisville, KY USA; 5 Department of Immunology, St. Jude Children’s Research Hospital, Memphis, TN USA; 6 Biostatistics and Informatics Shared Resource, University of Cincinnati Cancer Center, Cancer Data Science Center, University of Cincinnati College of Medicine, Department of Biostatistics, Health Informatics and Data Sciences, University of Cincinnati College of Medicine, Cincinnati, OH USA; 7 Division of Neonatal-Perinatal Medicine, Department of Pediatrics, University of North Carolina at Chapel Hill, Chapel Hill, NC USA

**Keywords:** Ulcerative colitis, Gastrointestinal models

## Abstract

The aryl hydrocarbon receptor (AHR) plays a central role in orchestrating gut barrier and mucosal immune functions in the pathogenesis of inflammatory bowel disease (IBD). Nevertheless, activation of the AHR by diverse ligands yields varied outcomes, and the downstream pathways responsible for these effects remain unknown. Here, we report that selective activation of AHR in mouse intestinal epithelial cells (IEC) by the microbial metabolite, urolithin A (UroA), triggers the Nod-like receptor pyrin domain-containing protein 6 (NLRP6) inflammasome, resulting in the release of interleukin (IL)−18 but not IL-1β. Further, we show that UroA-induced IL-18 in IECs is critical for IL-22, mucin 2 and REG3γ production, as well as protection against colitis. Moreover, UroA significantly upregulates IL-18 and IL-22 levels in IECs and type-3 innate lymphoid cells, respectively, in intestinal biopsies from patients with IBD patients. These results demonstrate that activation of AHR by UroA modulates intestinal barrier function through an NLRP6-IL-18-IL-22 pathway in both healthy and IBD conditions.

## Abstract (web summary)

The aryl hydrocarbon receptor (AHR) is a transcription factor activated by various endogenous and exogenous ligands. Here, the authors demonstrate that selective AHR activation in epithelial cells by the microbial metabolite, urolithin A, induces NLRP6-dependent secretion of IL-18, leading to IL-22 production from ILC3 cells to restore gut homeostasis and mitigate colitis in mice.

## Introduction

The intestinal epithelial barrier is a crucial component of the gastrointestinal (GI) tract responsible for separating bacteria and other microorganisms from the internal environment of the body. Compromised gut epithelial barrier and mucosal immune system lead to various GI disorders, including inflammatory bowel diseases (IBD)^1–3^. The aryl hydrocarbon receptor (AHR) is a ligand-activated transcription factor, which is activated by numerous endogenous and exogenous ligands to play a critical role in the gut homeostasis of both healthy and IBD conditions^4–6^. Expression of AHR and beneficial AHR ligands (tryptophan, indoles) is significantly decreased in the intestines of IBD patients compared to healthy subjects^7,8^. In recent years, there has been a growing recognition of the interplay between AHR, gut microbes, and gut microbial metabolites that influence intestinal health. These intricate interactions have emerged as mechanisms conferring the protective benefits against inflammatory conditions, notably IBD^9,10^. IBD patients with low AHR ligands/AHR activities may be at a disadvantage to maintain healthy gut barrier and more susceptible to severe disease. The cellular and molecular regulatory pathways responsible for gut homeostasis and barrier functions are not fully understood.

Inflammation and cell death induced by inflammasome pathways in the GI tract contribute to the maintenance of gut homeostasis and pathophysiology of IBD conditions^11^. Inflammasomes are cytoplasmic multiprotein complexes that comprise of nucleotide-binding leucine-rich repeat receptors (NLR) include NLRP1, NLRP3, NLCR4 and NLRP6^12,13^. Among NLRs, NLRP6 is predominantly expressed in intestinal epithelial cells and exhibits both inflammasome-dependent and independent roles in the maintenance of intestinal homeostasis^14–18^. Previously, it was shown that *Ahr*^*−/−*^ or *Nlrp6*^*−/−*^ mice exhibit increased susceptibility to dextran sodium sulfate (DSS)-induced colitis suggesting protective roles of AHR and NLRP6 in colitis^10,19–26^. Also, gut microbial metabolites induce production of NLRP6-caspase-1 dependent interleukin (IL)-18 and regulate tissue repair and tolerance to commensal microorganisms to maintain gut homeostasis^17^. In contrast, it was also reported that excess production of IL-18 can be detrimental in colitis by targeting goblet cells leading to reduced levels of mucin^27^. Excessive IL-18 production proves detrimental in conditions related to IBD, while the complete absence of IL-18 results in significant damage to the gut environment, making individuals more prone to IBD. The precise regulators and mechanisms responsible for dual roles of IL-18 in gut homeostasis, and the role of xenobiotic-inflammasome interactive pathways remain unidentified.

A natural gut microbial metabolite, Urolithin A (UroA), produced from ellagic acid or ellagitannin-containing foods such as pomegranates, was shown to enhance gut barrier function and protect against chemical-induced colitis in pre-clinical models^22,28,29^. We and others have demonstrated that UroA acts as a low-affinity AHR ligand that activates AHR signaling pathways without displaying toxicity^22,28,30,31^. In the current study, we discovered that selective activation of intestinal epithelial cell (IEC)-AHR by UroA triggers NLRP6 activation to induce homeostatic IL-18 from IECs. UroA-induced IEC-IL-18, which in turn stimulates tissue protective IL-22 production from innate lymphoid type 3 (ILC3) cells, leading to increased expression of mucin 2 (MUC2) and Regenerating Family Member 3 Gamma (REG3γ) to strengthen gut barrier function and protect against colitis. Importantly, our data unveiled that UroA treatment induces IL-18 and IL-22 in intestinal biopsies of IBD patients, indicating potential therapeutic options to reduce or attenuate IBD pathogenesis.

## Results

### IEC-AHR is required for UroA-mediated protective activities

Previously, we demonstrated that UroA enhances gut barrier function and reduces intestinal inflammation, mitigating colitis in pre-clinical models in an AHR-CYP1-dependent manner^22,28^. We showed that UroA activated AHR-dependent signaling pathways both in macrophages and gut epithelial cells^22^. To determine the cell-specific requirement of AHR for UroA-mediated protective activities, we generated *Ahr*^fx^-*Villin*^Cre^ (*Ahr* is deleted in intestinal epithelial cells)^32^ and *Ahr*^fx^-*LysM*^Cre^ (*Ahr* is deleted in myeloid cells) mice by crossing *Ahr*^fx/fx^ with *Villin*^Cre^ and *LysM*^Cre^, respectively. We used *Ahr*^fx/fx^ and *Ahr*^−/−^ mice as controls. The cell-specific deletion of AHR in these mice was confirmed by genotyping PCR, immunofluorescence and western blot analysis (Fig. S1A, B). UroA-AHR-dependent anti-inflammatory activities were investigated using bone marrow-derived macrophages (BMDM) prepared from *Ahr*^fx/fx^, *Ahr*^−/−^, *Ahr*^fx^-*Villin*^Cre^ and *Ahr*^fx^-*LysM*^Cre^ mice. As shown in Fig. S1C, UroA failed to reduce LPS-induced IL-6 and TNF-α in BMDM prepared from *Ahr*^−/−^ and *Ahr*^fx^-*LysM*^Cre^ mice. However, UroA treatment significantly reduced LPS-induced IL-6 and TNF-α in BMDM isolated from *Ahr*^fx/fx^ and *Ahr*^fx^-*Villin*^Cre^ mice. These results suggest that AHR is functionally active in myeloid cells in *Ahr*^fx^-*Villin*^Cre^ mice, but not in BMDM of *Ahr*^fx^-*LysM*^Cre^ mice.

**Fig. 1:**
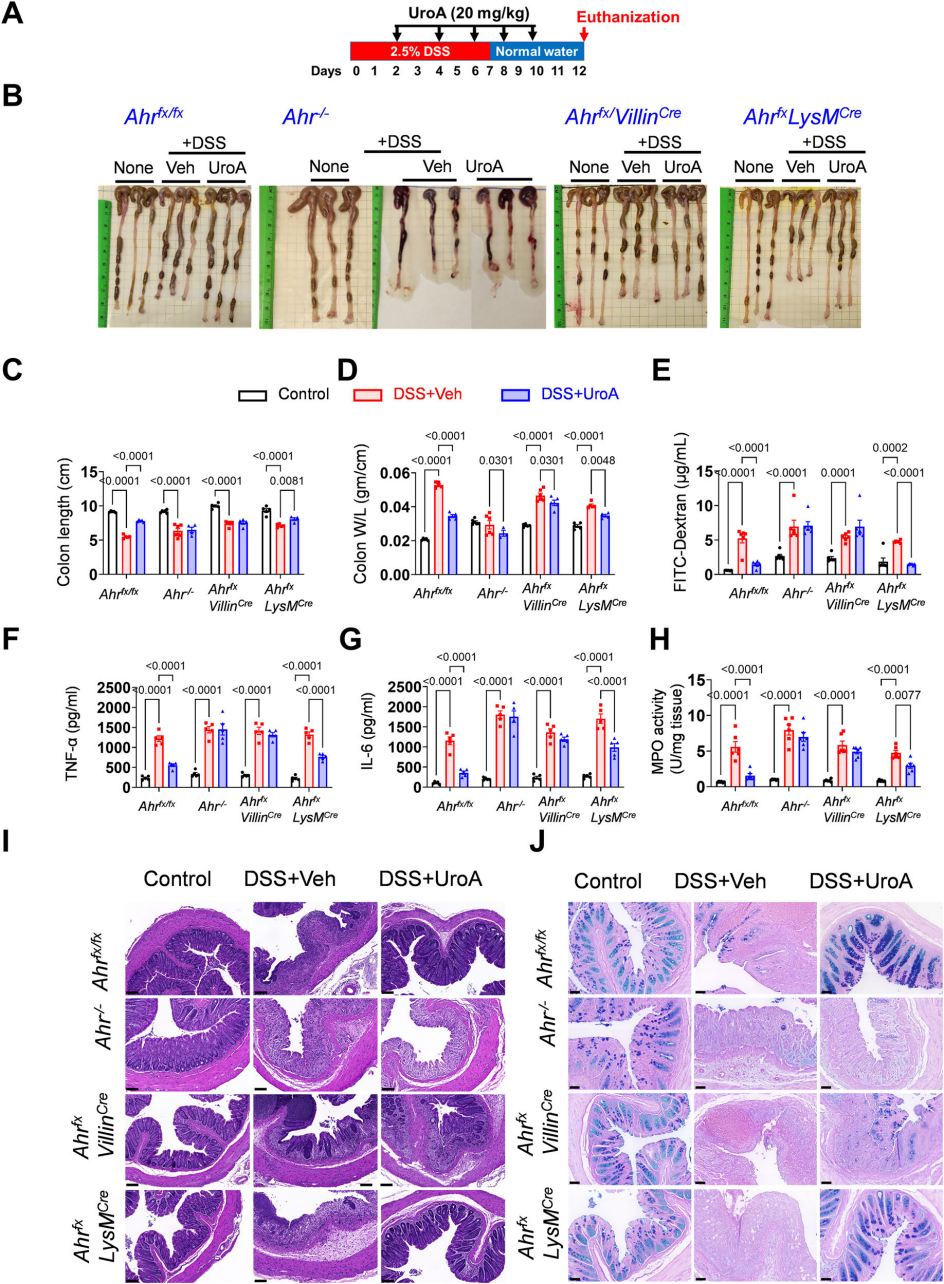
Intestinal AHR is required for UroA-mediated protection against colitis. **A** Schematic diagram of the DSS-induced colitis model shows the frequency of UroA treatment. *Ahr*^fx/fx^, *Ahr*^−/−^, *Ahr*^fx^-*Villin*^Cre^ and *Ahr*^fx^-*LysM*^Cre^ mice (6–8 week old age, *n* = 5–8, consisting of male (*n* = 3, 4) and female (*n* = 2–4) mice per group) were kept on 2.5% DSS drinking water for 7 days, followed by 5 days of regular water. Mice were treated with either Vehicle (1% CMC, 0.1% Tween-80) or UroA (20 mg/kg) orally every 48 h post DSS treatment. Control mice received normal water. All mice were euthanized on day 12. Representative data from one of three independent experiments is shown. **B** Gross images of colons of indicated genotypes are represented. **C** Colon lengths were measured. **D** Ratio of Colon weight/length is shown. **E** Intestinal permeability was assessed by using the FITC-dextran intestinal permeability assay as described in the methods. **F**,** G** Serum TNF-α and IL-6 levels were determined using standard ELISA methods. **H** Colonic MPO levels were evaluated by ELISA using colon tissue homogenates. **I** Microphotographs of hematoxylin and eosin (H&E) stained sections of the colon are shown. Scale bar indicates 100 μm. **J** Micrographs of Alcian Blue-Periodic Acid Schiff (AB-PAS) stained sections are shown. Scale bar indicates 50 μm. Statistics were performed using two-way ANOVA. Error bars, ±SEM; *p*-values that are <0.05 are indicated. All experiments were repeated at least three times using biologically independent replicates, yielding similar results. Source data are provided as a Source data file.

To define the cell-specific requirement of AHR expression for UroA-mediated activities in vivo, *Ahr*^fx/fx^, *Ahr*^−/−^ *Ahr*^fx^-*Villin*^Cre^ and *Ahr*^fx^-*LysM*^Cre^ mice were subjected to acute DSS-induced colitis, where mice were allowed to drink water containing 2.5% DSS for 7 days and followed by 5 days of regular water (Fig. 1A). Mice were euthanized on day 12 post DSS treatment. The colitis phenotypes were characterized by evaluating colon lengths, colon length/weight, intestinal permeability, histology, serum cytokines and colonic myeloperoxidase (MPO) levels as described by Singh et al.^22^. As shown in Fig. S2, treatment with UroA significantly protected from DSS-induced body weight loss in *Ahr*^fx/fx^ and *Ahr*^fx^-*LysM*^Cre^ mice but failed in *Ahr*^−/−^ and *Ahr*^fx^-*Villin*^Cre^ mice. Similarly, UroA treatment also protected from inflammation-induced shortening of colons (Fig. 1B–D). In corroboration with these observations, UroA treatment also reduced intestinal permeability in *Ahr*^fx/fx^ and *Ahr*^fx^-*LysM*^Cre^ mice, but not in *Ahr*^−/−^ and *Ahr*^fx^-*Villin*^Cre^ mice (Fig. 1E). Further, UroA treatment reduced DSS-induced serum inflammatory cytokines (IL-6 and TNF-α) and MPO in colons of *Ahr*^fx/fx^ and *Ahr*^fx^-*LysM*^Cre^ mice but not in *Ahr*^−/−^ mice and *Ahr*^fx^-*Villin*^Cre^ (Fig. 1F–H). Analysis of colon histology by hematoxylin and eosin (H & E) staining (Fig. 1I) revealed that DSS-induced immune cell infiltration and loss of epithelial membrane integrity were not restored in *Ahr*^−/−^ and *Ahr*^fx^-*Villin*^Cre^ mice upon UroA treatment. Histological scores suggested that UroA failed to restore the DSS-induced colon epithelial damage in *Ahr*^−/−^ and *Ahr*^fx^-*Villin*^Cre^ mice compared to littermate *Ahr*^fx/fx^ mice (Fig. S3A). However, UroA treatment protected *Ahr*^fx/fx^ and *Ahr*^fx^-*LysM*^Cre^ mice from DSS-induced colonic damage. Analysis of mucin levels by Alcian Blue-Periodic Acid Schiff (AB-PAS) staining suggested that UroA treatment restored the mucin levels in *Ahr*^fx/fx^ and *Ahr*^fx^-*LysM*^Cre^ mice but not in *Ahr*^−/−^ and *Ahr*^fx^-*Villin*^Cre^ mice (Figs. 1J, S3B). Next, we confirmed the UroA-mediated AHR-dependent Cytochrome P450 Family 1 Subfamily A Member 1 (CYP1A1) induction in the colon by measuring the expression of *Cyp1a1*. As shown in Fig. S3C, UroA treatment induced *Cyp1a1* in colons of *Ahr*^fx/fx^ mice, but not in colons of *Ahr*^fx^-*Villin*^Cre^ mice. As expected, UroA treatment significantly increased expression of *Cyp1a1* in *Ahr*^fx/fx^ mice treated with DSS + UroA, compared with DSS + Vehicle (Fig. S3C). Interestingly, we observed low levels of *Cyp1a1* expression in the colons of *Ahr*^fx^-*Villin*^Cre^ mice when exposed to DSS and treated with UroA. It is possible that UroA potentially induced *Cyp1a1* in infiltrated myeloid cells, leading to observable changes (Fig. S3C). Overall, these results suggested that UroA-mediated anti-colitogenic activities are dependent upon AHR expression in IEC, but not in myeloid cells.

### Selective activation of IEC-AHR is critical for IL-22 production from ILC3

**Fig. 2:**
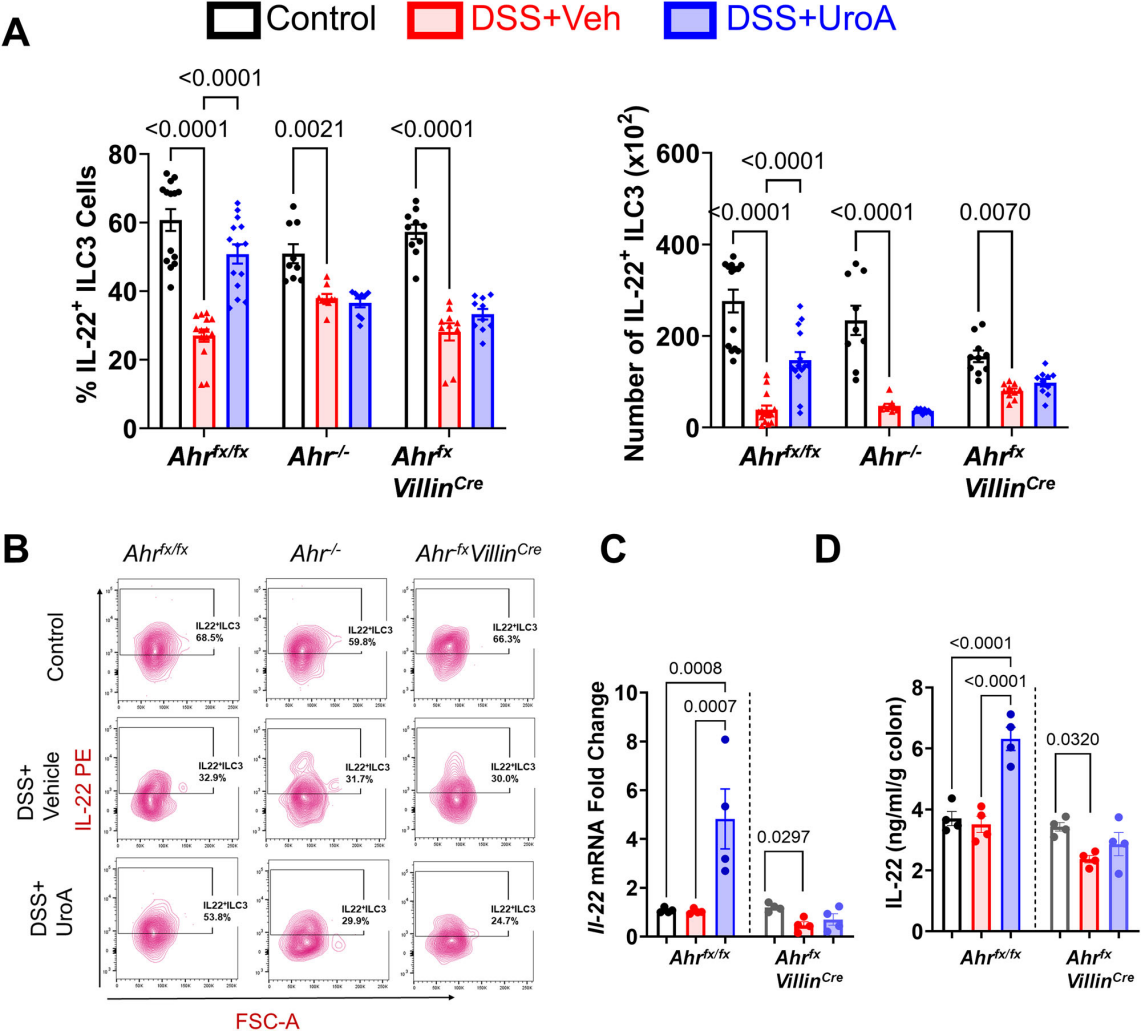
Intestinal AHR is required for UroA-mediated IL-22 production from Type 3 innate lymphoid cells (ILC3). *Ahr*^fx/fx^ and *Ahr*^fx^-*Villin*^Cre^ mice (6–8 week old age, *n* = 5–7, consisting of male (*n* = 3–4) and female (*n* = 2–3) mice per group) were 2.5% DSS containing water for 7 days followed by 5 days of regular water and were treated with either Vehicle or UroA (20 mg/kg) orally every 48 h starting from day 0. **A** Single-cell suspensions from lamina propria (LP) were prepared from the colon of *Ahr*^fx/fx^, *Ahr*^−/−^ and *Ahr*^fx^-*Villin*^Cre^ mice. The IL-22^+^ ILC3s were measured using standard flow cytometry methods as described in the methods, and the gating strategy is shown in Fig. S3. Percentage and total number of IL22^+^ILC3 cells are shown. Statistics were performed two-Way ANOVA test. Error bars, ±SEM; ns: Not significant, **** *p* < 0.0001. **B** Flow cytometry diagrams of IL-22 levels are shown for the indicated mice and treatments. **C** The fold changes of *Il-22* mRNA levels in the colons of *Ahr*^fx/fx^ and *Ahr*^fx^-*Villin*^Cre^ mice were determined by the SYBR green RT-PCR method. **D** Protein levels of IL-22 were determined in colon homogenates using standard ELISA. Statistics were performed using one-way ANOVA. Error bars, ±SEM; *p*-values < 0.05 are shown. All experiments were repeated at least three times using biologically independent replicates, yielding similar results. Source data are provided as a Source data file.

IL-22, a known tissue protective cytokine, strengthens the epithelial barrier by inducing mucins and anti-microbial mediators as well as promoting wound healing mechanisms^33,34^. Previous studies suggested that AHR activation in ILC3 leads to the induction of IL-22^35,36^. We asked whether UroA treatment leads to the generation of IL-22 in an AHR-dependent manner. The IL-22 levels from the lamina propria ILC3 were measured in *Ahr*^fx/fx^*, Ahr*^−/−^ and *Ahr*^fx^-*Villin*^Cre^ mice. As shown in Fig. 2A, IL-22 from ILC3 is significantly reduced in DSS-induced colitis mice compared with control mice on day 12 post DSS. UroA treatment restored the IL-22 levels in *Ahr*^fx/fx^ mice that were subjected to DSS-induced colitis, but not in *Ahr*^*−/−*^ mice. Interestingly, UroA also failed to induce IL-22 from ILC3 from *Ahr*^fx^*-Villin*^Cre^ mice (Fig. 2A, B), indicating that UroA requires IEC-AHR to induce IL-22 from ILC3. The flow cytometry gating strategy for ILC3^+^IL-22^+^ cells is provided in Figs. 2B, S4. The flow cytometry experiments were performed using appropriate controls, including the use of *Il-22*^*−/−*^ mice, IL-23 + IL-1β induction to upregulate IL-22, as well as fluorescence minus one staining (FMO) (Fig. S5). Further, expression of IL-22 at mRNA and protein levels in colon tissues was significantly increased upon UroA treatment in *Ahr*^fx/fx^ mice but not in *Ahr*^fx^-*Villin*^Cre^ mice, indicating IEC-AHR requirement for IL-22 production (Fig. 2C, D). To define the IEC-AHR role in UroA-mediated IL-22 production in non-inflammatory conditions, we treated naïve wild-type, *Ahr*^*−/−*^, *Ahr*^fx/fx^ and *Ahr*^fx^-*Villin*^Cre^ with vehicle and UroA, and colonic IL-22 levels were determined. As shown in Fig. S6, UroA treatment led to increased levels of IL-22 in wild-type and *Ahr*^fx/fx^ mice, but not in *Ahr*^*−/−*^, and *Ahr*^fx^-*Villin*^Cre^ mice, suggesting IEC-AHR is required for UroA-mediated induction of IL-22. Next, we determined IL-22 levels in ILC3, ILC3 NCR^+^, ILC3 NCR^-^, CD4^-^Lti and CD4^+^Lti populations in WT mice that are subjected to DSS and DSS+UroA treatments to define the source of ILC3 (Fig. S7). These data suggested that UroA upregulated IL-22 in ILC3, ILC3 NCR^+^, ILC3 NCR^-^ and CD4^-^Lti populations, but not CD4^+^Lti population.

We examined UroA-mediated IL-22 production in the *Citrobacter rodentium*-induced colitis model in wild-type (WT) mice (C57BL/6 J) (Fig. S8). *C. rodentium* infection is a well-established model to investigate therapeutics and IL-22 role in colitis^37,38^. For this model, mice were infected with *C. rodentium* and then received alternate-day treatment with either vehicle or UroA (Fig. S8A). Euthanasia was performed on the 10^th^ day post-infection. Our results indicated that UroA treatment alleviated *C. rodentium*-induced colitis in WT mice. As shown in Fig. S8B–F, UroA treatment protected these mice from rapid loss of body weight, shortening of the colon, dysregulation of colon weight/length and barrier dysfunction as measured by FITC-dextran. Analysis of IL-22^+^-ILC3 cells from lamina propria (LP) revealed that UroA treatment increased the IL-22^+^-ILC3 cells in *C. rodentium*-infected mice (Fig. S8G). Consistent with these findings, H&E analysis of colon tissue sections also showed lesser inflammation and tissue damage upon UroA treatment compared with vehicle alone (Fig. S8H, I). Overall, these studies suggested that UroA treatment induced IL-22 during colitis in an IEC-AHR-dependent manner.

### UroA-activated IEC-AHR regulates IL-22 production through IEC-IL-18 signaling

**Fig. 3:**
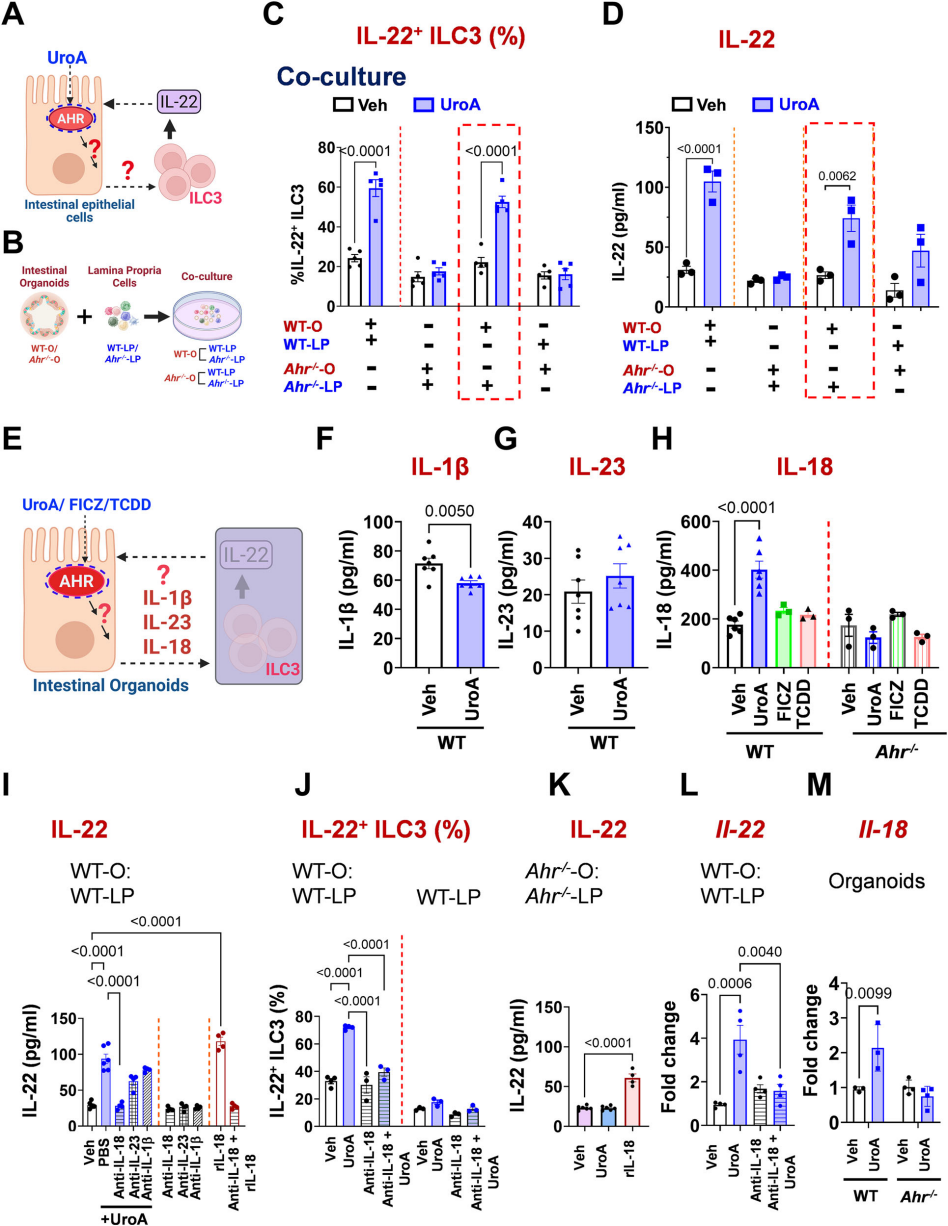
UroA-mediated IL-22 induction is dependent upon expression of intestinal epithelial cell (IEC)-AHR. **A** Schematic representation of testing the hypothesis that UroA requires IEC-AHR to induce IL-22. Created in BioRender. Ghosh, S. (2026) https://BioRender.com/bpbcn69. **B** Experimental models of co-cultures of intestinal organoids and lamina propria lymphocytes. WT-O: Small intestinal organoids prepared from wild-type mice; *Ahr*^*−/−*^-O: small intestinal organoids prepared from *Ahr*^*−/−*^ mice. WT-LP: Lamina propria cells prepared from wild-type mice; *Ahr*^*−/−*^-LP: lamina propria cells prepared from *Ahr*^*−/−*^ mice. Created in BioRender. Ghosh, S. (2026) https://BioRender.com/c7c5z9c. **C** Intestinal organoids (*n* = 100 organoids from WT or *Ahr*^*−/−*^ mice per sample, (*n* = 9)) were cultured with or without LP cells (1 × 10^5^ cells from WT and *Ahr*^*−/−*^ mice) for 3 days in the presence of Veh (0.05% DMSO) or UroA (25 µM). The IL-22-producing ILC3 were analyzed using flow cytometry as described in Fig. 2A. Dotted red box highlights WT-O:*Ahr*^*−/−*^-LP co-cultures, indicating AHR on IEC is required for IL-22 production. **D** IL-22 levels were determined in supernatants of indicated co-cultures using standard ELISA in a separate set of experiments. **E** Schematic diagram to test the hypothesis that AHR activation leads to the generation of IL-1β, IL-23 and IL-18 to induce IL-22 from ILC3. Created in BioRender. Ghosh, S. (2026) https://BioRender.com/jgcw48w. **F**,** G** WT intestinal organoids (*n* = 100 organoids per sample, (*n* = 7)) were treated with Vehicle (0.05% DMSO) or UroA (25 µM) and measured IL-1β (**F**), IL-23 (**G**) in the supernatants using ELISA methods. **H** WT and *Ahr*^*−/−*^ intestinal organoids were treated with Vehicle (0.05% DMSO) or UroA (25 µM) or FICZ (50 nM) or TCDD (10 nM) for 72 h. The levels of IL-18 were measured in the supernatants using ELISA methods. **I** Co-cultures of intestinal organoids (*n* = 100 organoids per sample, (*n* = 4–6)) and LP cells from WT mice were treated with Vehicle (Veh, 0.05% DMSO) or UroA (25 μM) or recombinant IL-18 (10 ng/ml) for 72 h. These cultures were co-treated with neutralizing anti-IL-18 antibody or anti-IL-23 antibody, anti-IL-1β antibody (20 ng/ml) in the presence or absence of UroA. IL-22 levels were measured by ELISA. **J** Co-cultures of intestinal organoids (*n* = 100 organoids per sample, (*n* = 3–4)) and LP cells or only LP cells from WT mice were treated with Vehicle (Veh, 0.05% DMSO) or UroA (25 μM) or recombinant IL-18 (10 ng/ml) for 72 h. These cultures were co-treated with neutralizing anti-IL-18 antibody. The percentage of IL-22^+^ ILC3 cells was determined by flow cytometry as described in the methods. **K** *Ahr*^−/−^ intestinal organoids (*n* = 100 organoids per sample, (*n* = 4–6)) and *Ahr*^−/−^ LP cells (1 × 10^5^ cells) were treated with Vehicle (0.05% DMSO) or UroA (25 µM) or recombinant IL-18 (10 ng/ml) for 72 h. IL-22 levels were measured in supernatants by ELISA. **L** The fold changes in mRNA levels of *Il-22* in co-cultures of intestinal organoids (*n* = 100 organoids per sample, (*n* = 4)) and LP cells (1 × 10^5^ cells) from WT mice were determined by the SYBR green RT-PCR method. **M** WT and *Ahr*^−/−^ intestinal organoids (*n* = 100 organoids per sample, (*n* = 3–4)) were treated with Vehicle (0.05% DMSO) or UroA (25 µM) for 72 h. The fold changes in mRNA levels of *Il*-18 were determined by the SYBR green RT PCR method. Statistics were performed using two-way ANOVA. Error bars, ±SEM; *p*-values < 0.05 are shown. All experiments were repeated at least three times using biologically independent replicates, yielding similar results. Source data are provided as a Source data file.

To define UroA-mediated IEC-AHR-dependent signaling pathways in IL-22 production by UroA (Fig. 3A), we adopted a co-culture system of ‘intestinal organoids (O) and lamina propria (LP) lymphocytes’ (Fig. 3B). Intestinal organoids were prepared from C57BL/6 J wild type (WT) and *Ahr*^−/−^ mice using standard protocols described by StemCell Technologies. LP cells were isolated and co-cultured with organoids^39,40^. The representative bright field images of small intestinal and colon organoids are shown in Figs. S9A, S9B. In this experimental setup, WT-organoids (WT-O) or *Ahr*^*−/−*^-organoids (*Ahr*^*−/−*^-O) were co-cultured either with WT-LP or *Ahr*^*−/−*^-LP for 3 days (Fig. 3B). The co-cultures were treated with vehicle (DMSO, 0.05%) or UroA (25 μM) for 72 h. The IL-22^+^ ILC3 were gated using standard flow cytometric methods. As shown in Fig. 3C, UroA induced IL-22 in WT-O:WT-LP co-cultures, whereas UroA failed to induce IL-22 in *Ahr*^−/−^-O:*Ahr*^*−/−*^-LP co-cultures, suggesting the requirement of AHR for UroA-mediated IL-22 production. To define the role of IEC-AHR, we performed cross-co-culture experiments, where WT-O:*Ahr*^*−/−*^-LP and *Ahr*^*−/−*^-O:WT-LP were treated with UroA, and IL-22 levels were determined. As shown in Fig. 3C, D, UroA failed to induce IL-22 in *Ahr*^*−/−*^-O:WT-LP but generated a significant increase in IL-22 in WT-O:*Ahr*^*−/−*^-LP co-cultures. These results indicated that UroA required IEC-AHR for IL-22 production and was independent of ILC3-AHR. It is possible that UroA may induce a mediator in an IEC-AHR–dependent manner that subsequently drives IL-22 production by ILC3s. Previous studies have reported that IL-1β, IL-23 and IL-18 are major inducers of IL-22 from ILC3^41,42^ (Fig. 3E). Therefore, we tested which of these cytokines from IEC are responsible for UroA-induced IL-22 generation from ILC3 (Fig. 3E). We used well-known AHR ligands [(6-Formylindolo[3,2-b]carbazole (FICZ), 2,3,7,8-Tetrachlorodibenzodioxin (TCDD)] as controls. As shown in Fig. 3F, G, UroA treatment did not induce IL-1β or IL-23 in WT organoids. However, UroA treatment significantly induced IL-18 in WT organoids in an AHR-dependent manner (Fig. 3H). Interestingly, high-affinity AHR ligands FICZ or TCDD failed to induce IL-18, suggesting distinct activation patterns of UroA to induce IL-18 compared to other known AHR ligands (Fig. 3H). Similar to small intestinal organoids, we also observed that UroA-induced IL-18 in colon organoids is AHR-dependent (Fig. S9C). Additionally, our findings demonstrated that UroA induced IL-22 in co-cultures of WT-colon organoids with WT lamina propria (WT-colon organoid:WT-LP) and WT colon organoids with *Ahr*^*−/−*^ lamina propria (WT-colon organoid:*Ahr*^*−/−*^LP), mirroring the response seen in small intestinal organoids (Fig. S9D).

To elucidate the specificity of IEC-IL-18 in driving IL-22 production, we employed specific neutralizing respective antibodies to block IL-18, IL-23, and IL-1β signaling. As illustrated in Fig. 3I, UroA treatment resulted in IL-22 induction in WT-O:WT-LP co-cultures. Blocking IL-1β and IL-23 had no impact on UroA-mediated IL-22 production. Intriguingly, the induction of IL-22 by UroA from ILC3 was significantly reduced in WT-O:WT-LP co-cultures treated with an IL-18 neutralizing antibody (anti-IL-18 Ab) but not in WT-LP (Fig. 3J).

Importantly, UroA failed to produce IL-22 in *Ahr*^*−/−*^-O:*Ahr*^*−/−*^-LP co-cultures, but recombinant IL-18 induced IL-22 in *Ahr*^*−/−*^-O:*Ahr*^*−/−*^-LP co-cultures. These results suggest that IL-18 is responsible for the induction of IL-22 even in the absence of AHR (Fig. 3K). Collectively, these findings suggest that UroA-driven IL-22 induction is dependent on IL-18, while being independent of IL-1β and IL-23. As expected, UroA failed to produce IL-22 and IL-18 from *Ahr*^*−/−*^ intestinal organoids both at mRNA and protein levels (Figs. 3K–M, S10A), suggesting UroA-mediated IL-18 production from IECs is AHR-dependent. Next, we evaluated IL-18 at mRNA and protein levels in the colons of the mice that were subjected to DSS-induced colitis. As shown in Fig. S10B–D, UroA treatment led to upregulation of both IL-18 mRNA and protein levels in the colons of mice compared with the vehicle. IL-18 mRNA and protein levels in the colons of mice exposed to DSS are significantly upregulated corroborating with previous observations^27,43^. However, treatment with UroA downregulated DSS-induced upregulation of IL-18 (restoring to homeostatic levels) in the colons of colitis mice. These results were further confirmed by measuring IL-18 protein levels in the colons using ELISA (Fig. S10E). We postulate that the heightened levels of IL-18 produced during colitis may contribute to inflammatory damage. It is possible that the maintenance of homeostatic IL-18 by UroA is crucial for sustaining the integrity of the gut barrier that offers protection against colitis. In summary, these findings indicate that UroA distinctly triggers IEC-AHR-dependent IL-18 production, subsequently eliciting an IL-22 response from ILC3.

### UroA-mediated IL-22 production is dependent on IEC-IL-18 signaling

IL-22 is produced by several types of cells, including T helper type 1 (Th1), Th17, Th22, CD8^+^ T cells, γδ T cells, NKP46^+^ natural killer cells, neutrophils, lymphoid tissue inducer cells, and ILC3^33,44–46^. In the above experiments, we have utilized LP cells that contain many cell types that are capable of producing IL-22. To evaluate the source of UroA-mediated IL-22 production, we analysed LP cells and their IL-22 levels by flow cytometry in in vivo models. Our results suggested that UroA failed to induce IL-22 from other cell types listed in Figs. S11A–C, S12. Overall, our studies thus far indicated that UroA treatment leads to the induction of IL-22 from ILC3. Further, to examine the direct effects of UroA in ILC3, we used the MNK3 cell line (ILC3 cell line model) in our co-culture model system. MNK3 cell line expresses the same transcription factors and cytokines as mouse ILC3 and produces IL-22 in response to IL-1β and IL-23^47^. The MNK3 cells were co-cultured with intestinal organoids prepared from WT or *Ahr*^−/−^ mice and treated either with Vehicle or UroA (Fig. S13A). As with O:LP co-cocultures, UroA treatment induced IL-22 in WT-O:MNK3 co-culture but failed to produce IL-22 in co-cultures of *Ahr*^−/−^-O:MNK3 cells (Fig. S13B). As expected, UroA induced the expression of IL-18 in IECs (EpCAM^+^ cells) (Fig. S13C). However, in the presence of the AHR inhibitor CH223191, UroA failed to induce IL-18, indicating the AHR-dependent nature of UroA-mediated IL-18 production in IECs. Next, we examined IL-22 production in MNK3 cells within the WT-O:MNK3 cell coculture, incorporating either the AHR inhibitor CH223191 or specific neutralizing antibodies against IL-18, IL-1β, or IL-23, in conjunction with UroA (Fig. S13D). As shown in Fig. S13D, UroA treatment induced IL-22 production from MNK3 cells. Treatment with CH223191 or anti-IL-18 antibodies abrogated UroA-induced IL-22 production, indicating that the AHR–IL-18 cascade is essential for UroA-mediated IL-22 induction. As noted above, anti-IL-23 or anti-IL-1β antibodies did not have an impact on UroA-mediated IL-22 production from MNK3 cells. Thus, selective activation of the AHR-IL-18 axis by UroA specifically regulates IL-22 production from ILC3 cells.

IL-18 is also produced by immune cells such as macrophages and dendritic cells in addition to intestinal epithelial cells^48^. To validate the source of UroA-induced IL-18, we co-cultured WT organoids and LP cells together with Vehicle (0.5% DMSO) or UroA (25 μM). Expression of IL-18 in epithelial cells (EpCAM^+^) and total immune cells (CD45^+^), macrophages (CD45^+^F/480^+^) and dendritic cells (CD45^+^CD11c^+^) was determined. The flow cytometry gating strategy is provided in Fig. S14. UroA induced IL-18 expression only from EpCAM^+^ cells but failed to induce IL-18 from immune cells such as macrophages or dendritic cells (Fig. S13E). These results suggest that UroA induces IL-18 in IEC, but not from immune cells.

### UroA requires the IEC-AHR-NLRP6 axis to induce IL-18 from epithelial cells

**Fig. 4:**
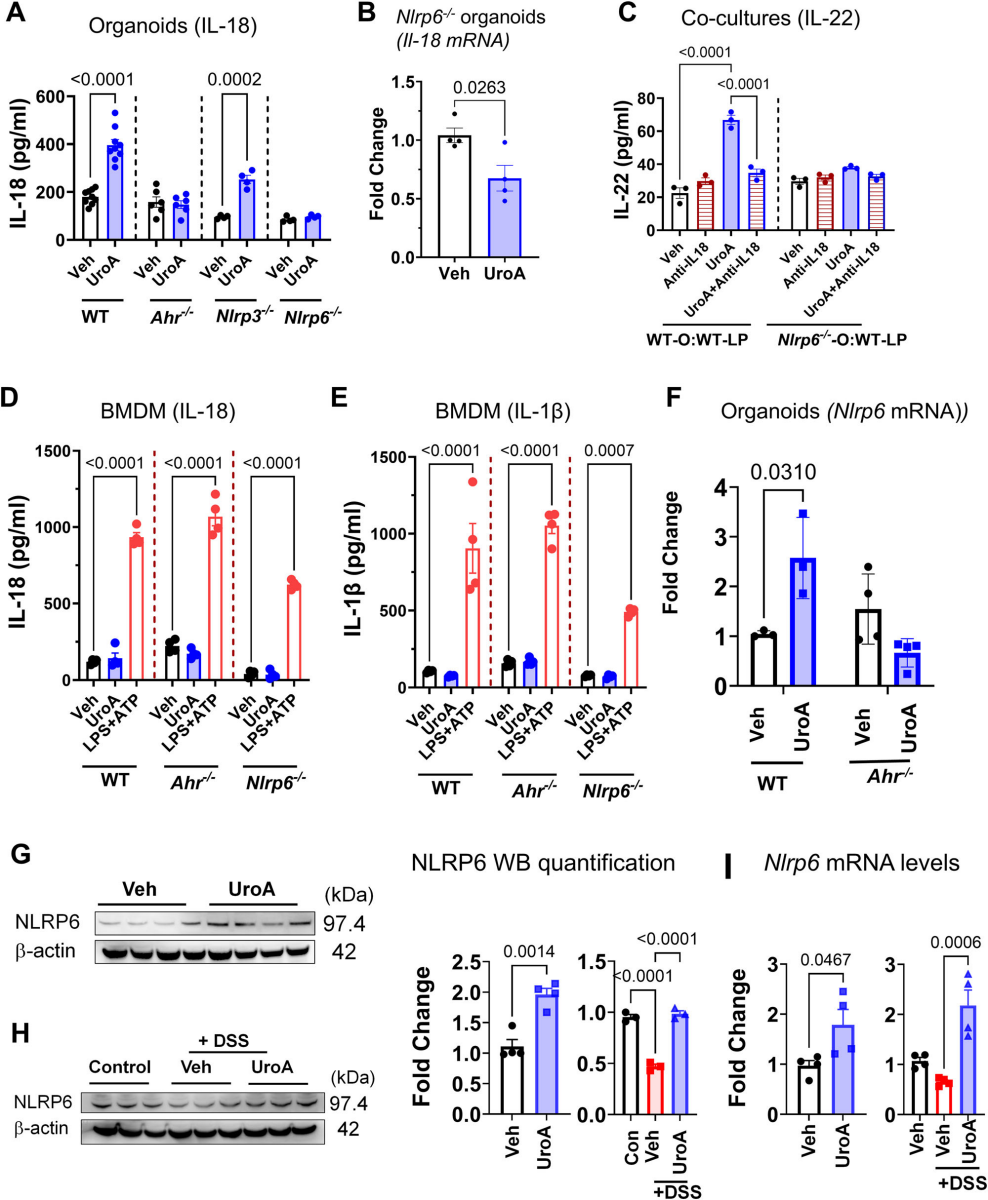
UroA requires the IEC-NLRP6-IL-18 axis to induce IL-22 from LP cells*.* **A** Intestinal organoids (*n* = 100 organoids) were prepared from WT, *Ahr*^−/−^, *Nlrp3*^−/−^ and *Nlrp6*^−/−^ mice and treated with vehicle (0.05% DMSO) or UroA (25 μM) for 72 h. The IL-18 levels were measured using standard ELISA methods (*n* = 4–9). **B** The fold changes in mRNA levels of *Il-18* were measured in *Nlrp6*^−/−^ intestinal organoids treated with vehicle or UroA by SYBR green RT-PCR methods. **C** WT-O:WT-LP or *Nlrp6*^−/−^-O:WT-LP cells were co-cultured and treated with neutralizing IL-18 antibody (20 ng/ml) in the presence or absence of UroA (25 μM). The IL-22 protein levels were measured in the supernatants by standard ELISA methods. **D**,** E** Bone marrow-derived macrophages (BMDMs) were prepared from WT, *Ahr*^−/−^ and *Nlrp6*^−/−^ mice and treated with vehicle (0.05% DMSO) or UroA (25 μM) or LPS (10 ng/ml)+ATP (5 mM) for 6 h and the levels of IL-18 (**D**) and IL-1β (**E**) were measured by ELISA. Statistics were performed using one-way ANOVA. Error bars, ±SEM; ns: not significant; ***p < 0.001; ****p < 0.0001. **F** WT and *Ahr*^*−/−*^ intestinal organoids were treated with vehicle (0.05% DMSO) or UroA (25 µM) for 72 h. The fold changes in mRNA levels of *Nlrp6* were determined by RT PCR method. Statistics were performed using two-way ANOVA. Error bars, ±SEM; ns: Not significant, *p < 0.05. **G–I** WT mice (C57BL/6, *n* = 5–7, 6–8-week-old) were either on normal water (control group) or subjected to 2.5% DSS in drinking water for 7 days, followed by 5 days of regular water. Both control and DSS-subjected mice were treated with either vehicle (1% CMC + 0.1% Tween 80) or UroA (20 mg/kg) orally every 48 h of intervals. Expression patterns of NLRP6 protein in the colons of control (**G**) and colitis (*n* = 4) (**H**) mice were measured by Western blots and quantified by using Image J software. (*n* = 3) (**I**) mRNA levels in colon tissues by SYBR green RT -PCR methods. (*n* = 4) Statistics were performed using either one-way ANOVA or a two-tailed unpaired *t*-test. Error bars, ±SEM; *p*-values < 0.05 are shown. All experiments were repeated at least three times using biologically independent replicates, yielding similar results. Source data are provided as a Source data file.

**Fig. 5:**
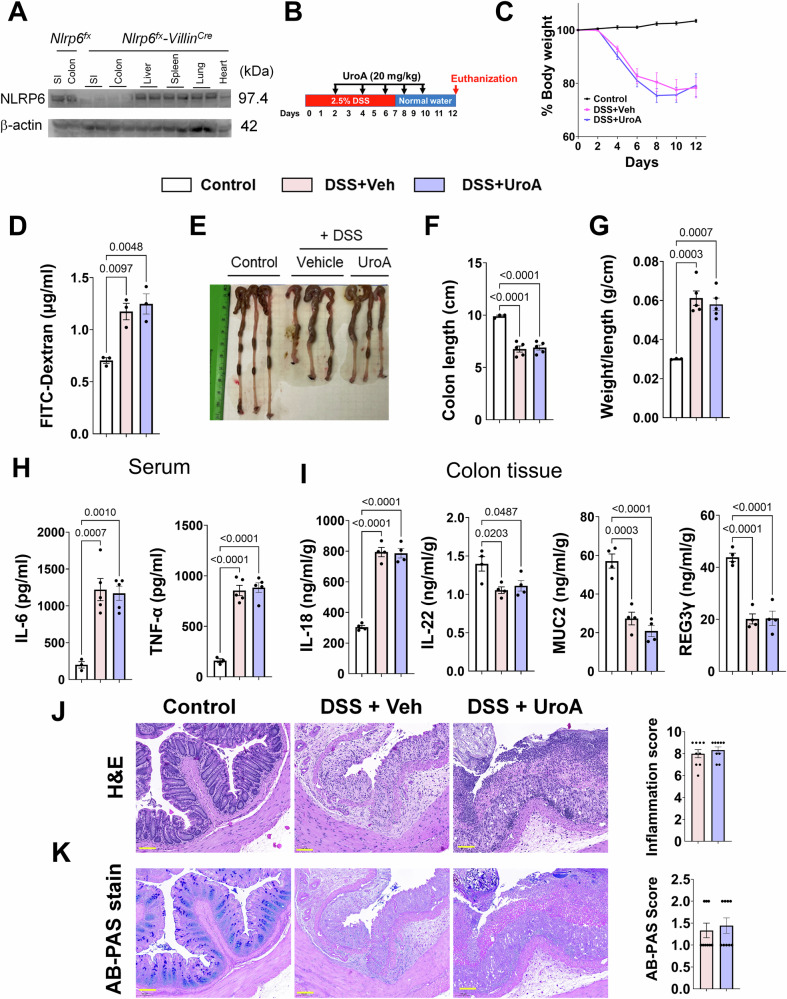
Intestinal epithelial NLRP6 is required for UroA-mediated protective activities against DSS-induced colitis. **A** Expression of NLRP6 was determined by Western blot analysis in the indicated tissues of *Nlrp6*^*fx/fx*^ (Cre negative) and *Nlrp6*^*fx*^*-Villin*^Cre^ mice. **B** *Nlrp6*^*fx*^*-Villin*^Cre^ mice (6–8 weeks old, *n* = 5–7 mice consisting of male (*n* = 3–4) and female (*n* = 2–3) mice per group) were either on normal water (control group) or subjected to 2.5% DSS in drinking water for 7 days, followed by 5 days of regular water. Both control and DSS-subjected mice were treated with either vehicle (1% CMC + 0.1% Tween 80) or UroA (20 mg/kg) orally every 48 h intervals. **C** Percent body weight loss. **D** Intestinal permeability was assessed by using the FITC-dextran intestinal permeability assay as described in the methods. **E** Gross images of colons are represented. **F** Colon lengths were measured. **G** Ratio of Colon weight/length is shown. **H** Serum IL-6 and TNF-α levels. **I** Colon tissue levels of IL-18, IL-22, MUC2 and REG3γ were determined using standard ELISA methods. **J**,** K** Microphotographs of hematoxylin and eosin (H&E) stained (**J**) and Alcian Blue-Periodic Acid Schiff (AB-PAS) stained (**K**) sections of colons are shown. Inflammation and AB-PAS scores were shown. Scale bar indicates 100 μm. Statistics were performed using one-way ANOVA. Error bars, ±SEM; ns: Not significant, *p*-values < 0.05 are shown. Source data are provided as a Source data file.

It is well-established that NLRP3 and NLRP6 inflammasome pathways are critical to induce IL-18 and IL-1β^15,49,50^. NLRP6 has been shown to be associated with maintenance of intestinal homeostasis^17^. To distinguish the involvement of NLRP3 or NLRP6 in UroA-induced IL-18 production, we utilized intestinal organoid cultures from *Nlrp3*^−/−^^51^ and *Nlrp6*^−/−^ mice^19^. As shown in Fig. 4A, UroA induced IL-18 in WT and *Nlrp3*^−/−^ organoids, suggesting that UroA-mediated IL-18 production is NLRP3-independent. As shown in Fig. 4A, UroA failed to induce secretion of IL-18 in *Ahr*^−/−^ and *Nlrp6*^−/−^ organoids, suggesting the requirement of the AHR-NLRP6 axis for UroA-mediated IL-18 induction. Similar results were also observed at the mRNA level, where UroA treatment did not induce *Il18* transcription in *Nlrp6*^−/−^ organoids (Fig. 4B). Next, we evaluated the role of IEC-NLRP6 on IL-22 production by ILC3s using WT or *Nlrp6*^*−/−*^ organoids:WT-LP co-culture systems. As expected, UroA induced IL-22, and blockade of IL-18 reduced the IL-22 production in WT-O:WT-LP co-cultures. However, UroA failed to induce IL-22 in *Nlrp6*^*−/−*^-O:WT-LP co-cultures, suggesting a requirement of IEC-NLRP6 for UroA-mediated IL-22 production (Fig. 4C). To define the specificity of UroA-mediated IL-18 from IECs, we investigated the effects of UroA on IL-18 release from bone marrow-derived macrophages (BMDMs). For this purpose, we prepared BMDMs from WT, *Ahr*^*−/−*^ or *Nlrp6*^*−/−*^ mice. LPS + ATP was used as a positive control to activate the inflammasome. As shown in Fig. 4D, E, UroA did not induce IL-18 or IL-1β in BMDMs. LPS + ATP treatment led to induction of IL-18 and IL-1β in BMDMs prepared from WT, *Ahr*^*−/−*^ or *Nlrp6*^*−/−*^ mice, suggesting that IL-18 or IL-1β production is not impaired in BMDMs of these knockout mice. Both NLRP3 and NLRP6 inflammasome pathways can independently induce IL-18 and IL-1β. The absence of NLRP6 does not hinder the NLRP3-dependent production of IL-18 and IL-1β. However, it does result in a reduction of the cytokines that are typically contributed by the NLRP6 pathways in *Nlrp6*^*−/−*^ BMDMs. It is possible that low expression of NLRP6 in macrophages and/or UroA’s inability to activate the NLRP3 inflammasome may be responsible for the failure of UroA-mediated upregulation of IL-18 in macrophages. UroA treatment induced the expression of NLRP6 in organoids in an AHR-dependent manner (Fig. 4F) as well as colons of WT mice (Fig. 4G). Significantly, UroA treatment successfully reinstated NLRP6 levels, counteracting the downregulation induced by DSS on NLRP6 expression both at protein (Fig. 4H) and mRNA levels (Fig. 4I). UroA-mediated activation of NLRP6 was evaluated by examining Caspase 1 activity in organoids. As shown in Fig. S15A, UroA activated caspase 1 activity similar to that of taurine (a well-known NLPR6 activator^17^) or Nigericin (NLPR3 activator) treatment. UroA failed to induce NLRP6 activity in the presence of the AHR inhibitor CH22319. Interestingly, the co-treatment with MCC950, a NLRP3 inhibitor, did not influence UroA-mediated caspase-1 activity, underscoring the specificity of NLRP6 in the regulation of UroA-mediated caspase-1 activity. As expected, UroA treatment failed to activate NLRP6 in *Ahr*^*−/−*^ and *Nlrp6*^*−/−*^ intestinal organoids (Fig. S15B). These results suggest that UroA triggers NLRP6-IL-18 in an IEC-AHR-dependent manner. Next, to evaluate whether IEC-NLRP6 is required for UroA protective activities against colitis, we generated *Nlrp6*^*fx*^*-Villin*^*Cre*^ mice, where the *Nlrp6* gene is deleted in IECs (Fig. 5A). As shown in Fig. 5, UroA treatment failed to protect *Nlrp6*^fx^*-Villin*^Cre^ mice against DSS-induced colitis compared to littermate control Cre-negative *Nlrp6*^*fx/fx*^ mice (Fig. S16), as evident from loss of body weight, increased intestinal permeability, shortening of colons, and increased systemic cytokines (Fig. 5B–H). Colonic IL-18, IL-22, MUC2 and REG3γ levels and H & E staining and mucin analysis supported similar results (Fig. 5I–K). To identify the putative AHR binding sites on the *Nlrp6* promoter, we screened 6 promoter sites-based JASPAR promoter screen program and performed Chromatin Immunoprecipitation-quantitative PCR (CHIP-qPCR). Two potential binding sites have been identified on the NLRP6 promoter region for AHR binding (Fig. S17). These promoter sites (Sequence 3 and Sequence 5) of *Nlrp6* were confirmed in both intestinal tissues and colon organoids using CHIP-qPCR.

### AHR-NLRP6-IL-18 axis is required for UroA-induced MUC2 and REG3γ

**Fig. 6:**
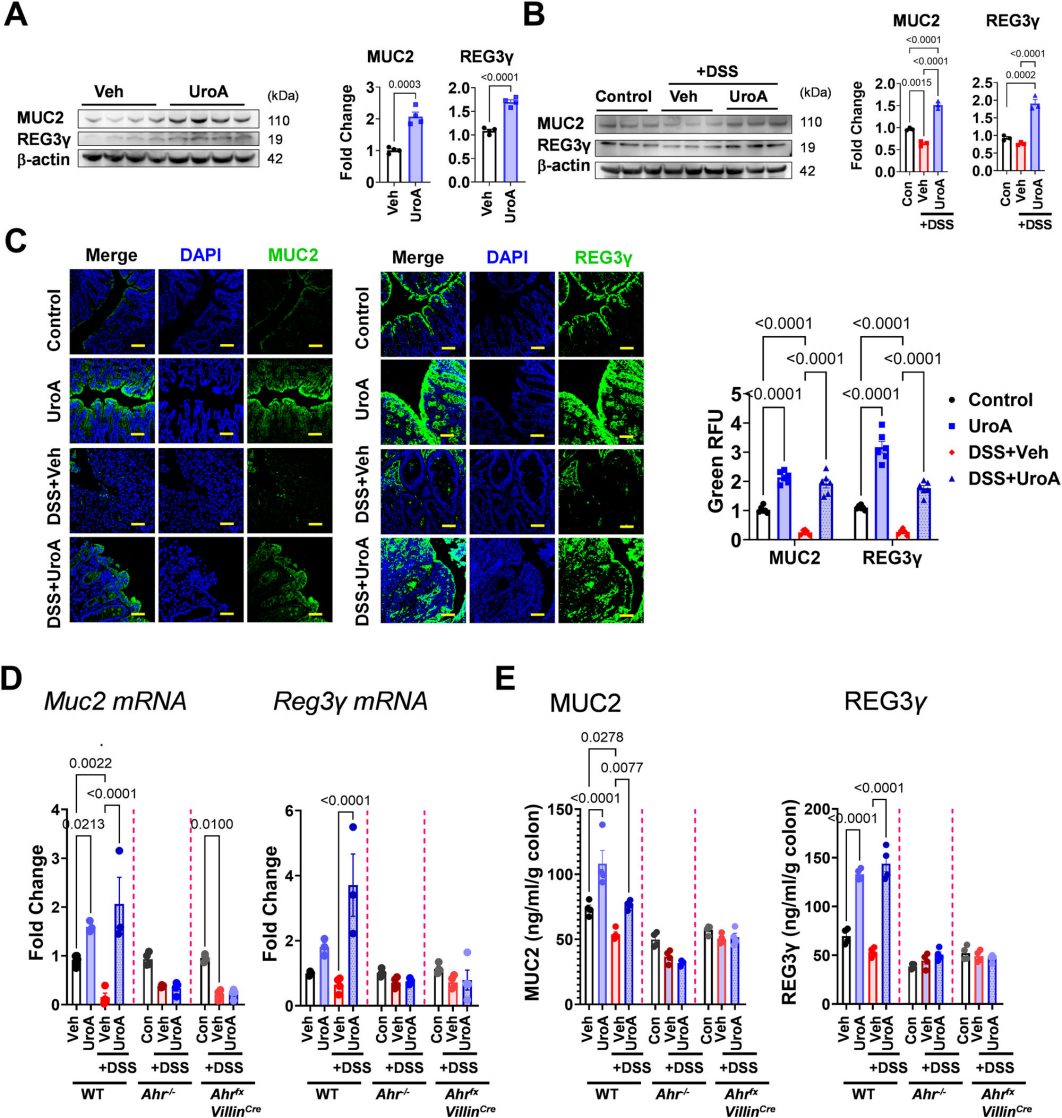
UroA upregulated MUC2 and REG3γ in an IEC-AHR-dependent manner. **A**,** B** WT mice (C57BL/6, *n* = 5–7, 6–8-week-old) were either on normal water (control group) or subjected to 2.5% DSS in drinking water for 7 days, followed by 5 days of regular water. Both control and DSS-subjected mice were treated with either vehicle (1% CMC + 0.1% Tween 80) or UroA (20 mg/kg) orally every 48 h intervals. Expression patterns of MUC2 and REG3γ proteins in the colon tissues of control (*n* = 4) (**A**) and colitis (*n* = 3) (**B**) mice were measured by Western blots and quantified by using Image J software. Statistics were performed using either one-way ANOVA or a two-tailed unpaired t-test. Error bars, ±SEM; *p*-values < 0.05 are shown. **C** Confocal images of colon sections stained with anti-MUC2 or REG3γ antibodies followed by secondary antibodies tagged with Alexa 488. The nucleus was stained with DAPI. The fluorescence images were captured using a Nikon A1R confocal microscope. The scale bar indicates 50 μm. The fluorescence intensity (*n* = ~20 cells) was measured per sample (*n* = 6). Statistics were performed by two-way ANOVA. Error bars, ±SEM. **D** The fold changes in mRNA levels of *Muc2* and *Reg3γ* in the colons of WT, *Ahr*^−/−^ and *Ahr*^fx^-*Villin*^Cre^ mice were determined by the SYBR green RT-PCR method. **E** Protein levels of MUC2 and REG3γ protein levels were determined in colon homogenates of WT, *Ahr*^−/−^, and *Ahr*^fx^-*Villin*^Cre^ mice using standard ELISA. Statistics were performed using one-way ANOVA. Error bars, ±SEM; *p*-values < 0.05 are shown. All experiments were repeated at least three times using biologically independent replicates, yielding similar results. Source data are provided as a Source data file.

The IL-18/IL-22 axis is known to induce anti-microbial peptides (AMPs, e.g., REG3γ) and MUC2 from IEC to maintain gut homeostasis^16,52–54^. To establish the relevance of this pathway in vivo under homeostatic or IBD conditions, we measured the expression of MUC2 and REG3γ in colon of wild type mice that were treated with vehicle or UroA under normal and colitis conditions. UroA treatment significantly upregulated the protein levels of MUC2 and REG3γ in the colons of WT mice and DSS-induced colitis mice, compared with vehicle treatment (Fig. 6A, B). Immunofluorescence staining studies also confirmed that UroA treatment upregulated MUC2 and REG3γ expression in the colons of naive and DSS-induced colitis mice (Fig. 6C). UroA-induced expression of *Muc2* and *Reg3γ* at mRNA and protein (Fig. 6D, E) levels in colons was confirmed. As predicted, UroA treatment failed to induce MUC2 and REG3γ in the intestines of *Ahr*^−/−^ and *Ahr*^fx^-*Villin*^Cre^ mice (Figs. 6D, E, S18).

To define whether UroA-mediated IL-18 is required for upregulation of REG3γ and MUC2, we used WT-O:WT-LP co-culture systems and IL-18 neutralizing approaches. As shown in Fig. S19A–C, UroA induced REG3γ and MUC2 in WT-O:WT-LP co-cultures, and treatment with anti-IL-18 antibody abrogated UroA-induced REG3γ and MUC2 both at mRNA and protein levels. Further, UroA failed to induce MUC2 or REG3γ in *Ahr*^−/−^ and *Nlrp6*^*−/−*^ organoids. Collectively, these results suggested the importance of the AHR-NLRP6-IL-18 axis for UroA-mediated upregulation of MUC2 or REG3γ (Fig. S19A, B). Previous reports suggest that IL-22 can induce IL-18 from IEC^53^. Therefore, we next asked whether the IL-22 signaling pathway is required for UroA-mediated IL-18 production, as depicted in Fig. S19D, recombinant IL-22 (rIL-22) or UroA induced IL-18 production in intestinal organoids from wild-type mice. However, when we blocked IL-22 signaling using anti-IL-22 antibodies, IL-18 production was reduced. Interestingly, UroA induced IL-18 production even in the presence of anti-IL-22 antibodies, suggesting that UroA mediated IL-18 production is independent of IL-22 signaling. Another AHR ligand, FICZ failed to induce IL-18 production, indicating that UroA distinctly activates AHR to produce IL-18. We further confirmed that UroA failed to induce IL-22, MUC2 and REG3γ when organoids from *Il18*^−/−^ mice were co-cultured with WT-LP (Fig. S20). These results support that UroA requires IL-18 to upregulate IL-22 from ILC3, and MUC2 and REG3γ from IECs.

### UroA requires IL-18 and IL-22 signaling pathways to protect against DSS-induced colitis

**Fig. 7:**
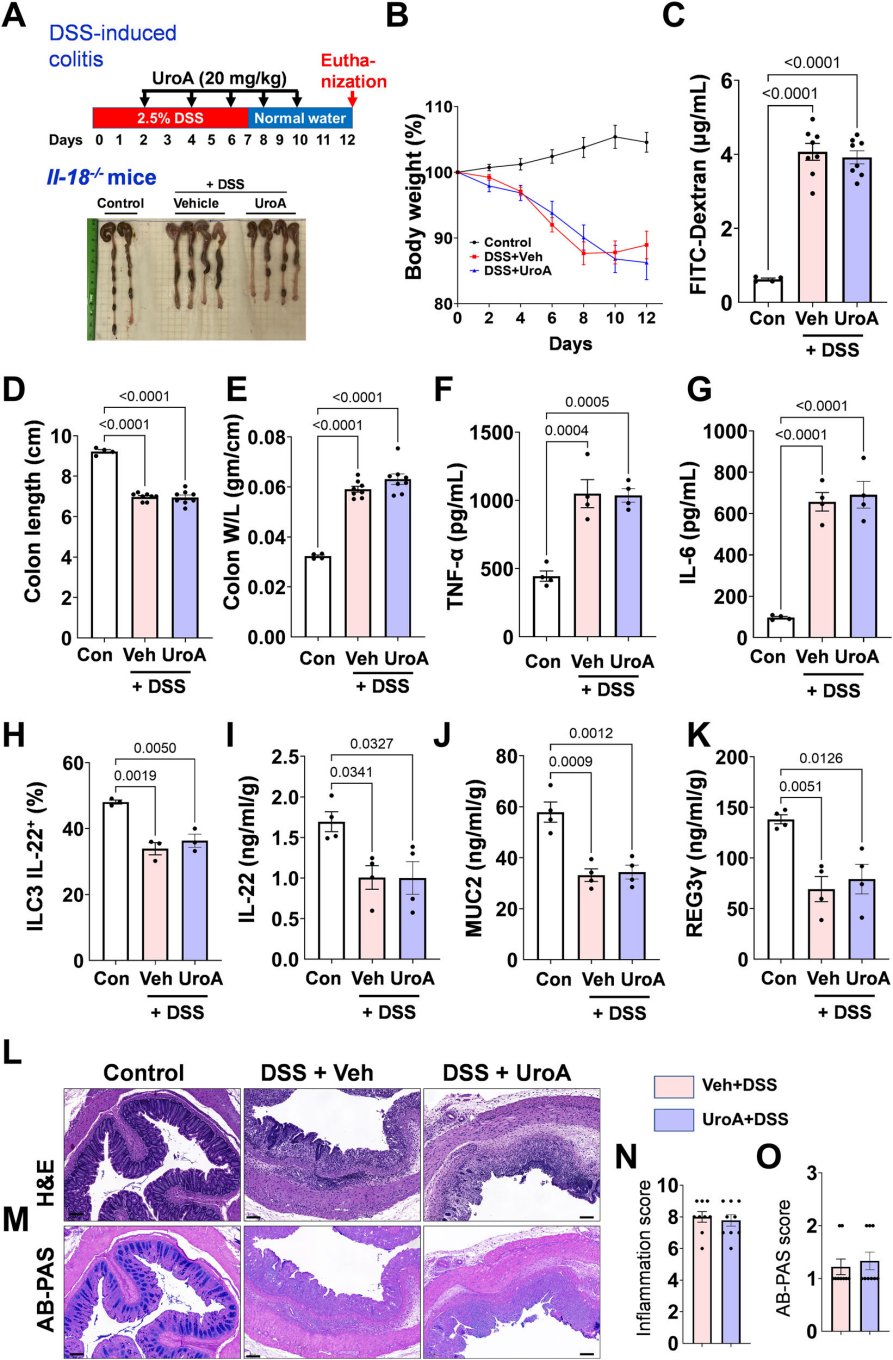
UroA failed to protect *Il-18*^*−/−*^ mice against DSS-induced colitis. **A** Schematic diagram of the DSS-induced colitis model and shows the frequency of UroA treatment. *Il-18*^*−/−*^ mice (8–10 week age old; *n* = 8, consisting of males (*n* = 4) and female (*n* = 4) mice per group) were subjected to 2.5% DSS in water for 7 days and shifted back to regular for another 5 days. Mice were treated with either Vehicle (1% CMC, 0.1% Tween-80) or UroA (20 mg/kg) orally every 48 h post DSS treatment. Control mice received normal water. All mice were euthanized at day 12 and characterized for the colitis phenotype. Gross images of colons were taken. **B** The percentage of body weight loss was evaluated. **C** Intestinal permeability was assessed by using the FITC-dextran intestinal permeability assay as described in the methods. **D** Colon lengths were measured. **E** Ratio of Colon weight/length is shown. **F**,** G** Serum cytokine levels, TNF-α (**F**) and IL-6 (**G**), were measured by standard ELISA. **H** Lamina propria cells were analyzed for IL-22^+^ ILC3s using standard flow cytometric procedures and followed the gating strategy as described in Fig. 2A. Percentage of IL22^+^ILC3 cells are shown. **I–K** Protein levels of IL-22, MUC2 and REG3γ were determined in colon homogenates using standard ELISA. **L–O** Microphotographs of hematoxylin and eosin (H&E) stained (**L**,** N**) and Alcian Blue-Periodic Acid Schiff (AB-PAS) stained (**M**,** O**) sections of colons are shown. Scale bar indicates 100 μm. Statistics were performed using one-way ANOVA. Error bars, ±SEM; *p*-values < 0.05 are shown. All experiments were repeated at least three times using biologically independent replicates, yielding similar results. Source data are provided as a Source data file.

To determine whether IL-18 signaling is required for the gut-protective effects of UroA against colitis, we evaluated UroA treatment in *Il18⁻/⁻* mice subjected to DSS-induced colitis. As shown in Fig. 7, DSS-induced severe colitis in *Il18*^*−/−*^ mice as indicated by loss of body weight, shortening of colons, increased intestinal permeability and increased systemic cytokines (Fig. 7A–G). Unlike in littermate (*ll18*^*+/+*^control mice (Fig. S21), UroA treatment failed to protect *Il18*^*−/−*^ mice from DSS-induced colitis. UroA also did not induce changes in frequency of IL-22^+^-ILC3, IL-22, MUC2 or REG3γ levels in the colons of *Il18*^*−/−*^ mice (Fig. 7H–K). It is also evident from H & E staining and mucin analysis that UroA treatment failed to restore the damaged colon epithelium and mucin levels in *Il18*^*−/−*^ mice (Fig. 7L–O). These studies indicate that UroA requires IL-18 signaling to restore gut homeostasis and barrier function. Next, we examined whether IL-22 is required for UroA-mediated protective activities against colitis using *Il22*^*−/−*^ mice. Our studies indicated that UroA treatment failed to protect *Il22*^*−/−*^ mice against colitis compared to littermates (Figs. S22, S23). The co-culture experiments of WT-O-*Il22*^*−/−*^ LP cells suggest that UroA induced IL-18 from epithelial cells independent of IL-22 (Fig. S24). These studies indicate that UroA-mediated IL-18 production from IEC in an IL-22-independent manner (Fig. S24A–C). UroA also failed to induce MUC2 and REG3γ in co-cultures of WT-organoids and *Il-22*^*−/−*^ lamina propria cells suggesting requirement of IL-22 for overall UroA activities (Fig. S24D, E). IL-22 receptor alpha 1 (IL22RA1) is a heterodimeric receptor for IL-22 and plays a protective role in colitis by promoting intestinal barrier integrity and tissue regeneration through several mechanisms^55^. We have generated mice that lack *Il-22ra1* in IECs (*Il-22ra1*^*fx/*^*Villin*^*Cre+*^ mice) to define IL-22-IL-22RA1 signaling pathway in UroA-mediated colitis protective activities. The *Il-22ra1*^*fx/*^*Villin*^*Cre+*^ mice and littermate *Il-22*^*fx/fx*^ mice were subjected to DSS-induced colitis and treated with UroA (Fig. S25). As expected, UroA treatment protected littermate *Il-22*^*fx/fx*^ mice from DSS-induced loss of body weight, shortening of colons, colon weight/length ratio, permeability and inflammatory cytokines (Figs. S25A, C–F, K). However, UroA treatment is partially rescued above parameters in *Il-22ra1*^*fx/*^*Villin*^*Cre+*^ mice against DSS-induced colitis (Figs. S25B,G–J, L). Importantly, UroA treatment did not induce IL-22, MUC2 and REG3γ in colons of *Il-22ra1*^*fx/*^*Villin*^*Cre+*^ mice compared to *Il-22*^*fx/fx*^ mice (Fig. S25M–P). However, UroA can downregulate IL-18 in both *Il-22ra1*^*fx/fx*^ and *Il-22ra1*^*fx/*^*Villin*^*Cre+*^ upon exposure to DSS Fig. S25M, N). Additionally, there is no significant reduction of colonic inflammation and goblet cell recovery in *Il-22ra1*^*fx/*^*Villin*^*Cre+*^ mice compared to *Il-22*^*fx/fx*^ mice (Fig. S25Q, R). It is possible that UroA requires both IL-18 and IL-22 signaling pathways to provide full protective activities against DSS-induced colitis.

### UroA elicits the IL-18/IL-22 axis in intestinal cells of IBD patients in AHR-dependent manner

**Fig. 8:**
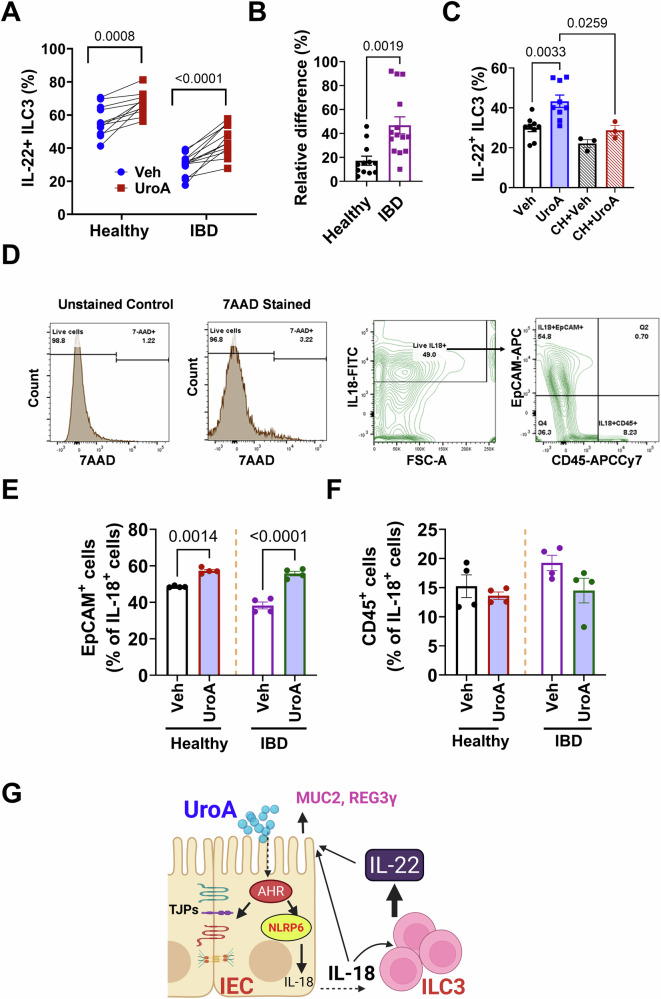
UroA induced IL-22 and IL-18 in intestinal cells isolated from IBD patients. **A** Single-cell suspensions were prepared from healthy and ulcerated tissue biopsies from IBD patients (*n* = 5) were treated with vehicle (0.05% DMSO) and UroA (25 µM) for 24 h, and IL-22^+^ ILC3 were determined using flow cytometry methods. Statistics were performed using two-way ANOVA pair-wise Sidak multiple comparison test; *p*-values < 0.05 are shown. **B** The original measurements are normalized by taking the relative difference from Veh to UroA, and then the logit transformation. Error bars, ±SEM; *p*-values less than 0.05 are shown. **C** Single cell suspension from IBD biopsies were treated Veh or UroA (25 μM) or CH-223191 (10 μM) or UroA (25 μM) + CH-223191 (10 μM). Statistics are performed using one-way ANOVA and Turkey’s multiple comparison test. Error bars, ±SEM; *p*-values less than 0.05 are shown. **D** Single cell suspensions were prepared from healthy and ulcerated tissues from IBD patients (*n* = 4 per group) and treated with Vehicle (Veh, 0.05% DMSO) and UroA (25 µM) for 24 h. Flowcytometric analysis of cell viability (7AAD^+^ cells) is shown. The live cells were gated for total IL-18^+^ followed by EpCAM^+^IL-18^+^ cells using standard flow cytometry analysis. **E**,** F** To quantify IL-18-producing cells, live cells were gated for IL-18^-^FITC^+^ and further analyzed for epithelial cells (EpCAM-APC^+^) and immune cells (CD45-APCcy7^+^). EpCAM^+^-IL-18^+^ and CD45 ^+^-IL18^+^ cells were determined. Statistics were performed using one-way ANOVA. Error bars, ±SEM; *p*-values less than 0.05 are shown. **G** UroA selectively activates the IEC-AHR-NLRP6 axis to induce homeostatic levels of IL-18 in IECs. IL-18 from IECs induces IL-22 to promote the induction of MUC2 and REG3γ to enhance gut barrier function, restore homeostasis and attenuate colitis. Created in BioRender. Ghosh, S. (2026) https://BioRender.com/sph8gt8. All experiments were repeated at least three times using biologically independent replicates, yielding similar results. Source data are provided as a Source data file.

To assess the translatability of our studies, we tested the effects of UroA on human intestinal biopsies. Human intestinal biopsies from both inflamed and non-inflamed areas were collected and single cell suspensions from these biopsies were prepared as described^56–58^. The single cell suspensions were treated with vehicle (0.05% DMSO) or UroA (25 μM) for 24 h. UroA treatment led to a significant increase in IL-22 in all the samples (Figs. 8A, S26). The relative difference of IL-22 between vehicle vs UroA-treated IBD samples was more prominent than healthy samples (Fig. 8B). Next, AHR specificity of UroA activities was tested using the AHR inhibitor CH-223191. As shown in Fig. 8C, UroA induced IL-22 from ILC3 cells, but failed to induce IL-22 in the presence of AHR inhibitor CH-223191. These results suggest that UroA-mediated IL-22 production from human primary ILC3 is AHR-dependent. To examine the status of UroA-induced IL-18, we also measured IL-18 production in these single-cell suspensions. The cell viability of a single cell suspension of biopsies was confirmed using 7-AAD staining (Fig. 8D) and gated for IL-18 levels from epithelial cells (EpCAM^+^) or immune cells (CD45^+^). As shown in Fig. 8E, F, UroA treatment induced IL-18 in EpCAM^+^ cells, but not in CD45^+^ immune cells, suggesting UroA-specific activation of AHR-IL18 in IEC compared to immune cells to produce IL-18.

In summary, UroA selectively activates AHR-NLRP6-dependent secretion of homeostatic levels of IL-18 in IECs, resulting in production of IL-22 from ILC3, leading to increased anti-microbial peptides and mucin levels to enhance gut barrier function, reduce unwarranted inflammation, and ultimately ameliorate IBD pathogenesis (Fig. 8G).

## Discussion

The AHR is a ligand-dependent transcription factor and is well-known to respond to structurally diverse exogenous and endogenous ligands that include gut microbial metabolites. Activation of AHR leads to a variety of ligand- and cell type- specific activities controlling many pathophysiological processes, including drug metabolism, inflammation, regulation of T cell differentiation, and gut barrier integrity^59,60^. For example, hyper-activation of AHR by dioxins (environmental toxins) such as TCDD causes immunotoxicity, neurodevelopmental abnormalities, disruption of steroid hormones and reproductive functions, and possible carcinogenesis^61,62^. In contrast, activation of AHR by some dietary ligands, such as indoles and flavonoids, exerts beneficial physiological functions^63,64^. Recent studies support the concept of selective AHR modulators (SAhRMs), where different AHR-ligands exhibit variable organ-, tissue- and cell type- specific activities^65–67^. Here, we showed that UroA activates IEC AHR to promote NLRP6-IL-18 signaling events to maintain gut homeostasis both at physiological and IBD conditions (Fig. 8G).

Though the importance of AHR signaling in gut barrier functions and regulation of the mucosal immune system has been documented^68–70^, its role in gut homeostasis and ligand-dependent downstream signaling patterns is still not completely understood. Here, we demonstrated that AHR can be activated by UroA in IECs to offer protection against colitis. Although UroA effectively inhibited LPS-induced inflammatory cytokines in macrophages, the UroA-AHR signaling cascade in macrophages does not seem to be essential to mitigate colitis, suggesting the existence of cell- and ligand-specific AHR activation mechanisms. These results emphasize that AHR may possess ligand-dependent, unique and distinct active downstream targets in epithelial cells and immune cells.

In this study, we demonstrated that the UroA activated AHR-dependent NLRP6-IL-18 pathways in IECs to induce IL-22 from ILC3. IL-22 plays an important role in gut barrier functions by increasing the number of goblet cells and intestinal epithelial homeostasis^33^. It was shown that AHR activation by microbial metabolites can stimulate ILC3 and CD4^+^ T cells to produce IL-22^36,71^. The therapeutic effects of AHR ligands such as FICZ against colitis are partly dependent upon the induction of IL-22^7^. To our surprise, UroA treatment failed to induce IL-22 in *Ahr*^*fx*^*Villin*^*Cre*^ mice, despite having normal AHR expression in lymphocytes, including ILC3 of these mice. The findings from WT-O:*Ahr*^*−/−*^ LP co-cultures further showed that UroA induced IL-22 from ILC3, even in the absence of AHR expression on ILC3, suggesting IEC-AHR dependency for IL-22 production. In search of the UroA-induced mediators responsible for IL-22 production, we identified that UroA, but not TCDD, significantly induced the expression of IL-18 in an AHR-dependent manner. These findings reveal unique signaling activation mechanisms of UroA when compared to established AHR ligands in IECs.

Next, we explored the UroA-induced regulators upstream of the IL-18 signaling cascade. Numerous studies have established the role of inflammasome pathways in the generation of IL-18^15,49,50^. The NLRP6-dependent pathway is especially critical for IL-18 production in intestinal epithelial cells. The dual roles for the NLRP6-IL-18/IL-22 axis have been reported both as a pro-inflammatory and a cytoprotective mediator in intestinal epithelial cells based on the time of activation and levels of production^27,52,72–77^. *Nlrp6*^−/−^ and *Il18*^−/−^ mice are also more susceptible to DSS-induced colitis, indicating their importance in maintaining gut homeostasis^53,78–81^. Our studies unveiled that UroA-induced IL-18 is dependent on NLRP6, but not on NLRP3. Additionally, co-culture (*Ahr*^*−/−*^*-*O or *Nlrp6*^*−/−*^*-*O:WT-LP) experiments suggested that UroA activation of the AHR-NLRP6-IL-18 axis in IECs is important for IL-22 production of ILC3. IL-18 is produced by several immune cells, including macrophages and dendritic cells, upon inflammasome activation. However, our data clearly show that UroA activates IEC-AHR to induce IL-18 specifically in intestinal epithelial cells, rather than in immune cells. Macrophages generally express much lower levels of NLRP6 compared with NLRP3, whereas IECs display substantially higher NLRP6 expression^13,14,82–84^. Therefore, it is likely that UroA does not promote IL‑18 production in macrophages due to their low levels of NLRP6 expression. In contrast, UroA induces IL‑18 in IECs, where NLRP6 expression is adequate to support IL‑18 production within the gastrointestinal tract.

One unanswered question revolves around how UroA-mediated AHR activation can induce NLRP6 in IECs while excluding NLRP3. As AHR functions as a transcription factor, it remains unclear whether UroA induces a specific conformational change in AHR or its associated partners, enabling binding to the NLRP6 promoter and thereby enhancing NLRP6 expression. Alternatively, the UroA-AHR signaling pathway might regulate the stability of NLRP6. Previous studies by Mukherjee et al. have demonstrated that NLRP6 activity is governed by the ubiquitination of K-63, facilitating ASC recruitment and potentially contributing to inflammasome oligomerization^85^. This study demonstrated that CYLD Lysine 63 Deubiquitinase (Cyld), a deubiquitinating enzyme (DUB), deubiquitinates K-63 of NLRP6, resulting in the negative regulation of NLRP6 inflammasome activation and preventing excessive IL-18 levels in the colonic mucosa. UroA-mediated activation of AHR may modulate the ubiquitination status of NLRP6. Further investigations are necessary to explore these possibilities and determine whether UroA treatment alters the ubiquitination status of NLRP6.

Earlier studies reported elevated IL-18 levels in IBD patients^74,86^, and loss-of-function IL-18 SNPs have been shown to increase the risk of Crohn’s disease in humans^87,88^. It was shown that excess IL-18 worsens DSS colitis in mice^27^, whereas administering recombinant IL-18 (rIL-18) before disease onset is protective^75^. Similarly, treating with rIL-18 rescued the mice that lack apoptosis-associated speck-like protein containing a CARD (*Asc*^*−/−*^ mice that to produce IL-18) from DSS-induced colitis^17^. Although neutralizing IL-18 reduces acute TNBS colitis, it does not improve chronic colitis^89^, highlighting the unresolved mechanisms underlying IL-18’s dual roles (pro-inflammatory vs anti-inflammatory) in gut barrier regulation and IBD. Interestingly, our data show that despite elevated levels of IL-18 in colons of *Nlrp6*^*fx*^*-Villin*^*Cre+*^ *and Nlrp6*^*fx*/*fx*^ mice subjected to DSS, IL-22 remained low at day 12 during recovery in vehicle-treated animals. It is possible that increased inflammatory IL-18 seen in DSS+vehicle-treated mice likely originates from infiltrating immune cells through NLRP3-dependent pathways^90^, which may drive goblet cell depletion and hinder epithelial barrier recovery/repair^91^ and reduce IL-22. However, UroA treatment reduced inflammatory IL-18 and increased IL-22 during recovery in *Nlrp6*^*fx*/*fx*^ mice, but not in *Nlrp6*^*fx*^*-Villin*^*Cre+*^. UroA appears to suppress immune-cell–derived inflammatory IL-18 in an NLRP3-dependent manner while promoting NLRP6-dependent IL-18 from IEC. Previous studies support this idea, showing that UroA treatment reduces LPS‑induced inflammatory cytokines in BMDMs^22^ and inhibits NLRP3 inflammasome activation in LPS‑primed BV2 cells^92,93^. Furthermore, unpublished data indicate that UroA treatment decreases LPS + ATP-induced IL-18 and IL-1β in BMDMs in an AHR-dependent manner. Together, these findings suggest that the high IL-18 observed in DSS-treated colons potentially reflects NLRP3-driven inflammation in immune cells, whereas UroA promotes NLRP6-dependent IL-18 in IECs and suppresses NLRP3-mediated IL-18 in immune cells. This dual action positions UroA as a unique metabolite that enhances IL-18/IL-22–mediated epithelial repair. Future studies are warranted to distinguish the roles of epithelial- versus immune-cell–derived IL-18 across inflammation and recovery using cell-specific IL-18 knockout models. It is also important to measure IL-22 levels at multiple time points (during DSS exposure and recovery) and correlate them with IL-18, other cytokines (such as IL-1β and IL-23), and the extent of tissue damage and recovery in both vehicle- and UroA-treated groups.

Our studies indicated that UroA treatment led to an increase in the MUC2 and REG3γ in intestinal organoids in an AHR-NLRP6-IL-18 axis-dependent manner. MUC2 is a secreted gel-forming mucin and forms the intestinal mucus barrier that physically separates the bacteria from the host epithelial layer^94^. Mice that lack of MUC2 exhibited increase susceptibility to IBD and spontaneously develop colitis^95–97^. Increased IL-22 is known to induce the innate antimicrobials, including defensins, Reg family molecules, and S100 proteins^98,99^. In addition, IL-22 protects goblet cells during colitis and induces the production of mucins to enhance gut barrier function^100^. Here, we demonstrate that UroA-induced IL-18 is sufficient to increase the MUC2 and REG3γ production in intestinal organoids. Nevertheless, when subjected to co-cultures (O:LP cells), UroA treatment resulted in significantly increased levels of MUC2 and REG3γ compared to organoids alone. This suggests a synergistic effect between UroA-induced IL-18 and IL-22 in promoting MUC2 and REG3γ production.

It is established that IL-22 has the capability to induce IL-18 in IECs^53,101^. Conversely, IL-18 was shown to drive ILC3 proliferation and promote IL-22 production via NF-κB^101,102^. In agreement with these studies, our data suggest that rIL-22 led to the induction of IL-18. Interestingly, treatment with UroA upregulated IL-18 independent of IL-22 signaling in IECs. FICZ, a well-known AHR ligand, failed to upregulate IL-18 in IECs, indicating distinct activity patterns of UroA and FICZ. Further, we confirmed the importance of IL-18 using organoids prepared from *Il18*^*−/−*^ mice, where UroA treatment failed to induce MUC2 and REG3γ as well as IL-22 in co-cultures (*Il18*^*−/−*^ O: WT-LP cells). UroA treatment also failed to mitigate colitis in *Il18*^*−/−*^ mice, suggesting a requirement of IL-18 for UroA-mediated protective activities. Most importantly, UroA treatment also failed to induce IL-22, MUC2 and REG3γ in *Il18*^*−/−*^ mice, suggesting the importance of IL-18 for UroA-mediated barrier protective activities. The importance of IL-22 signaling in IBD has been extensively studied^103–105^. Our pre-clinical results from *ll-22/-* and *Il-22ra1*^*fx/*^*Villin*^*Cre+*^ mice suggest that IL-22-IL-22RA1 signaling pathways are critical for UroA-mediated protective barrier functions (e.g., improved MUC2 and Reg3g levels) against colitis. Our data also support that the IL-22 levels are significantly reduced in ulcerated intestinal tissues compared to healthy tissues. UroA treatment of single cell suspensions of human biopsies led to an increase in IL-22 and IL-18 in ILC3 and IECs, respectively. Overall, an increase in these protective cytokines in ex vivo human cultures suggests the potential benefits of UroA in improving gut health.

**Fig. 9:**
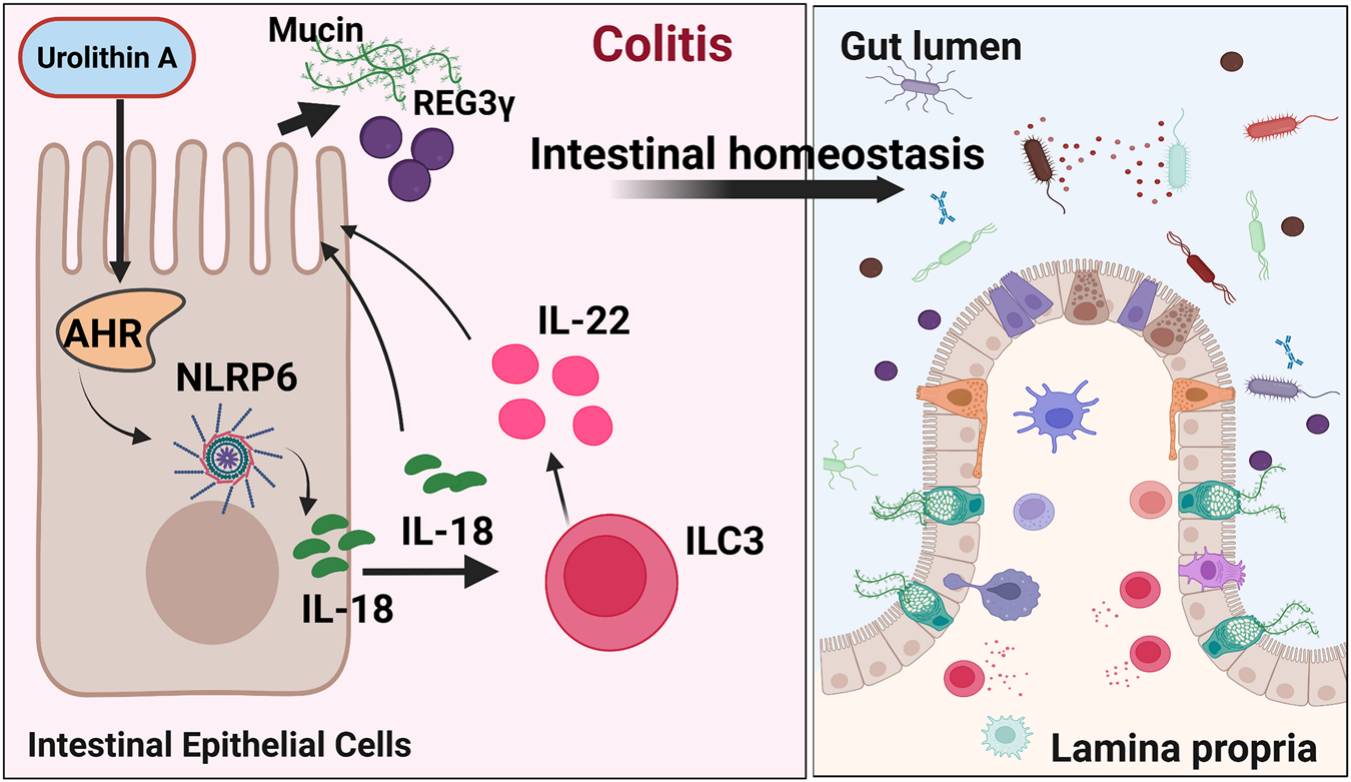
Proposed mechanism by which the UroA modulates intestinal immunity. UroA induces IL-18 by activating AHR-NLRP6-dependent manner. pathway. UroA-induced homeostatic levels of IL-18 from IECs promote IL-22 production from ILC3s, which in turn induces antimicrobial peptide REG3γ and mucin MUC2 expression. This coordinated response enhances epithelial barrier integrity, limits inflammation during colitis, and promotes maintenance of intestinal homeostasis. Created in BioRender. Ghosh, S. (2026) https://BioRender.com/sq0jb9y.

In summary, our studies suggest that specific activation of the IEC xenobiotic receptor AHR engages the inflammasome (NLRP6)-IL-18 pathway to induce IL-22 from ILC3. This signaling cascade enhances expression of MUC2 and REG3g in IECs, thereby maintaining gut epithelial integrity and mucosal homeostasis (Fig. 9). Notably, when compared to other AHR ligands, UroA distinctly activates AHR-NLRP6, orchestrating the regulation of IL-18/IL-22 and thereby enhancing gut homeostasis in both physiological and pathological IBD conditions. Existing research indicates lower levels of endogenous AHR ligands and AHR expression in IBD patients compared to their healthy counterparts^7,8,106,107^. We suggest that individuals with reduced beneficial AHR ligands/AHR activities in the context of IBD may face challenges in maintaining gut homeostasis and could be more susceptible to severe disease. Consequently, comprehending the distinct mechanisms of AHR activation and its downstream partners that govern gut barrier functions is crucial for IBD control. This selective activation of intestinal epithelial cell (IEC)-AHR by UroA holds promise for restoring gut homeostasis and mitigating inflammation in IBD through the IEC-NLRP6-IL-18/IL-22 signaling pathways. The outcomes of these studies may pave the way for microbial metabolite-based therapeutic interventions, offering improved health conditions for IBD patients without inducing systemic toxicity.

### Limitations of the study

These studies represent the microbial metabolite-activated AHR-NLRP6 axis in intestinal epithelial cells (IECs), which is responsible for IL-18 production and leads to the production of IL-22 from ILC3. Nevertheless, the molecular mechanisms underlying the AHR-dependent activation of NLRP6 remain unidentified. The specific activation of NLRP6 by UroA-AHR signaling, leading to the induction of IL-18 without affecting IL-1β, is not yet comprehended. We hypothesize that UroA selectively triggers AHR-dependent signaling, activating caspases or other unknown pathways that are specific to the upregulation of IL-18. The perplexing ability of UroA to downregulate inflammatory IL-18 in colitis conditions and restore it to normal/homeostatic levels remains elusive. Earlier research has highlighted the dual roles of IL-18 in colitis, demonstrating both pro-inflammatory and anti-inflammatory effects depending on the timing of administration and IL-18 levels. In future investigations, introducing rIL-18 or an anti-IL-18 antibody during disease progression will help elucidate whether UroA can mitigate inflammatory IL-18 during colitis development. Moreover, uncertainties persist regarding the specific type of intestinal epithelial cells responsible for UroA-mediated IL-18 production. Additionally, it remains unclear whether UroA can directly impact the proliferation and differentiation of stem cells, particularly goblet cells, to enhance mucin production and bolster gut barrier function. Further, how uniquely UroA regulates AHR and impacts downstream signaling cascades compared to known AHR ligands is still under investigation. Addressing these questions will be integral to advancing our understanding of UroA’s mechanisms and its potential therapeutic implications.

## METHODS

### Mice

Wildype (C57BL/6, Strain #000664), *Il-18*^*−/−*^ mice (B6.129P2-*Il18*^*tm1Aki*^/J, Strain #004130), were obtained from Jackson Laboratory and *Ahr*^−/−^ (C57BL/6-*Ahr*^*tm1.2Arte*^, Model: 9166) mice were procured from Taconic Laboratories. *Ahr*^fx^ mice (*Ahr*^*tm3.1Bra*^/J; Strain #006203, from Jackson Laboratory) were crossed with Villin-Cre (B6.Cg-Tg(Vil1-cre)997Gum/J; Strain #004586, from Jackson Laboratory) and LysMCre mice (B6.129P2-*Lyz2*^*tm1(cre)Ifo*^/J, Strain #004781, from Jackson Laboratory) at the University of Louisville animal facility to generate *Ahr*^fx^-*Villin*^Cre^ and *Ahr*^fx^-*LysM*^Cre^ mice by cell-specific deletion of these genes using Cre/lox methodologies. *Nlrp6*^fx^ mice (Provided by Dr. Daniel Mucida, Rockefeller University, USA, by signing MTA with original creator P. Rosenstiel, University Hospital Schleswig Holstein **·** Campus Kiel, Germany)^108^ were crossed with Villin-Cre (B6.Cg-Tg(Vil1-cre)997Gum/J; Strain #004586, from Jackson Laboratory). Mice were bred at our animal facility to generate experimental *Nlrp6*^fx^ -*Villin*^Cre^ animals. *Il-22*^*−/−*^ mice^109,110^ were provided by Dr. Misty Good, University of North Carolina. *Il-22ra1*^*fx/fx*^^111^ *and Il-22ra1*^*fx/*^*Villin*^*Cre+*^ mice^112^ were provided by Dr. Pawan Kumar from Stony Brook University. Mice at 6–8-week age old (both genders) were utilized for colitis experiments or organoid preparation. For each genotype, male and female mice were always equally distributed to each experimental group. In some cases, control and experimental animals were bred separately. To validate the observed phenotypes, littermate controls were included alongside the experimental animals. These littermate controls were generated by crossing heterozygous mice. Vehicle and treatments group animals were housed in separate cages. For treatment allocation, animals were randomized based on genotype to avoid selection bias and to ensure that each experimental group was balanced in terms of genetic background. At the experimental endpoint, mice were euthanized by CO_2_ asphyxiation followed by cervical dislocation. Mice were maintained in a temperature-controlled (ambient temperature between 70–72 °F with a relative humidity range of 30% to 70% and specific pathogen-free (SPF) barrier conditions with alternate 12 h cycles of dark and light. All the experimental studies were performed under approved protocols from the Institutional Animal Care and Use Committee (IACUC), University of Louisville, Louisville, KY, USA and adhered to guidelines in the Guide for the Care and Use of Laboratory Animals of the National Institutes of Health (NIH).

### DSS-induced acute colitis model

For DSS-induced colitis, experimental mice received 2.5% DSS (36,000–50,000 MW, MP Biomedicals) in their drinking water for 7 d, followed by 5 d of water without DSS. Control animals received regular water without DSS for the entire period. Mice were monitored regularly for body weight and sacrificed on day 12 for all analysis. Mice were randomly divided into groups and received the oral treatment of either vehicle (1% CMC and 0.1% Polysorbate 80) or UroA (20 mg/kg) on the alternate day starting from day 2 of DSS treatment. UroA was prepared in 1% CMC and 0.1% Polysorbate 80. In other cohort, mice without DSS treatment were orally treated with either vehicle (1% CMC and 0.1% Polysorbate 80) or UroA (20 mg/kg) on the alternate days starting from day 0.

### Assessment of colitis phenotype and tissue collection

Mice were weighed on alternate days. At the experimental endpoint, mice were euthanized with CO_2_ asphyxiation followed by cervical dislocation. After euthanasia, the mice's blood was collected using cardiac puncture. The colons were taken out after dissection, and the gross colon images were captured. The colons were cleaned with 1X PBS, and the fat tissues were removed. Colon length and colon weight were measured further. Colons were collected in complete RPMI medium (containing 10% fetal bovine serum (FBS, Gibco) and 1X penicillin-streptomycin solution (100 U/ml penicillin, and 100 µg/ml streptomycin) for lamina propria cell isolation. In a separate set of experiments, portions of the colon were either snap-frozen in liquid nitrogen and stored in −80 °C on in RNA later or 10% phosphate buffered saline formalin until further analysis.

### In vivo intestinal permeability determination

Intestinal permeability was measured by FITC-dextran (MW 4 kDa FD4, Sigma-Aldrich), as described elsewhere^28^.

### Histopathology

After fixation, colon tissues were processed for histopathology and further stained for both Hematoxylin and Eosin (H&E) and Alcian Blue-Periodic acid–Schiff (AB-PAS) staining as described previously^28^. The colon sections after H&E staining were used for histological scores as described by Erben et al.^113^. AB-PAS scoring was performed after counting the percentage of positive cells/field and was scored as described in Vadivazhagan et al.^114^. For AB-PAS staining, the percentage of positive cells/field 75 to 100% cells were scored 5, 50 to 75% cells were scored 4, 25 to 50% cells were scored 3, 5 to 25% cells were scored 2 and 0 to 5% cells were scored 1.

### Myeloperoxidase (MPO) activity

MPO activity of colon tissue homogenate was evaluated using Myeloperoxidase (MPO) Activity Assay Kit (Abcam) as per manufacture’s instruction, and MPO units were normalized with per mg colon tissue. Briefly, colon tissue was homogenized in 0.5% (w/v) hexadecyltrimethylammonium bromide (H6269; Sigma-Aldrich, USA) prepared in 50 mM PBS (pH 6.0). The homogenate was subjected to three freeze–thaw cycles followed by 10–15 s of sonication to obtain a uniform suspension. The resulting mixture was centrifuged at 13,000 × *g* for 20 min at 4 °C, and the supernatant was collected. An aliquot of the supernatant (10 µl) was added to 50 mM potassium phosphate buffer (pH 6.0) containing 0.167 mg/ml o-dianisidine (Sigma-Aldrich, USA) and 0.0005% H₂O₂ (Sigma-Aldrich, USA). Absorbance at 450 nm was measured (BMG LABTECH) at 2-min intervals. Myeloperoxidase (MPO) activity was calculated using the change in absorbance over time, applying the formula [ΔA(t₂ − t₁)]/Δmin × (1.13 × 10^−2^), where one unit (U) of MPO corresponds to the degradation of 1 µmol of H₂O₂ per minute (molar extinction coefficient: 1.13 × 10^−2^). MPO activity was normalized to tissue weight and expressed as units per milligram of tissue.

### Immunofluorescence

The paraffin section slides were permeabilized and stained with either AHR or MUC2 or REG3γ specific primary antibody (1:100 dilution) followed by Alexa flour 488 secondary antibody (1:500 dilution) as described previously^28,115^. Nucleus were stained with DAPI. List of the antibodies with working dilutions is provided in Table S1.

### Bone marrow derived macrophages (BMDMs) preparation

Mouse BMDMs were prepared and evaluated for the anti-inflammatory properties of UroA on BMDMs, as mentioned earlier^28^. Briefly, mice were euthanized by CO₂ asphyxiation, followed by rinsing in 70% ethanol. Bone marrow was harvested from the tibias and femurs, and the cells were plated at a density of 2 × 10^6^ cells/ml in high-glucose DMEM (HyClone) supplemented with 10% FBS, 1% glutamine, 1× penicillin–streptomycin, and 50 ng/ml mouse M-CSF (R&D Systems, Minneapolis, MN). Cells were cultured for 7 days to allow differentiation.

### Enzyme-linked immunosorbent assay (ELISA)

TNF-α and IL-6 levels in serum cytokines and BMDM supernatants were measured using mouse TNF-α and IL-6 specific ELISA kits (Biolegend). The levels of MUC2, REG3γ, IL-22, IL-18, IL-1β and IL-23 were measured in either colon homogenate or supernatant from organoid culture/ organoid-lamina propria culture. Mouse-specific MUC2 and REG3γ ELISA kits were purchased from MyBioSource, and the rest were procured from Biolegend. The ELISA for the experiments was processed following the standard protocol provided by the manufacturer.

### *Citrobacter rodentium*-induced colitis

*C. rodentium* is a gift from Dr. Gabriel Núñez from the University of Michigan, Ann Arbor, MI. For *C. rodentium*-induced colitis, experimental mice were gavaged 200 μL of 5 × 10^9^ inoculum at day 0. Control animals received PBS. Mice were monitored regularly for body weight and sacrificed on day 12 for all analysis. All colitis group mice were randomly divided into groups and received the oral treatment of either vehicle (1% CMC and 0.1% Polysorbate 80) or UroA (20 mg/kg) on the alternate day starting from day 0. UroA was prepared in 1% CMC and 0.1% Polysorbate 80.

### Immunoblotting

The mice colon tissues were homogenized and processed for western blotting as mentioned earlier^22,28,116^. The blots were probed with anti- AHR, IL-18, NLRP6, MUC2 or REG3γ antibodies. List of the antibodies with working dilutions are provided in Table S1.

### Quantitative real-time polymerase chain reaction (qRT-PCR)

Total RNA was prepared from colon tissues or organoids using either the RNeasy Mini Kit (QIAGEN) or Maxwell® 16 LEV simplyRNA tissue kits (Promega). Changes in expression of specific genes were evaluated as previously described^22,117^. List of the primers and sequences are provided in Table S1.

### Isolation of lamina propria lymphocytes and flow cytometry

Cells from mice lamina propria lymphocytes were isolated using the mouse Lamina Propria Dissociation Kit (Miltenyi biotec, USA) following manufacture’s protocol with the help of gentleMACS™ Octo Dissociator with Heaters (Miltenyi biotec, USA). Cells were further isolated by Percoll gradient described elsewhere^118^. To analyze the cytokine secretion of the ILC3s, lamina propria single-cell suspensions were cultured and stimulated at a density of 10^6^/ml in culture medium (RMPI, 10% fetal bovine serum (FBS) and 1% penicillin/ streptomycin) with IL-23 (R&D) (40 ng/ml), IL-1β (StemCell) (100 ng/ml) and Brefeldin A (Cell Signaling Technologies) (5 μg/ml) for 4 h at 37 °C. Cells were stained following flow cytometric methods as described elsewhere^118,119^. Briefly, cells were stained with appropriate fluorochrome-labeled anti-mouse CD45.2, CD127, CD90.2, Nkp46, CD4, KLRG1, CD3, CCR6, CD19, Ly-6G and Ly-6C, F4/80, CD5, TCR β, TCR γδ and the Live/Dead Fixable Yellow dead cell stain. Intracellular staining was performed using eBioscience™ Foxp3 / Transcription Factor Staining Buffer Set as per the manufacturer’s instructions with appropriate fluorochrome-labeled anti-mouse Eomes, GATA-3, RORγt, and IL22 Ab. To analyze IL-22 secretion from different cell types, cells were stained with appropriate fluorochrome-labeled anti-mouse CD45.2, CD3, CD4, CD8 TCR γδ and the Live/Dead Fixable Yellow dead cell stain. Intracellular staining was performed using True-Nuclear™ Transcription Factor Buffer Set (Biolegend, San Diego, CA, USA) as per the manufacturer’s instructions with appropriate fluorochrome-labeled anti-mouse IL22 Ab. The flowcytometric results were acquired using BD LSRFortessa. Data was analyzed using FlowJo software (Tree Star). List of the antibodies with catalog numbers is provided in Table S1.

### Generation of murine intestinal organoids

For the generation of intestinal organoids, mouse intestinal crypts were isolated from small intestines and colons, and organoids were generated using IntestiCult™ Organoid Growth Medium (Mouse, Stemcell Technologies, USA) following their standard protocol. Matrigel matrix (Corning) was used to culture these intestinal organoids.

### MNK-3 cell culture

MNK-3 cells were a kind gift from the **‘**late Dr. James Carlyle’ (University of Toronto, Canada)^47^. MNK-3 lines were cultured in Dulbecco’s modified Eagle’s medium-high glucose medium with 10% fetal bovine serum, 2 mM GlutaMAX, 1 mM sodium pyruvate, 55 μM 2-mercaptoethanol, 10 mM HEPES, 50 μg/ml gentamicin, 100U/ml penicillin, and 100 μg/ml streptomycin. MNK-3 cells were maintained in 10 ng/ml mouse IL-7 with or without 10 ng/ml mouse IL-15.

### Intestinal organoid and lamina propria cell/MNK-3 cell coculture

Intestinal organoids and lamina propria cells or MNK-3 cells were cocultured as described elsewhere^39,40^. Briefly, intestinal organoids, lamina propria lymphocytes and MNK-3 cells were cultured as described previously. After washing and counting, organoids and independently isolated LPLs pr MNK-3 cells were placed together onto a 24-well plate, resulting in 100 organoids and 1 × 10^5^ immune cells per experiment. Cell viability was assessed in all co-culture experiments using either trypan blue exclusion or flow cytometry–based live/dead staining. Viability remained high ( >90%) and comparable across all conditions.

### Flow cytometry of organoid and organoid-lamina propria cell/MNK-3 cell coculture

For flowcytometric analysis of organoids and organoid-lamina propria cell coculture, organoids were collected and washed with cold medium and centrifuged at 200 g for 5 min followed by resuspension in 1 mL of pre-warmed 1X TrypLE™ Express Enzyme (Thermofisher). Organoids were pipetted using a narrowed Pasteur pipette for 10 times and incubated for 5 min at 37 °C to make single cells. After incubation, the cells were pipetted 10 times and checked for single cells. Digestion was stopped by adding cold medium, and the digest was filtered through a 40 μm pore nylon cell strainer (Falcon) to remove doublets and further processed for flowcytometric analysis. For coculture, lamina propria single-cell suspensions or MNK-3 cells were also collected and stained for IL22^+^-ILC3 cells following flow cytometric methods as described previously. Single cells were stained with appropriate fluorochrome-labeled anti-mouse EpCAM, IL18, CD45.2, Lineage, Nkp46, RORγt, IL22 Ab, CD11c and F4/80. All intracellular staining was performed using True-Nuclear™ Transcription Factor Buffer Set (Biolegend, San Diego, CA, USA) as per the manufacturer’s instructions. The flowcytometric results were acquired with BD FACSCanto II. Data were analyzed using FlowJo software (Tree Star). A list of the antibodies used is provided in Table S1.

### Caspase activity assay

Caspase activity was evaluated using Caspase-Glo® 1 Inflammasome Assay (Promega) as per manufacture’s instructions.

### ChIP-qPCR

Colon organoids of C57BL/6 mice (n = 6) were cultured for 3 days in the presence of Veh (0.05% DMSO) or UroA (25 µM). The small intestinal (SI) tissue (1 cm) of C57BL/6 mice (n = 3) was cultured for 24 h in the presence of Veh (0.05% DMSO) or UroA (25 µM). The organoids and SI tissue were processed for ChIP assay using a Simple ChIP Enzymatic Chromatin IP kit (Cell Signaling Technology, 9003S) as per the manufacturer’s instructions. The anti-AHR antibody (Proteintech, 5.5 μg) was used to pull down AhR-binding DNA. The target DNA fragments were quantified by qPCR with primers listed in the key resources table.

The NM_133946 transcript of the mouse *Nlrp6* gene was selected to design primers for the ChIP-PCR assay. The *Nlrp6* gene -1000 bp promoter nucleotide sequence was selected from the Eukaryotic promoter database. Using JASPAR, we identified six putative AHR binding sites in the + strand of promoter region of the *Nlrp6* gene. List of the primers and sequences are provided in Table S1.

### Human intestinal specimens

For human samples, intestinal biopsies from the active inflamed region of individuals with inflammatory bowel disease (IBD) or controls were obtained following Institutional Ethical Review Board-approved protocols from the University of Louisville Hospital. Inflamed and non-inflamed regions were isolated by a trained pathologist. Written informed consent was obtained from all subjects. Details of human subjects are provided in Table S2.

### Flow cytometric analysis of human samples

Single-cell suspensions from intestinal tissues were obtained by incubating tissues for 30 min at 37 °C with shaking in stripping buffer (1 mM EDTA, 1 mM DTT and 5% FBS) to separate the epithelial layer. The supernatants containing the epithelial layer were kept separately, and the rest of the tissues were then mechanically dissociated with a sterile scalpel. The lamina propria fraction from the tissue was obtained by incubating the dissociated tissues for 1 h at 37 °C with shaking in 2 mg/ml collagenase D (Roche), 0.1 mg/ml DNase I (Sigma Aldrich) and 1 mg/ml of trypsin inhibitor (Gibco) digestion solution. Further, the remaining tissues were then filtered through a 70-μm cell strainer, and cells were further combined with the epithelial layer fraction to analyze as the whole tissue single cells. The single cell suspension was then filtered through a 70-μm cell strainer and cells were plated a 96-well plate (1 × 10^4^ cells/well) and incubated in DMEM with high glucose, supplemented with 10% FBS, 10 mM HEPES, 1 mM sodium pyruvate, non-essential amino acids, 80 μM 2-mercaptoethanol, 2 mM glutamine, 100 U/ml penicillin and 100 μg/ml streptomycin in the presence of recombinant human IL-1β (100 ng/ml) and human IL-23 (40 ng/ml) at 37 °C. Cells were incubated either in the presence of vehicle or UroA (25 μM) for 24 h. For intracellular cytokine trapping, cells were incubated for 4 h prior to the end point with 10 μg/ml brefeldin A. Cell viability was assessed in all experiments using either trypan blue exclusion or flow cytometry–based live/dead staining. Viability remained high ( > 90%) and comparable across all conditions. Single-cell suspensions were further stained with human-specific conjugated antibodies in PBS containing 2% FBS and 1 mM EDTA for extracellular and intracellular staining. Live CD45^+^LIN^−^CD127^+^CD117^+^NKp44^+^ were used to gate populations of human ILC3s. IL-22 expression was analyzed in ILC3 populations. All flow cytometry experiments were performed using a BD Canto flow cytometer and the FACS Diva software (BD Biosciences) and analyzed with FlowJo v.10 software (TreeStar).

### Statistical analysis

Statistical analysis was performed using GraphPad Prism software (GraphPad Software, San Diego, USA). Specific details of the statistical tests are provided in the figure legends. Data are represented as mean ± SEM from triplicate determinations, unless otherwise specified in the figure legends.

### Reporting summary

Further information on research design is available in the Nature Portfolio Reporting Summary linked to this article.

## Supplementary information

Supplementary Information

Transparent Peer Review file

Reporting Summary

## Source data

Source Data

## Data Availability

Data are available within the Article, Supplementary Information or Source Data file. Source data are provided with this paper.
