# Supplementary material for: Urolithin A activates aryl hydrocarbon receptor-NLRP6-mediated pathways in intestinal epithelial cells to modulate mucosal immunity and strengthen gut barrier integrity
Source: Nat Commun. 2026 Jun 23;17:5411. doi: 10.1038/s41467-026-73760-3 (PMC13291259; doi:10.1038/s41467-026-73760-3)
Supplement: Supplementary file 1 — Supplementary Information [file 41467_2026_73760_MOESM1_ESM.pdf]

Urolithin A activates aryl hydrocarbon receptor-NLRP6-mediated pathways in intestinal epithelial cells to modulate mucosal immunity and strengthen gut barrier integrity

Sweta Ghosh[1], Zachary M. Vanwinkle[1], Sobha Rani Bodduluri[1], Subir Kumar Juin[1], Mahendar Kadari[2], Ankita Singh[3], Gerald W Dryden[4], Matthew B. Lawrenz[2], Thirumala-Devi Kanneganti[5], Shesh N Rai[6], Misty Good[7], Pawan Kumar[3], Bodduluri Haribabu[1], Venkatakrishna Rao Jala[1*]

[1]Department of Microbiology and Immunology, UofL-Brown Cancer Center, Center for Microbiomics, Inflammation and Pathogenicity, University of Louisville, Louisville, KY, USA.

[2]Department of Microbiology and Immunology, Center for Predictive Medicine for Biodefense and Emerging Infectious Diseases, University of Louisville, Louisville, KY, USA.

[3]Department of Microbiology and Immunology, Stony Brook University, Stony Brook, NY, USA.

[4]Department of Medicine, University of Louisville, Louisville, KY, USA.

[5]Department of Immunology, St. Jude Children's Research Hospital, Memphis, TN, USA.

[6]Biostatistics and Informatics Shared Resource, University of Cincinnati Cancer Center, Cancer Data Science Center, University of Cincinnati College of Medicine, Department of Biostatistics, Health Informatics and Data Sciences, University of Cincinnati College of Medicine, Cincinnati OH, USA.

[7]Division of Neonatal-Perinatal Medicine, Department of Pediatrics, University of North Carolina at Chapel Hill, Chapel Hill, NC, 27599, USA

***Corresponding Author**

Venkatakrishna Rao JALA, Ph. D

Email: jvrao001@louisville.edu

Supplementary Figures

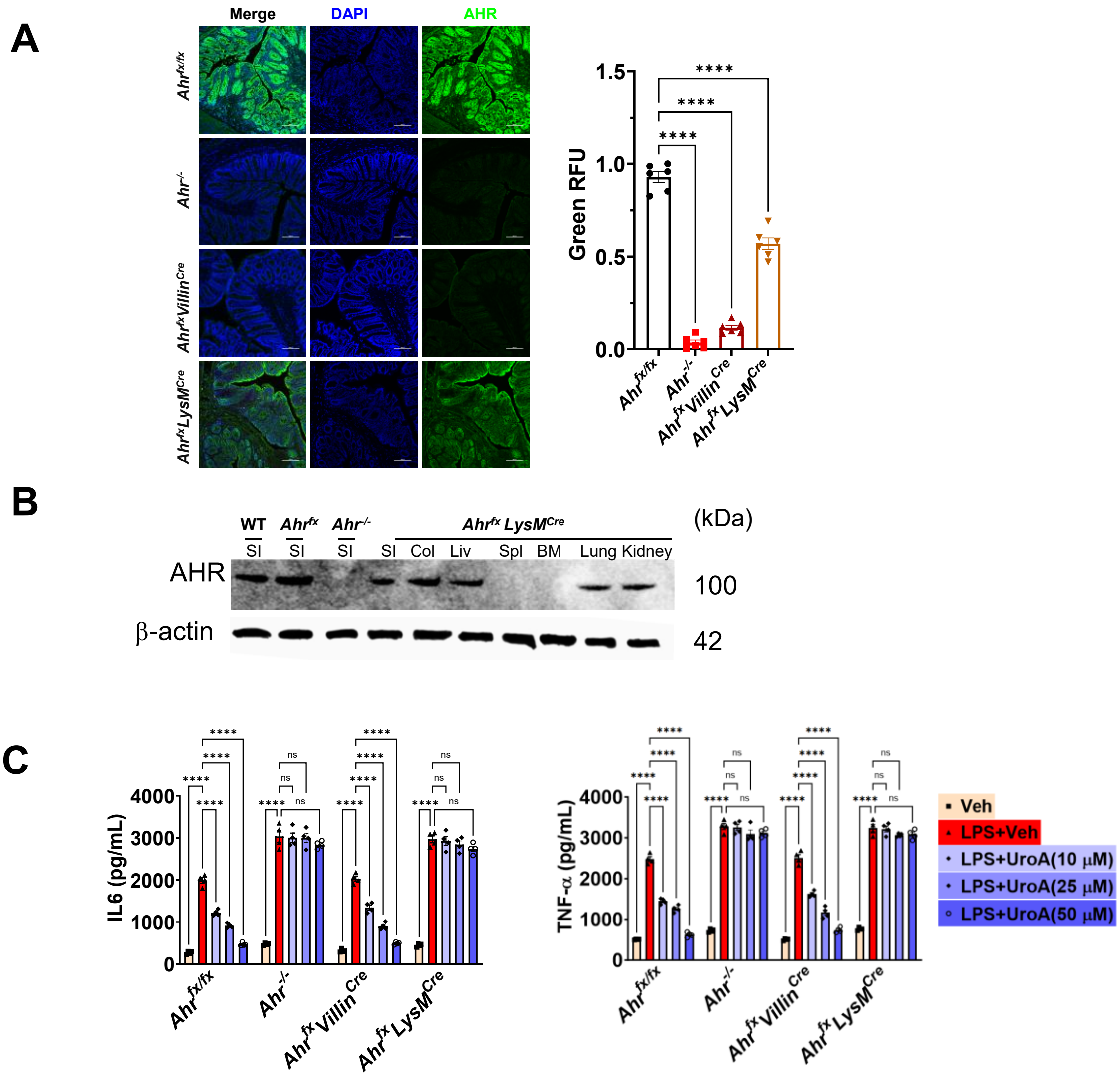

Figure S1. Characterization of cell-specific *AhR* deleted transgenic mice.
(A) Confocal images of colon sections stained with anti-AHR antibody followed by secondary antibody tagged with Alexa 488. Nucleus stained with DAPI. The fluorescence images were captured using Nikon A1R confocal microscope. The scale bar indicates 100 µm. Data suggest deletion of AHR in intestinal epithelial cells in *Ahr*^{fx}-*Villin*^{Cre} mice, but not in *Ahr*^{fx}-*LysM*^{Cre} mice. The fluorescence intensity ($n = \sim 20$ cell) was measured. Statistics were performed by One Way ANOVA. ****p<0.0001. **(B)** Western blot analysis of AHR expression from the different tissues of WT, *Ahr*$^{\text{fx/fx}}$, *Ahr*$^{-/-}$ and *Ahr*^{fx}-*LysM*^{Cre} mice. SI: small intestine; Col: colon; Liv: liver; Spl: Spleen; BM: bone marrow cells. **(C)** Bone marrow derived macrophages (BMDM) were prepared from *Ahr*$^{\text{fx/fx}}$, *Ahr*$^{-/-}$, *Ahr*^{fx}-*Villin*^{Cre} and *Ahr*^{fx}-*LysM*^{Cre} mice. BMDMs were treated with LPS (50 ng/ml) in the presence of vehicle (Veh) or UroA (10, 25 and 50 µM) for 6 h. The cytokines, IL-6 and TNF-α levels were measured by standard ELISA. The data is representative of two independent experiments with quadruplets. Statistics were performed using two-way ANOVA. Error bars, ±SEM; ns: Not significant, **p<0.01; ***p<0.001, ****p<0.0001. The actual p-values are provided in source data file. All experiments were repeated at least three times using biologically independent replicates, yielding similar results. Source data are provided as a Source data file.

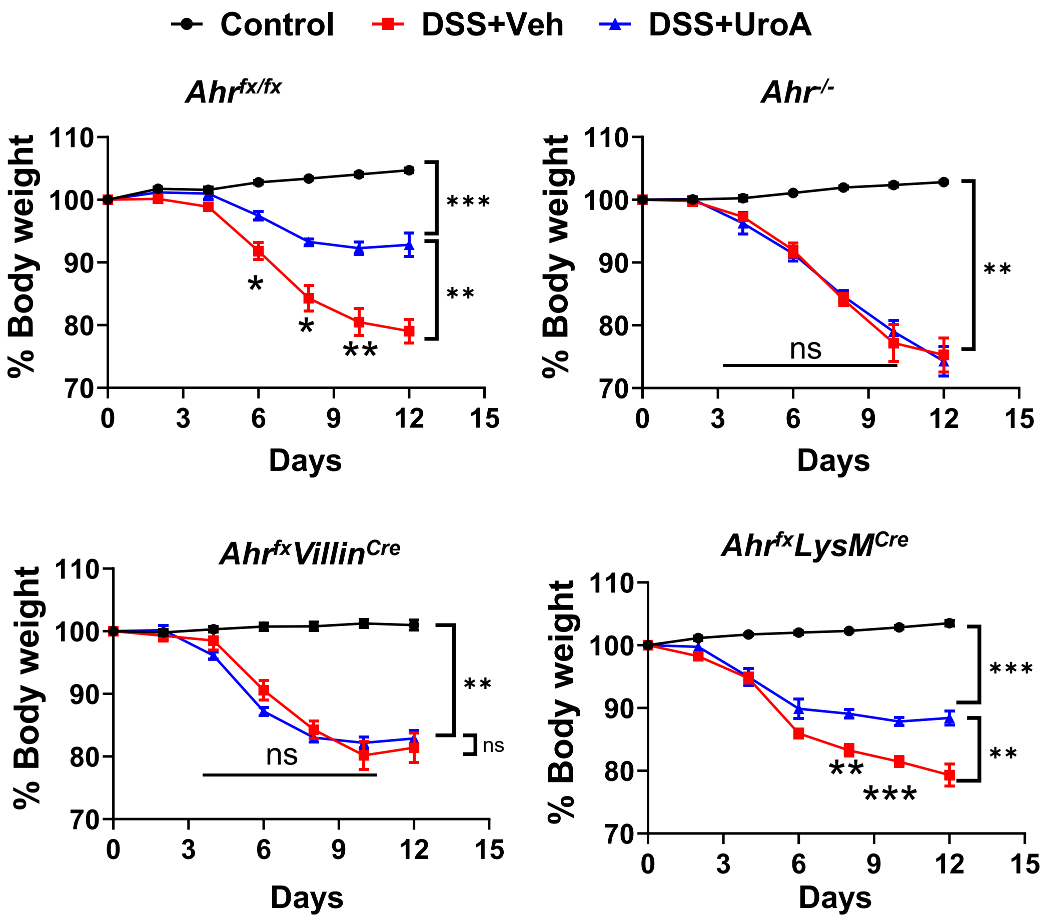

Figure S2. Extended analysis of Figure 1. Intestinal AHR is required for UroA mediated protection against colitis. Percent body weight loss of indicated mouse genotypes that are subjected to acute DSS-induced colitis as described in Figure 1 and methods. Statistics were performed 2 WAY ANOVA compared DSS+Veh vs DSS+UroA. Error bars, ±SEM; ns: Not significant, **p<0.01; ***p<0.0001. The actual p-values are provided in source data file. All experiments were repeated at least three times using biologically independent replicates, yielding similar results. Source data are provided as a Source data file.

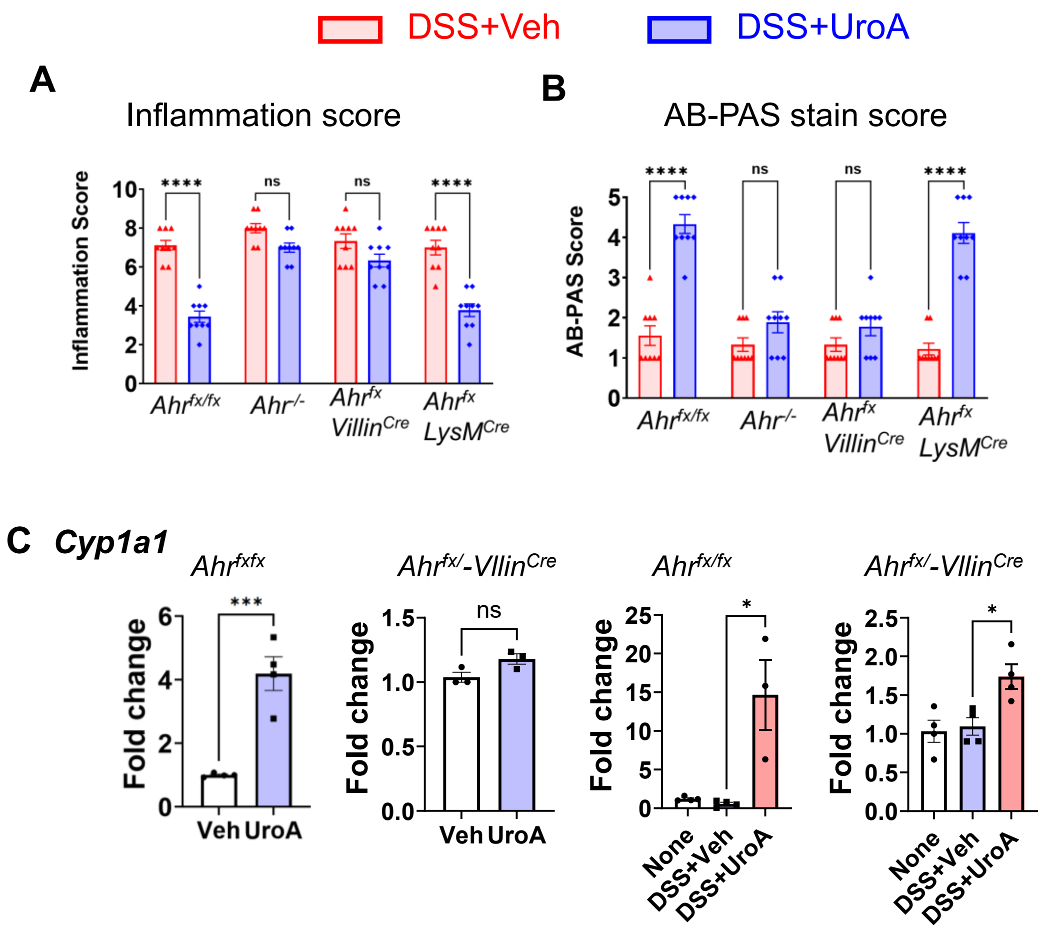

Figure S3. Extended analysis of Figure 1. Intestinal AHR is required for UroA mediated protection against colitis. **(A)** H&E sections of colons of indicated genotype mice that were subjected to DSS-induced colitis model were scored for inflammation as described in methods. **(B)** Colon sections were stained with AB-PAS and scored as described in methods. **(C)** $Ahr^{fx/fx}$ and Ahr^{fx}-$Villin^{Cre}$ mice were treated with Vehicle or UroA (20 mg/kg) alternate day for 12 days. The total RNA from the colons of these mice were isolated and measured the mRNA levels of *Cyp1A1*. Similarly, these mice subjected acute DSS-induced colitis and determined the expression of *Cyp1a1* by SyBR RT-PCR method. Fold changes were calculated as described in methods. Statistics were performed using one-way ANOVA. Error bars, ±SEM; ns: Not significant, *p<0.05; ****p<0.0001. The actual p-values are provided in source data file. All experiments were repeated at least three times using biologically independent replicates, yielding similar results. Source data are provided as a Source data file.

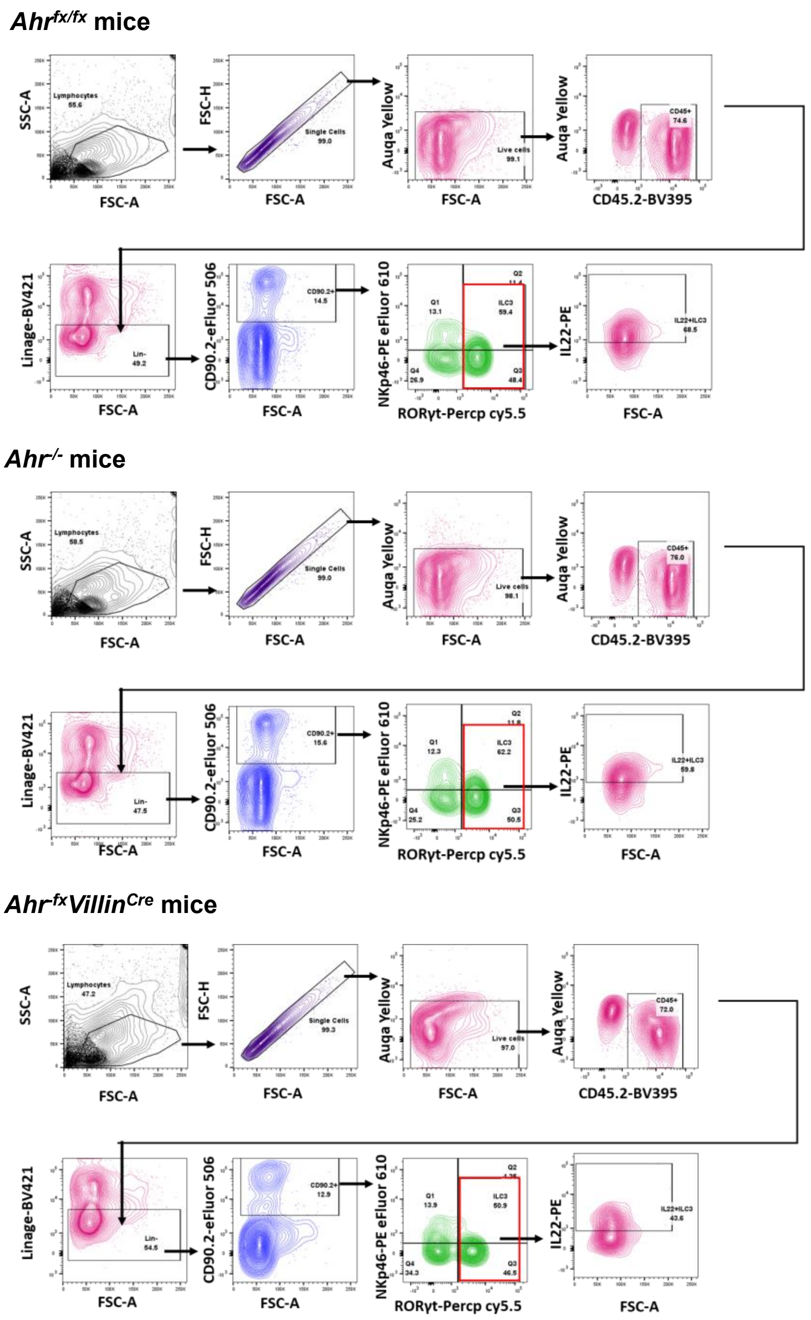

Figure S4. Gating strategy for IL-22$^+$ILC3 cells from lamina propria (LP). The representative flow cytometry gating strategy for ILC3-IL-22 of colon lamina propria of *Ahr$^{fx/fx}$*, *Ahr$^{-/-}$* *Ahr^{fx}-Villin^{Cre}* mice. Lamina propria single-cell suspension lymphocytes were gated with the forward scatter/side scatter (FSC/SSC) strategy. Single and CD45.2$^+$ live cells were only considered for analysis. Negative lineage cells (CD3$^-$, CD19$^-$, Ly-6G /Ly-6C$^-$, F4/80$^-$, CD5$^-$, TCR β$^-$, TCR γδ$^-$) were gated to analyze CD90.2$^+$ innate lymphocyte cells. Expression of RORγt and Nkp46 served to distinguish the different populations of ILCs. Q2 (RORγt$^+$ Nkp46$^+$) corresponded to the ILC3 NCR$^+$ population and Q3 (RORγt$^+$ Nkp46$^-$) included ILC3 NCR$^-$ and Lti cells. Together Q2 and Q3 are total ILC3s. The expression of IL-22-PE was analyzed in all ILC3 populations.

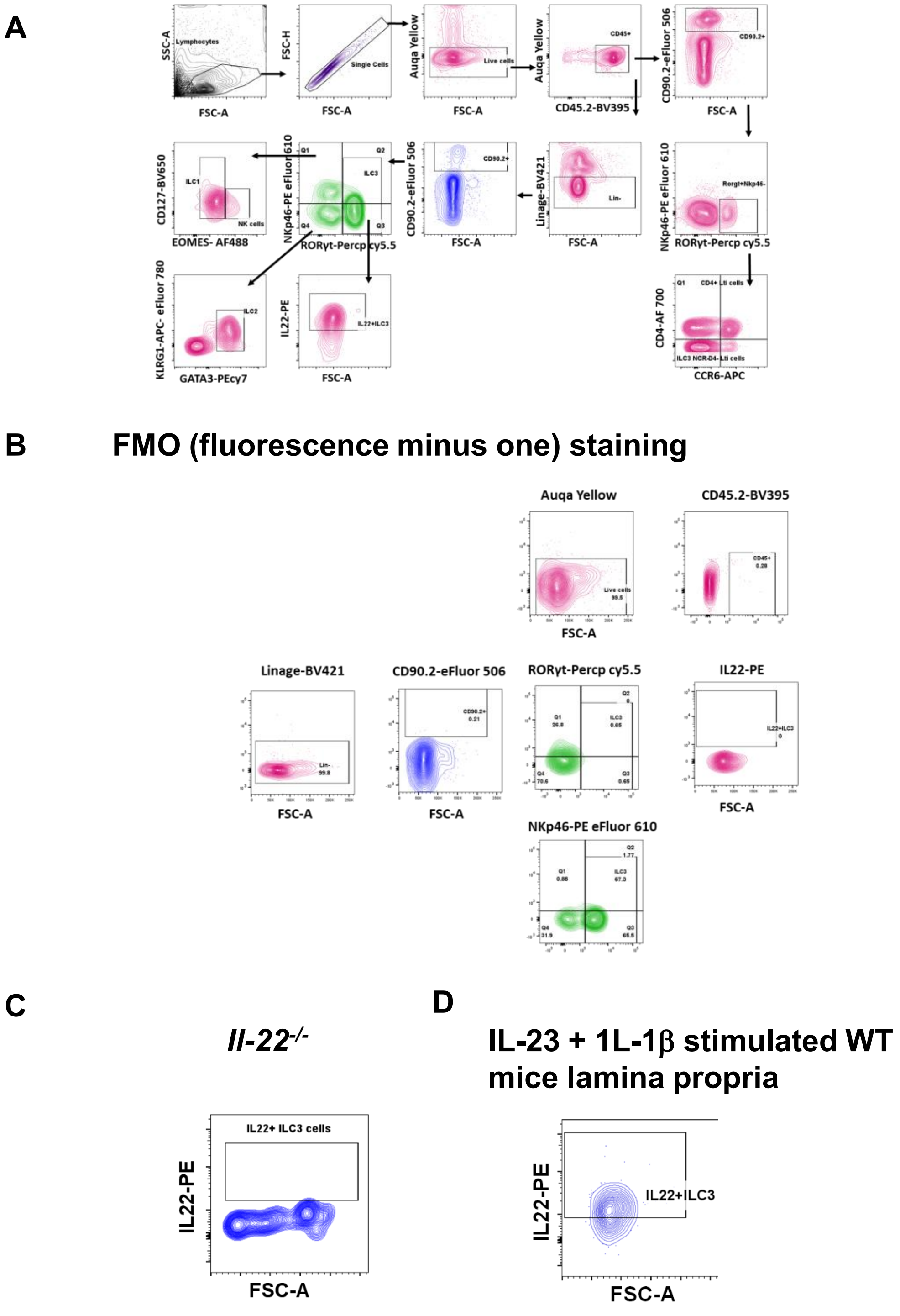

Figure S5: Gating strategies for different ILCs-IL-22 levels. A. Lamina propria single-cell suspension lymphocytes were gated with the forward scatter/side scatter (FSC/SSC) strategy. Single and CD45.2^{+} live cells were only considered for analysis. Negative lineage cells were gated to analyze CD90.2^{+} innate lymphocyte cells. Expression of RORgt and Nkp46 served to distinguish the different populations of ILCs. Q2 corresponded to the ILC3 NCR^{+} population Q3

included ILC3 NCR$^-$ and Lti cells. Together Q2 and Q3 are total ILC3s. The expression of IL-22-PE was analyzed in all ILC3 populations. Q1 included the NK and ILC1 population, which was differentiated with CD127 and Eomes. ILC2 population in Q4 was differentiated with expression of KLRG1 and GATA3. The Lti subpopulation, gated on CD45$^+$CD90.2$^+$RORgt$^+$Nkp46$^-$, expresses CCR6, and is divided into CD4$^+$ Lti and CD4- Lti cells. **B.** Fluorescence minus one (FOMO) gating strategy scatter data plots are shown as controls for gating. **C.** Lamina propria prepared from *Il-22*$^{-/-}$ mice were used as negative control. **D.** Lamina propria cells were isolated from wild type mice and treated with IL-23+IL-β to stimulate IL-22. These cells were used as positive control for IL-22 staining.

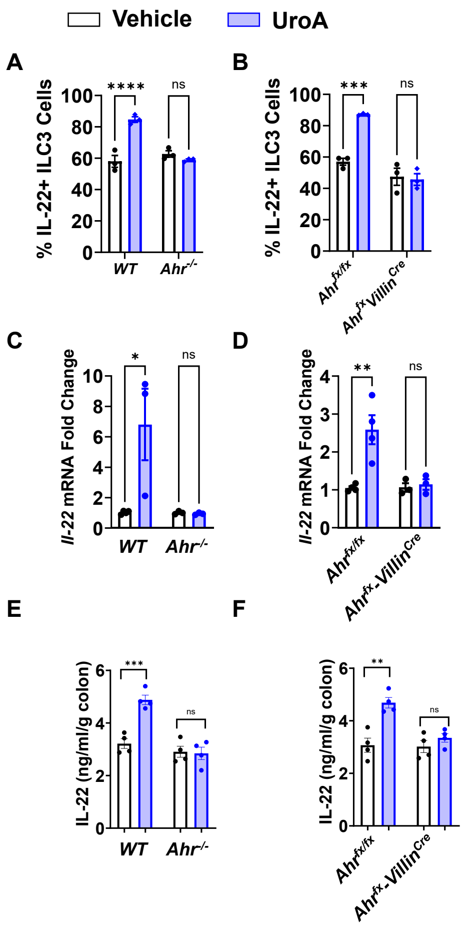

Figure S6. Wild type (WT), *Ahr*$^{-/-}$, *Ahr*$^{fx/fx}$ and *Ahr*^{fx}-*Villin*^{Cre} mice (6–8-week age old; total n=3-4 consisting of males (n=1-2) and females (n=2) mice per group) were treated with either Vehicle (1% CMC+0.1% Tween-80) or UroA (20 mg/kg) orally every 48 h starting from day 0. **(A)** Single cell suspensions from lamina propria (LP) were prepared from colon of *Ahr*$^{fx/fx}$, *Ahr*$^{-/-}$ and *Ahr*^{fx}-*Villin*^{Cre} mice. The IL-22^{+} ILC3s were measured using standard flow cytometry methods as described in methods and the gating strategy is shown in Figure S4. Percentage and total number of IL22^{+}ILC3 cells are shown. Statistics were performed two-Way ANOVA test. Error bars, ±SEM; ns: Not significant, **** p<0.0001. **(B)** Flow cytometry diagram of IL-22 levels are shown for indicated mice and treatments. **(C)** The fold changes of *Il-22* mRNA levels in the colons of WT and *Ahr*$^{-/-}$ mice; **(D)** *Ahr*$^{fx/fx}$ and *Ahr*^{fx}-*Villin*^{Cre} mice were determined by SYBR green RT-PCR method. (**E-F**) Protein levels of IL-22 were determined in colon homogenates using standard ELISA. Statistics were performed using one-way ANOVA. Error bars, ±SEM; ns: Not significant, *p<0.05, **p<0.01, ***p<0.001, ****p<0.0001. The actual p-values are provided in source data file. All experiments were repeated at least three times using biologically independent replicates, yielding similar results. Source data are provided as a Source data file.

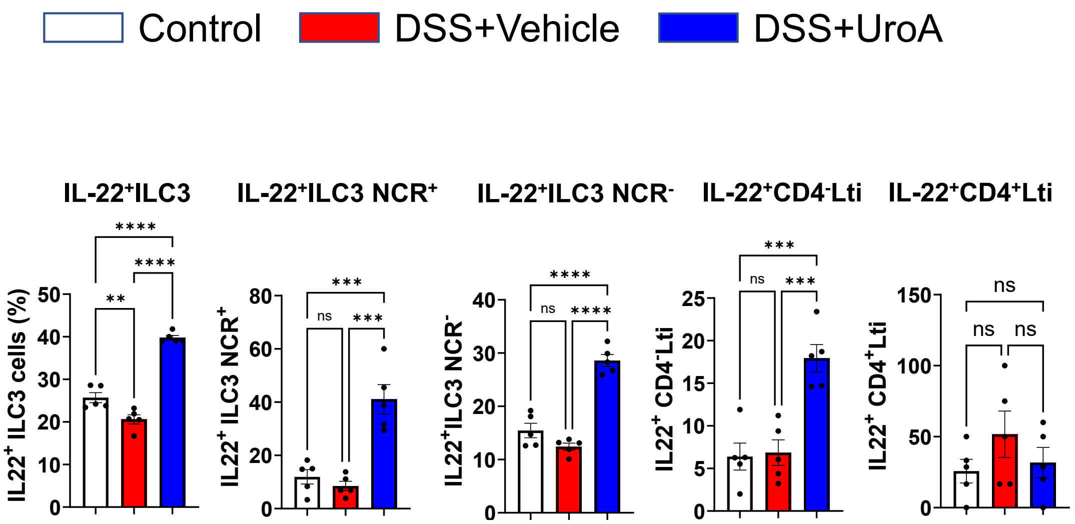

Figure S7. Single cell suspensions from lamina propria (LP) were prepared from colon. The IL-22^{+} ILC3s were measured using standard flow cytometry methods as described in methods. The gating strategy for different ILC3 populations is described in Figure S5A. Percentage of IL22^{+}ILC3 cells are shown. Statistics were performed using two-Way ANOVA test. Error bars, ±SEM. ns: Not significant, *p<0.05, **p<0.01, ***p<0.001, ****p<0.0001. The actual p-values are provided in source data file. All experiments were repeated at least three times using biologically independent replicates, yielding similar results. Source data are provided as a Source data file.

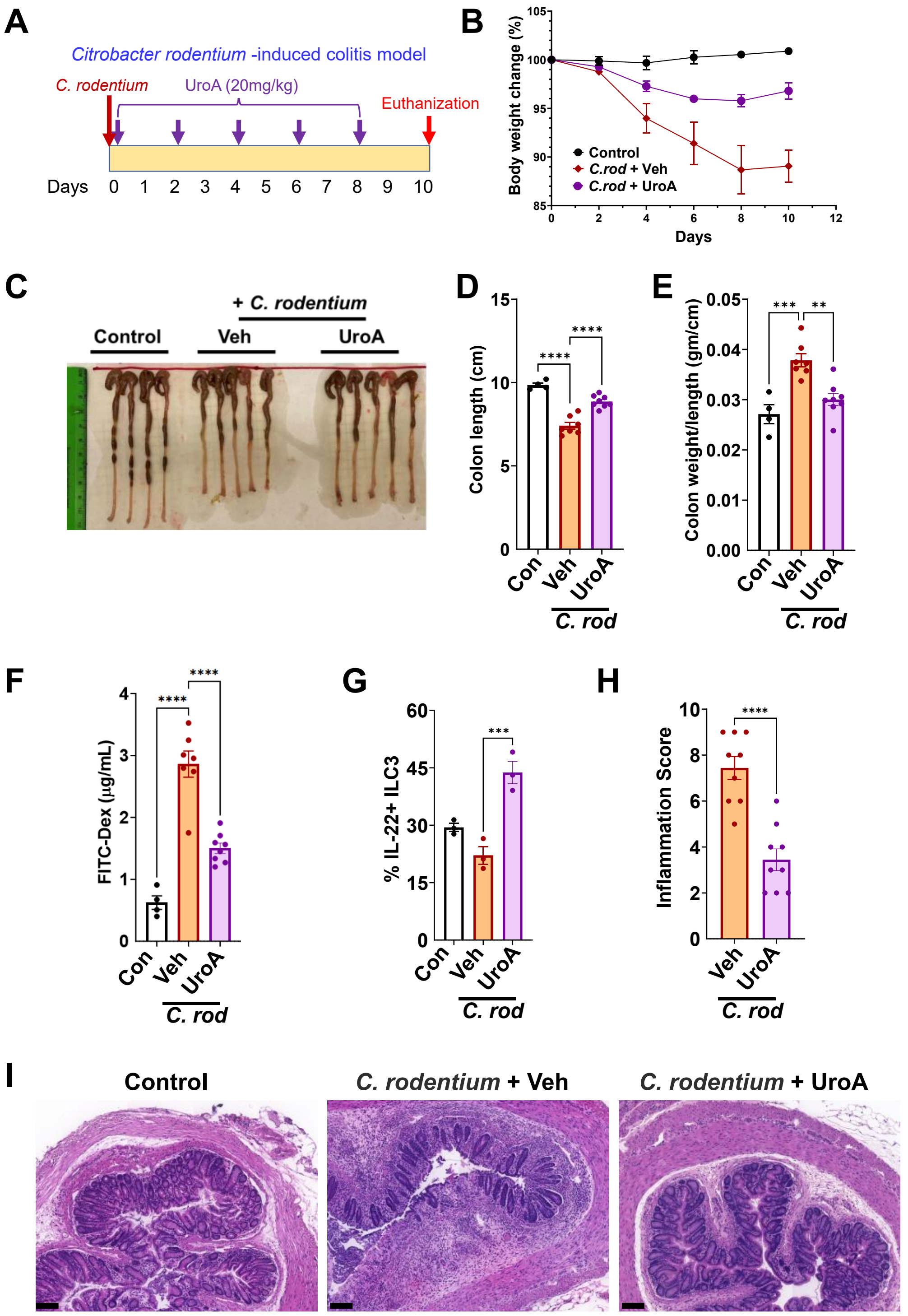

Figure S8. UroA treatment induced IL-22 in *Citrobacter rodentium*-induced colitis model and attenuated colitis.

(A) Schematic diagram of *C. rodentium*-induced colitis. C57BL/6J mice (8 week old age, n=8 per group consisting of male (n=4) and female (n=4) mice per group) were subjected to *C.rodentium*-induced colitis. Mice were treated with either Vehicle or UroA (20 mg/kg) orally every 48 h post

C. rodentium infection. Mice were euthanized at day 12. **(B)** Percent body weight loss of indicated mouse groups. **(C-E)** Gross images of representative colons (**C**) are shown. Colon lengths **(D)** and ratio of Colon weight/length **(E)** are shown. **(F)** Intestinal permeability of the mice was measured using FITC-dextran permeability assay. **(G)** Lamina propria cells were analyzed for IL-22^{+} ILC3s using standard flow cytometric procedures as described above. Percentage of IL22^{+}ILC3 are shown. **(H)** Inflammation scores of colon hematoxylin and eosin (H&E) stained sections. **(I)** Representative microphotographs of H&E stained sections of colons are shown. Scale bar indicates 100 μm. Statistics were performed using one-way ANOVA. Error bars, ±SEM; ns: Not significant, **p<0.01; ***p<0.001, ****p<0.0001. The actual p-values are provided in source data file. All experiments were repeated at least three times using biologically independent replicates, yielding similar results. Source data are provided as a Source data file.

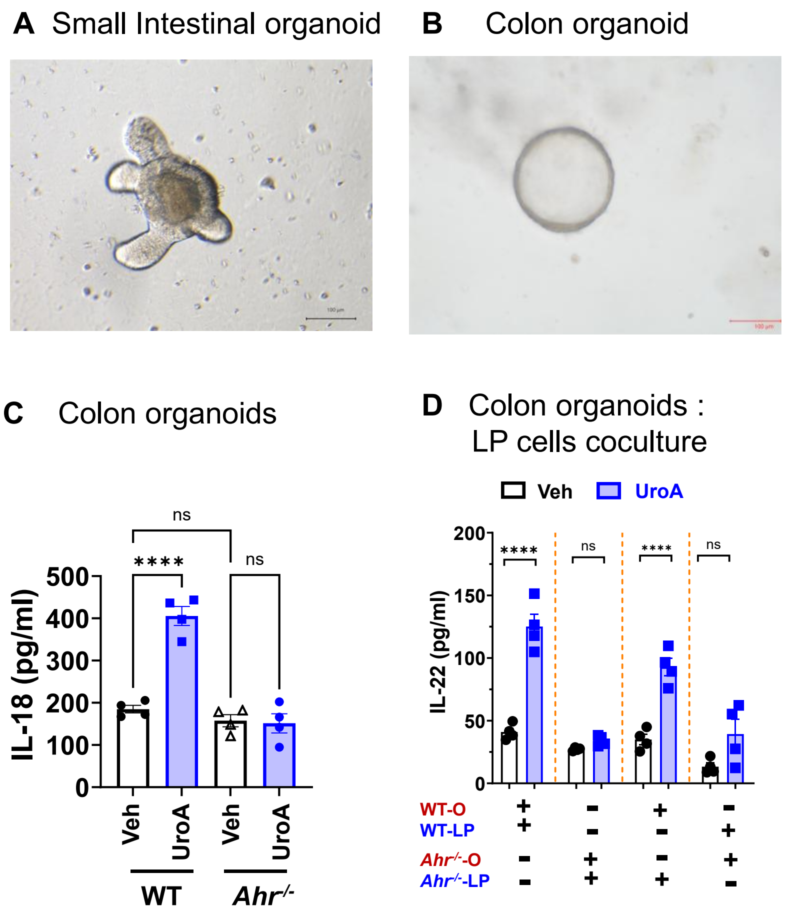

Figure S9. UroA treatment upregulated IL-18 and IL-22 in colon organoids and lamina propria cells respectively.

(A) Representative image of small intestinal organoid on day 7. Scale bar indicates 100 µm. **(B)** Representative image of colon organoid on day 7. Scale bar indicates 100 µm. **(C)** WT and *Ahr*$^{-/-}$ colon organoids (n= 100 organoids) were treated with Vehicle (0.05% DMSO) or UroA (25 µM) for 72 h. The levels of IL-18 were measured in the supernatants using ELISA methods. **(D)** Colon organoids (n= 100 organoids from WT or *Ahr*$^{-/-}$ mice) were cultured with or without LP cells ($1x10^5$ cells from WT and *Ahr*$^{-/-}$ mice) for 3 days in presence of Veh (0.05% DMSO) or UroA (25 µM). IL-22 levels were determined in supernatants of indicated co-cultures using standard ELISA in a separate set of experiment. Statistics were performed using two-way ANOVA. Error bars, ±SEM; ns: Not significant, *p<0.05, **p<0.01, ***p<0.001, ****p<0.0001. The actual p-values are provided in source data file. All experiments were repeated at least three times using biologically independent replicates, yielding similar results. Source data are provided as a Source data file.

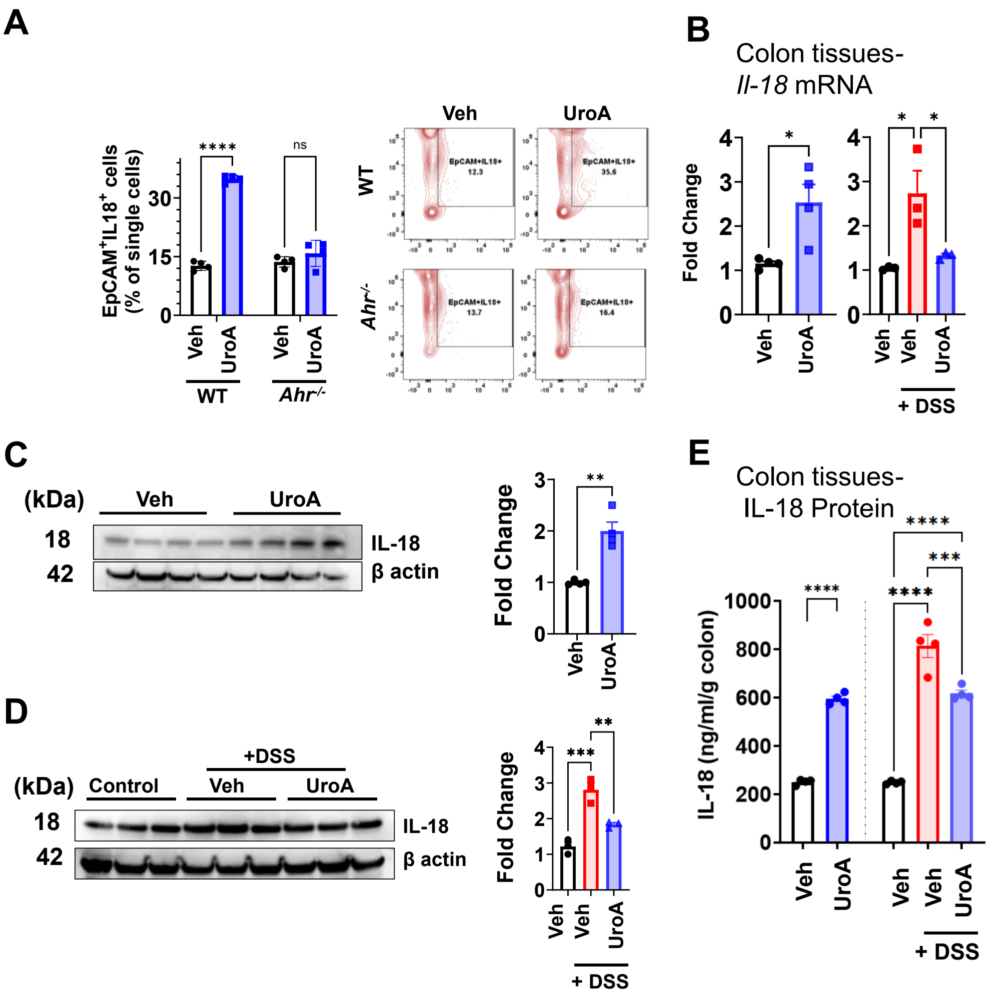

Figure S10. UroA-induced IL-22 from ILC3 is dependent on IEC-AHR-IL-18 axis.

(A) Intestinal organoids were prepared from WT and *Ahr*$^{-/-}$ mice were treated with vehicle (0.05% DMSO) or UroA (25 µM) for 72 h. Single cell suspensions were prepared from organoids and stained with EpCAM-APC and IL18-FITC antibodies using standard flow cytometry methods. EpCAM^{+}-IL-18^{+} cells were determined. **(B)** WT mice (C57BL/6, n=5-7, 6–8-week age old) were either on normal water (control group) or subjected to 2.5% DSS in drinking water for 7 days followed by 5 days of regular water. Both control and DSS-subjected mice were treated with either vehicle (1% CMC+0.1% Tween 80) or UroA (20 mg/kg) orally every 48 h of intervals. E. The fold changes in mRNA levels of IL-18 in the colons were determined by SYBR green RT-PCR method. Expression patterns of IL-18 protein in the colons of control group **(C)** and colitis mice **(D)** was measured by Western blot and quantified by using Image J software. **(E)** IL-22 protein levels were determined in colon homogenates using standard ELISA. Statistics were performed using either one-way ANOVA or unpaired t-test. Error bars, ±SEM; ns: Not significant, *p<0.05; **p<0.01; ***p<0.001, ****p<0.0001. The actual p-values are provided in source data file. All experiments were repeated at least three times using biologically independent replicates, yielding similar results. Source data are provided as a Source data file.

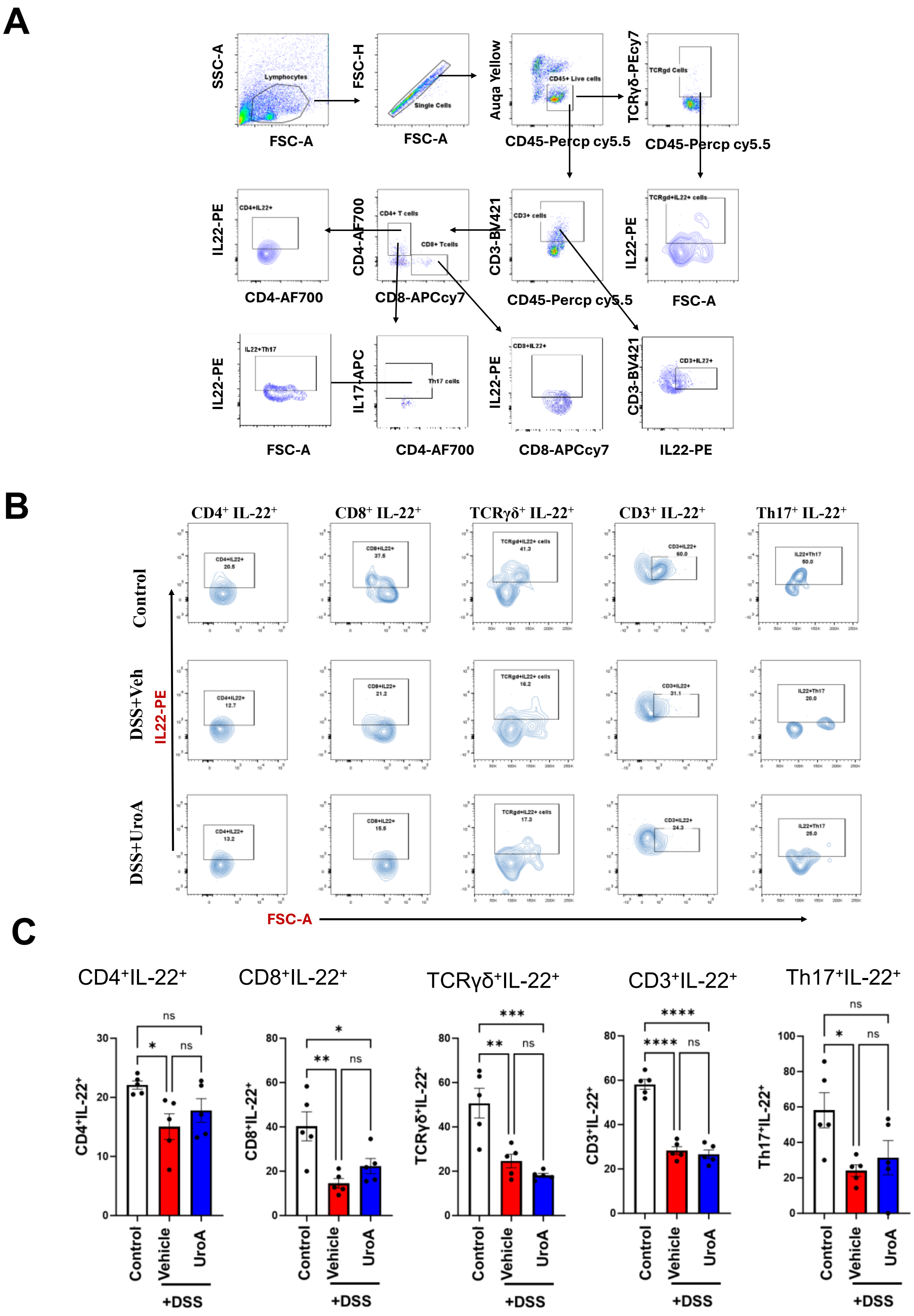

Figure S11. Analysis of IL-22 secretion from different cell types *in vivo*. Lamina propria single-cell suspension lymphocytes were gated with the forward scatter/side scatter (FSC/SSC) strategy.

The IL-22$^+$ ILC3s were measured using standard flow cytometry methods as described in methods. Single and CD45.2-APCcy7$^+$ live cells were only considered for the analysis. CD3$^+$ CD45.2-APCcy7$^+$ and TCRγδ$^+$ CD45.2-APCcy7$^+$ cells were analyzed for expression of IL-22-PE. CD3 cells were further gated for CD4$^+$ and CD8$^+$ T cells. CD4$^+$ cells were gated for IL17$^+$ Th17 cells. The expression of IL-22-PE was analyzed in CD4$^+$, CD8$^+$ and Th17 cell populations. (**A**) Gating Strategy (**B**) Representative contour plots of flow cytometry analysis of IL-22 levels in different cell types. **(C)** Percentage of different IL22$^+$ cells are shown. Statistics were performed One-Way ANOVA test. Error bars, ±SEM. ns: Not significant, *p<0.05, **p<0.01, ***p<0.001, ****p<0.0001. The actual p-values are provided in source data file. All experiments were repeated at least three times using biologically independent replicates, yielding similar results. Source data are provided as a Source data file.

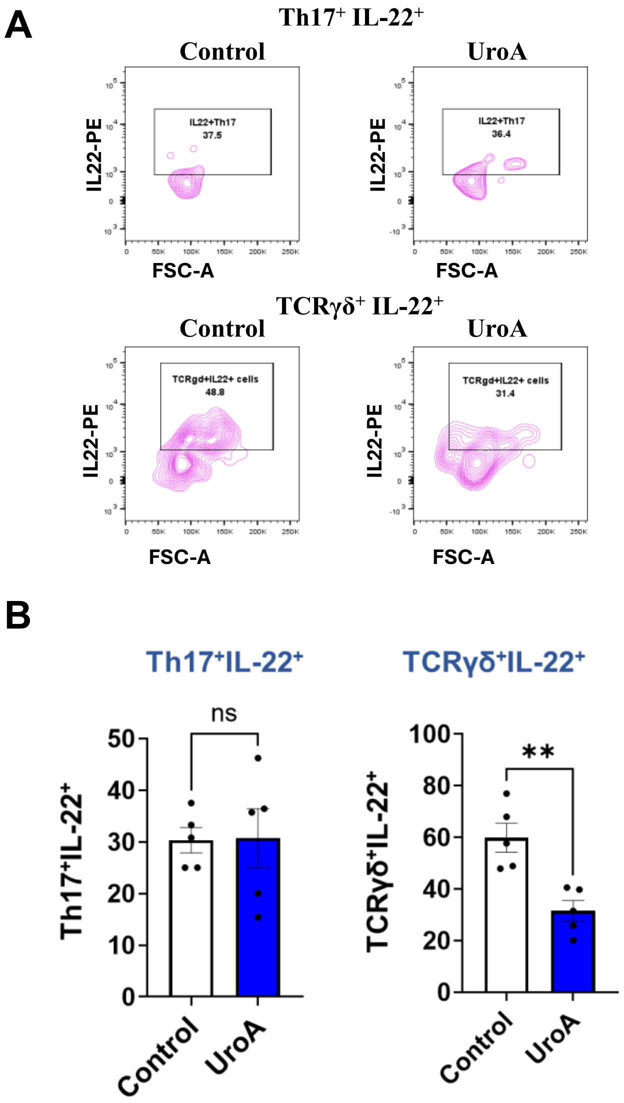

Figure S12. Analysis of IL-22 secretion from different cell types (Th17$^+$ and TCRγδ$^+$ cells) in organoid:lamina propria (LP) coculture. Colon organoids (n= 100 organoids from WT$^-$ mice) were cultured with LP cells (1x10^5 cells from WT mice) for 3 days in presence of Veh (0.05% DMSO) as control or UroA (25 μM). IL-22 secretion from indicated cell types were determined using standard flow cytometry methods as described in methods and Figure S11. (**A**) Representative contour plots of flow cytometry analysis. **(B**) Quantification of IL-22$^+$ cells from indicated cell types. Statistics were performed using unpaired t-test. Error bars, ±SEM. ns: Not significant, **p<0.01. The actual p-values are provided in source data file. All experiments were repeated at least three times using biologically independent replicates, yielding similar results. Source data are provided as a Source data file.

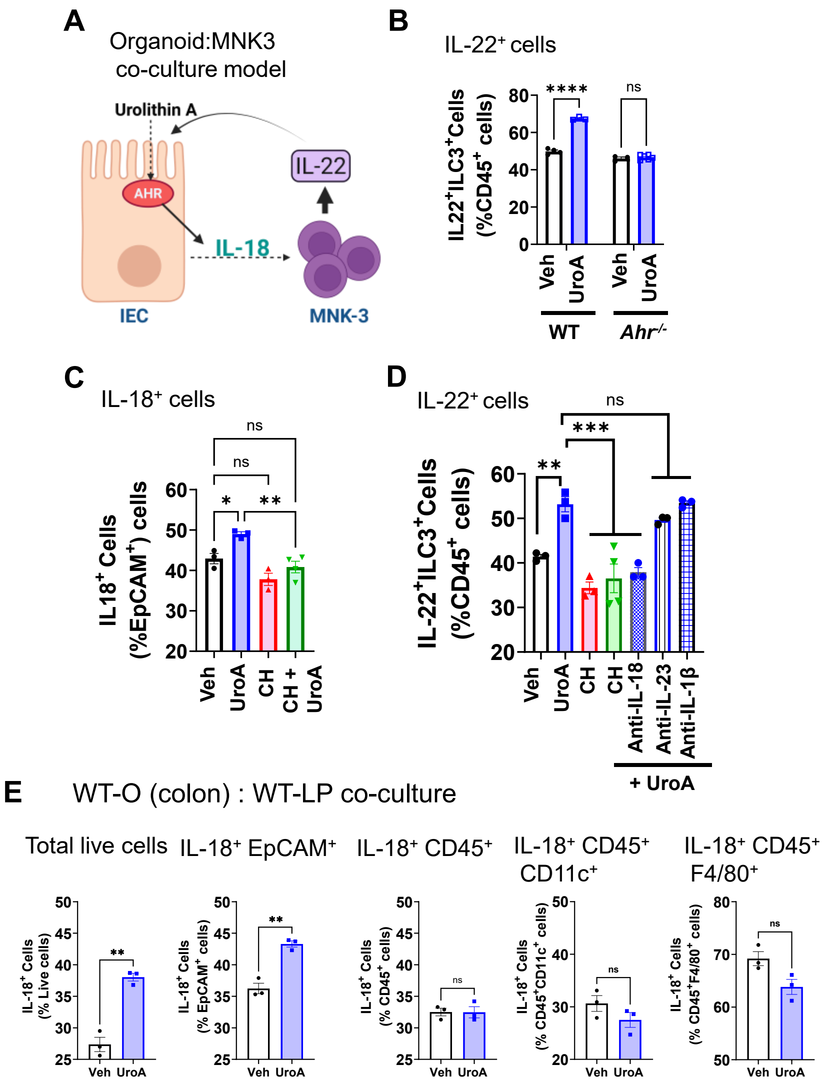

Figure S13. UroA induces IL-22 from ILC3 in an IEC-IL-18 dependent manner and IL-18 production independent of immune cells.

(A) Scheme representing IEC and MNK3 (ILC3 cell line) cells coculture-experiment to evaluate the role of AHR-IL18 axis to induce IL-22 from ILC3. Created in BioRender. Ghosh, S. (2026) https://BioRender.com/e6o5nnn **(B)** WT and *Ahr*$^{-/-}$ intestinal organoids (n= 100 organoids) were co-cultured with MNK3 cells and treated with vehicle (0.05% DMSO) or UroA (25 μM) for 24h. The IL-22 producing MCK3 were analyzed using flow cytometry as described in Figure 2A. Statistics were performed using two-way ANOVA. Error bars, ±SEM; ns: Not significant, ****p<0.0001. **(C)** WT-O:MNK3 cells coculture were treated with vehicle or UroA (25 μM) in presence or absence of CH223191 (10 μM). Single cells were prepared and stained for EpCAM^{+} IL-18^{+} cells and analyzed by standard flow cytometry methods. Statistics were performed using one-way ANOVA. Error bars, ±SEM; ns: Not significant, ****p<0.0001. **(D)** WT-O:MNK3 cells coculture were treated with UroA (25 μM) in presence or absence of CH223191 (10 μM) or anti-

IL18 Ab, anti-IL23 Ab, anti-IL1β Ab (20 ng/ml) for 24 h. The single cells were prepared from stained for IL-22^{+} ILC3 using standard flow cytometry methods. Statistics were performed using one-way ANOVA. Error bars, ±SEM; ns: not significant, *p<0.05, **p<0.01, ***p<0.001. **(E)** WT colon organoids and WT LP cells were co-cultured and IL-18 expression was determined in IEC (EpCAM^{+}) and immune cells (CD45, CD11c, F4/80 positive cells) using standard flow cytometry methods. Cells were gated as described in Supplementary Fig. 14. Statistics were performed using unpaired t-test. Error bars, ±SEM; ns: Not significant, **p<0.01. The actual p-values for all the comparisons are provided in source data file.

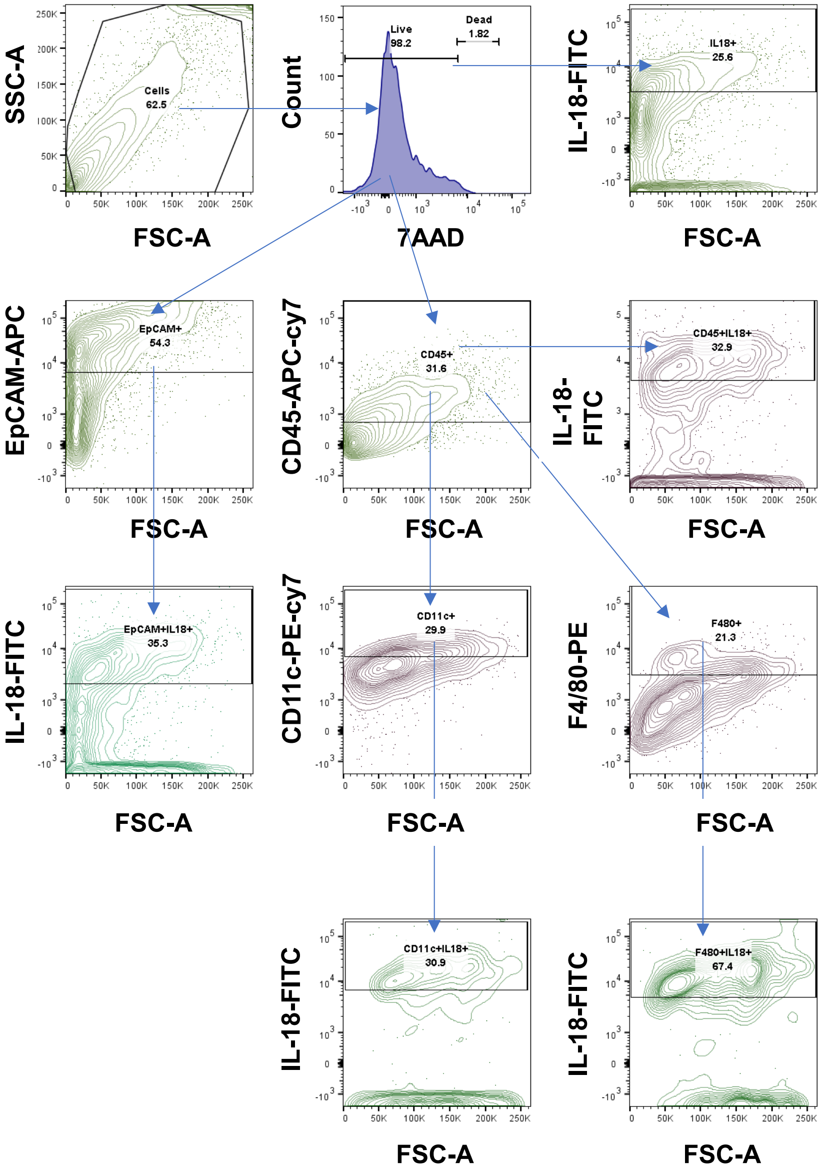

Figure S14. Gating strategy for determination of IL-18 production from epithelial and immune cells. WT organoid and LP single-cell suspension were gated with the forward scatter/side scatter (FSC/SSC) method. Live ($7AAD^{+}$) cells were gated for total IL-18^{+} on $EpCAM^{+}$ and $CD45^{+}$ cells. EpCAM-APC^{+} and CD45-$APCcy7^{+}$ cells were further gated for $IL18^{+}$ cells. Single CD45-$APCcy7^{+}$ live cells were gated to CD11c-$PEcy7^{+}$ and F4/80-PE^{+} cells. Expression of IL18-FITC were determined from those populations.

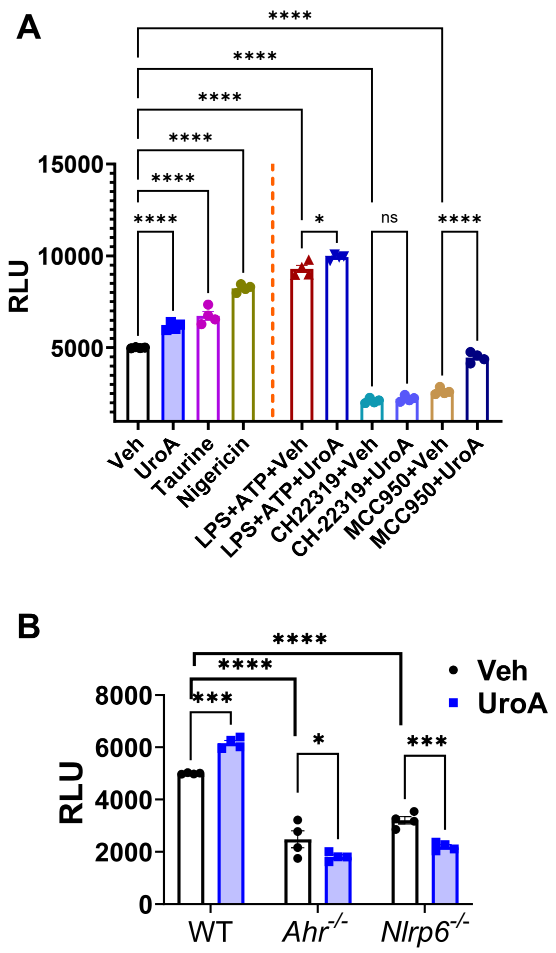

Figure S15. UroA activates and induces NLRP6 in AHR-dependent manner. (A) WT intestinal organoids treated with vehicle (0.05% DMSO) or UroA (25 µM) or taurine (25 µM), nigercin (10 µM) or LPS (10 ng/ml) + ATP (5 mM), CH-22319 (10 µM), MCC950 (1 µM) in the presence/absence of UroA for 24 h. Caspase 1 activity was measured using Caspase-Glo1 kit following manufacture's instruction. Statistics were performed using one-way ANOVA. Error bars, ±SEM; ns: Not significant,*p<0.05, ****p<0.0001 **(B)** Intestinal organoids from WT, *Ahr*$^{-/-}$ and *Nlrp6*$^{-/-}$ mice were generated and treated with vehicle (0.05%) or UroA (25 µM) for 24 h and measured caspase 1 activity. Statistics were performed using two-way ANOVA. Error bars, ±SEM; *p<0.05, ***p<0.001, ****p<0.0001. The actual p-values for all the comparisons are provided in source data file. All experiments were repeated at least three times using biologically independent replicates, yielding similar results. Source data are provided as a Source data file.

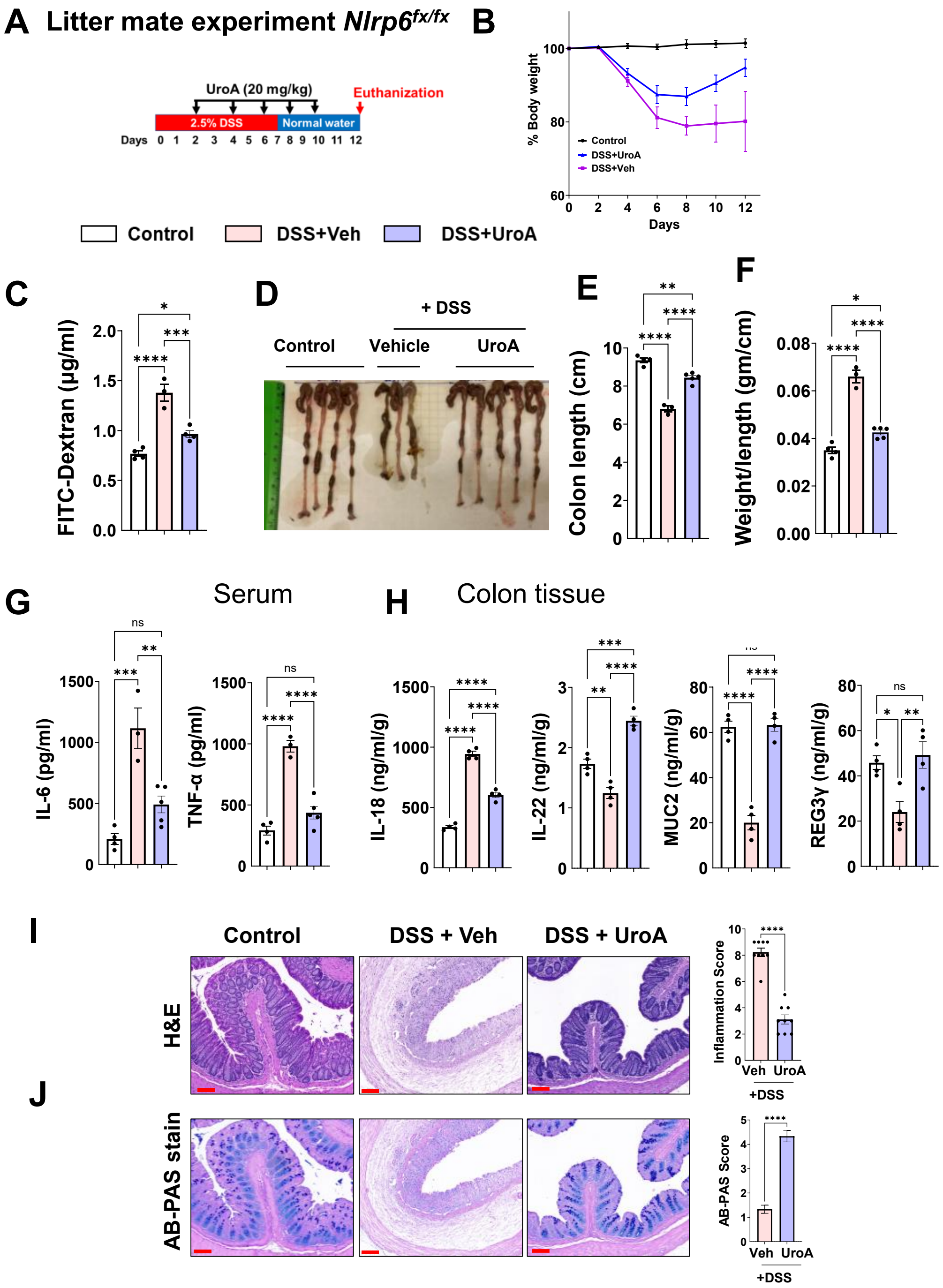

Figure S16. Littermate (*Nlrp6*$^{fx/fx}$) control experiment for *Nlrp6*^{fx}*Villin*^{Cre} mice (Figure 5). Intestinal epithelial cell NLRP6 is required for UroA-mediated protective activities against DSS-induced colitis. **(A)** *Nlrp6*$^{fx/fx}$ mice (6–8-week age old, n=5-7 consisting of males (n=3-4) and females (n=2-3) mice per group) were either on normal water (control group) or subjected to 2.5% DSS in drinking water for 7 days followed by 5 days of regular water. Both control and DSS-subjected mice were treated with either vehicle (1% CMC+0.1% Tween 80) or UroA (20 mg/kg)

orally every 48 h of intervals. **(B)** Percent body weight loss. **(C)** Intestinal permeability was assessed by using FITC-dextran intestinal permeability assay as described in methods. **(D)** Gross images of colons are represented. **(E)** Colon lengths were measured. **(F)** Ratio of Colon weight/length are shown. **(G)** Serum IL-6 and TNF-α levels **(H)** Colon tissue levels of IL-18, IL-22, MUC2 and REG3γ were determined using standard ELISA methods. **(I-J)** Microphotographs of hematoxylin and eosin (H&E) stained **(I)** and Alcian Blue-Periodic Acid Schiff (AB-PAS) stained **(J)** sections of colons are shown. Inflammation and AB-PAS scores were shown. Scale bar indicates 100 μm. Statistics were performed using one-way ANOVA. Error bars, ±SEM; ns: Not significant, **p<0.01; ***p<0.001, ****p<0.0001. The actual p-values are provided in source data file. All experiments were repeated at least three times using biologically independent replicates, yielding similar results. Source data are provided as a Source data file.

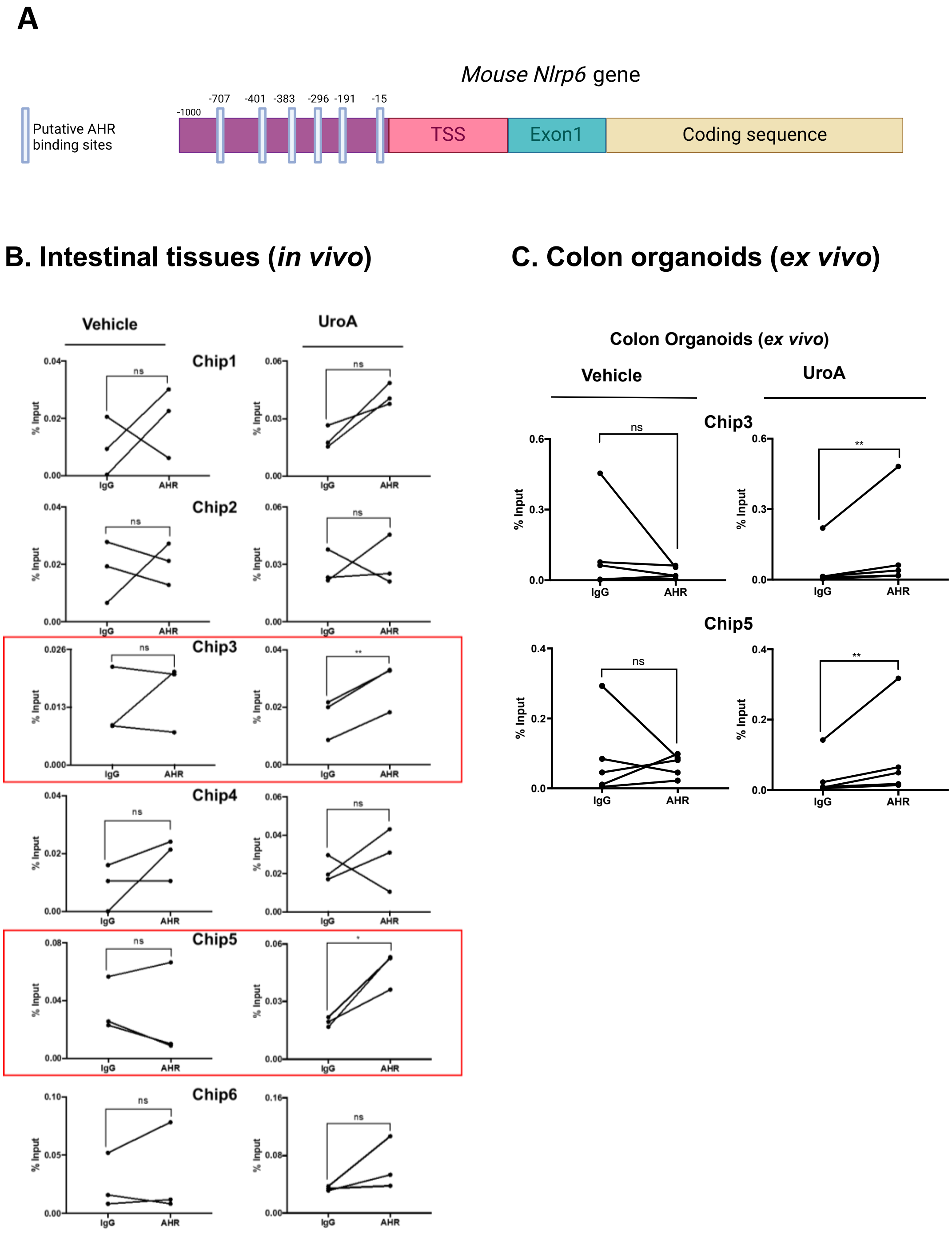

Figure S17. Putative AHR-binding sites in the promoter region of *Nlrp6* gene in colon organoids of C57BL/6 mice. **(A)** The putative AHR binding sites in the promoter region of mouse *Nlrp6* gene. **(B)** The small intestinal (SI) tissue (1 cm) of C57BL/6 mice was cultured for 24 h in presence of vehicle (0.05% DMSO) or UroA (25 µM). The SI tissue was processed for Chromatin immunoprecipitation (ChIP) assay with anti-AHR antibody. The AHR binding DNA was analyzed by qPCR to identify the 6 putative AHR-binding sites in the promoter region of *Nlrp6* gene. Data are presented as Mean± SEM in the graphs. (Paired Student t-test). **(C)** Colon organoids prepared

from C57BL/6 mice and treated with vehicle (0.05% DMSO) or UroA (25 μM) and processed for ChIP assay with anti-AHR antibody. The AHR-binding DNA sites (regions 3 and 5) was utilized for qPCR to identify the AHR binding site in the promoter region of the *Nlrp6* gene. Data are presented as Mean± SEM in the graphs. (Paired Student t-test). The actual p-values are provided in source data file. All experiments were repeated at least three times using biologically independent replicates, yielding similar results. Source data are provided as a Source data file.

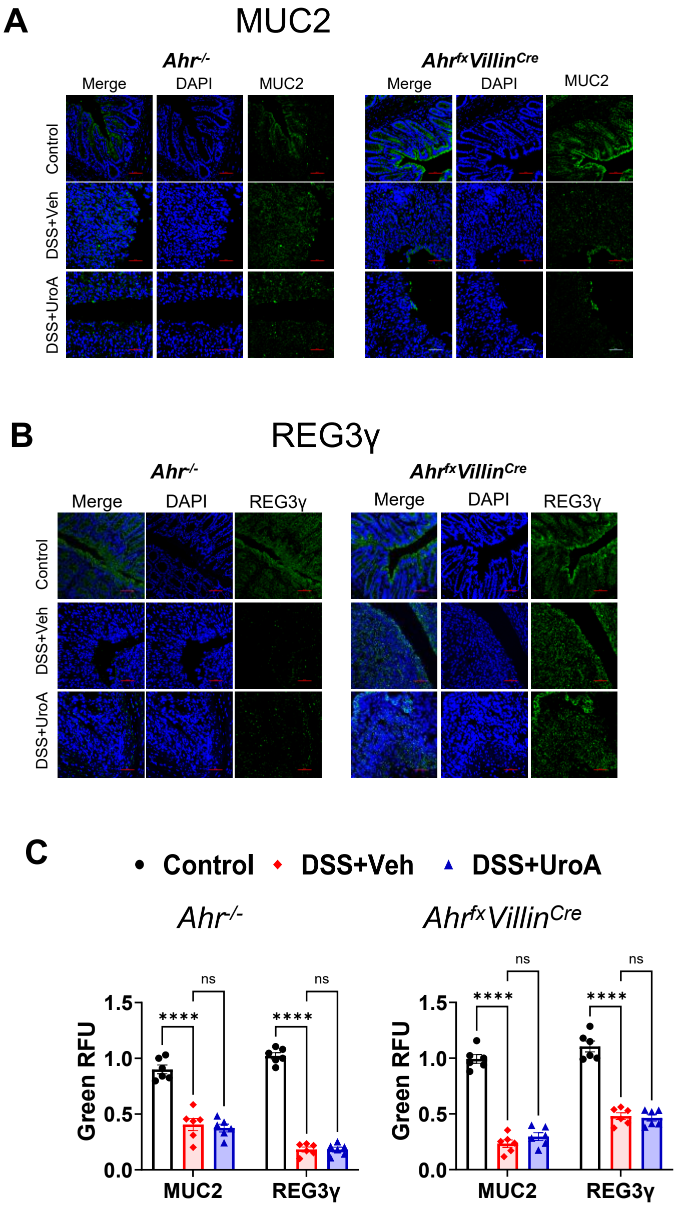

Figure S18. UroA induces MUC2 and REG3γ in AHR-dependent manner.
Confocal images of *AhR*$^{-/-}$ and *AhR*^{fx}-*Villin*^{Cre} mice colon sections stained with **(A)** anti-MUC2 antibody and **(B)** anti-REG3γ antibody followed by secondary antibody tagged with Alexa 488. Nucleus was stained with DAPI. The fluorescence images were captured using Nikon A1R confocal microscope. The scale bar indicates 50 µm. **(C)** The fluorescence intensity ($n = \sim 20$ cell) was measured using Nikon Elements. Statistics were performed by Two Way ANOVA test. ns: Not significant, ****$p<0.0001$**.** The actual p-values are provided in source data file. All experiments were repeated at least three times using biologically independent replicates, yielding similar results. Source data are provided as a Source data file.

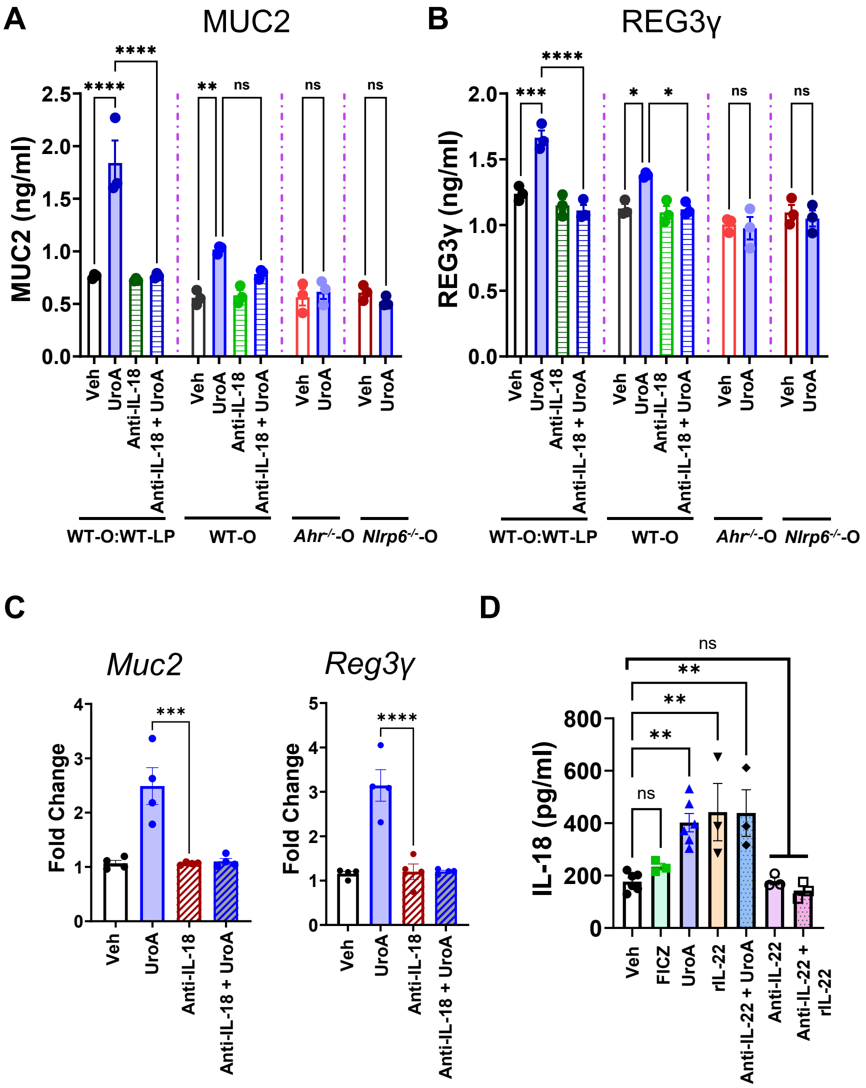

Figure S19. UroA induced MUC2 and REG3γ in an IL-18-dependent manner.
(A-B) Wildtype (WT) intestinal organoids (n= 100 organoids) (O): lamina propria (LP) co-cultures or organoids of WT or *Ahr*$^{-/-}$ or *Nlrp6*$^{-/-}$ were treated with vehicle (0.05% DMSO) or UroA (25 µM) for 72 h in presence or absence of anti- IL-18 Ab (20 ng/ml). The MUC2 (A) and REG3γ (B) protein levels in supernatant were measured by standard ELISA. **(C)** The fold changes in mRNA levels of *Muc2* and *Reg3γ* were measured by SYBR green RT-PCR method. **(D)** UroA induces IL-18 independent of IL-22**.** WT intestinal organoids were treated with FICZ (50 nM) or UroA (25 µM) or rIL-22 (10 ng/ml) in the presence/absence of anti-IL-22 ab (20 ng/mL). IL-18 levels were measured. Statistics were performed using one-way ANOVA. Error bars, ±SEM; ns: Not significant, *p<0.05, **p<0.01; ***p<0.001, ****p<0.0001. The actual p-values are provided in source data file. All experiments were repeated at least three times using biologically independent replicates, yielding similar results. Source data are provided as a Source data file.

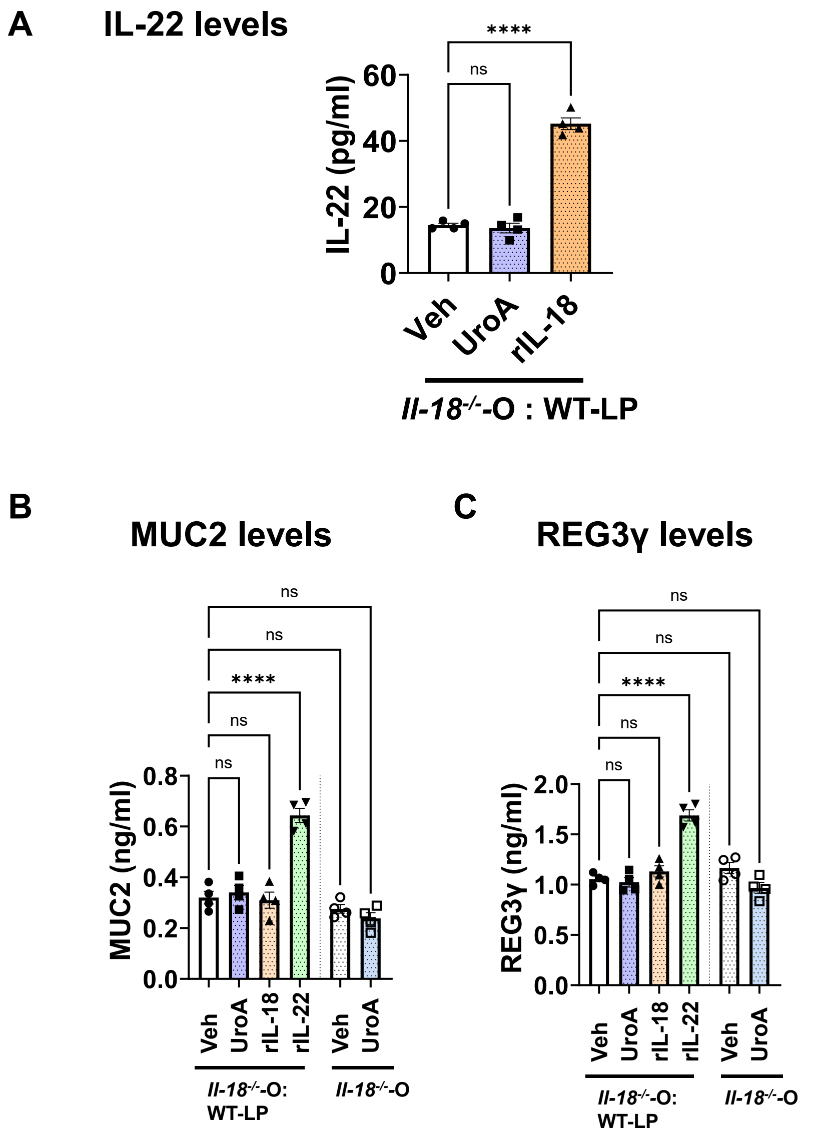

Figure S20. UroA requires IEC-IL-18 to induce IL-22, MUC2 and REG3γ.

(A) Co-cultures of *Il-18*$^{-/-}$ intestinal organoids (n= 100 organoids) (O): WT lamina propria (LP) cells (1X10^{5}) were treated with vehicle (0.05% DMSO) or UroA (25 μM) or recombinant IL-18 (10 ng/ml) for 72 h. IL-22 levels were measured by ELISA. **(B-C)** *Il-18*$^{-/-}$ intestinal organoids (n= 100 organoids) (O): WT lamina propria (LP) co-cultures or organoids of *Il-18*$^{-/-}$ were treated with vehicle (0.05% DMSO) or UroA (25 μM) or rIL-18 (10 ng/ml) or rIL-22 (10 ng/mL) for 72 h. The MUC2 (B) and REG3γ (C) protein levels in supernatant were measured by standard ELISA. Statistics were performed using one-way ANOVA. Error bars, ±SEM; ns: Not significant, ****$p<0.0001$. The actual p-values are provided in source data file. All experiments were repeated at least three times using biologically independent replicates, yielding similar results. Source data are provided as a Source data file.

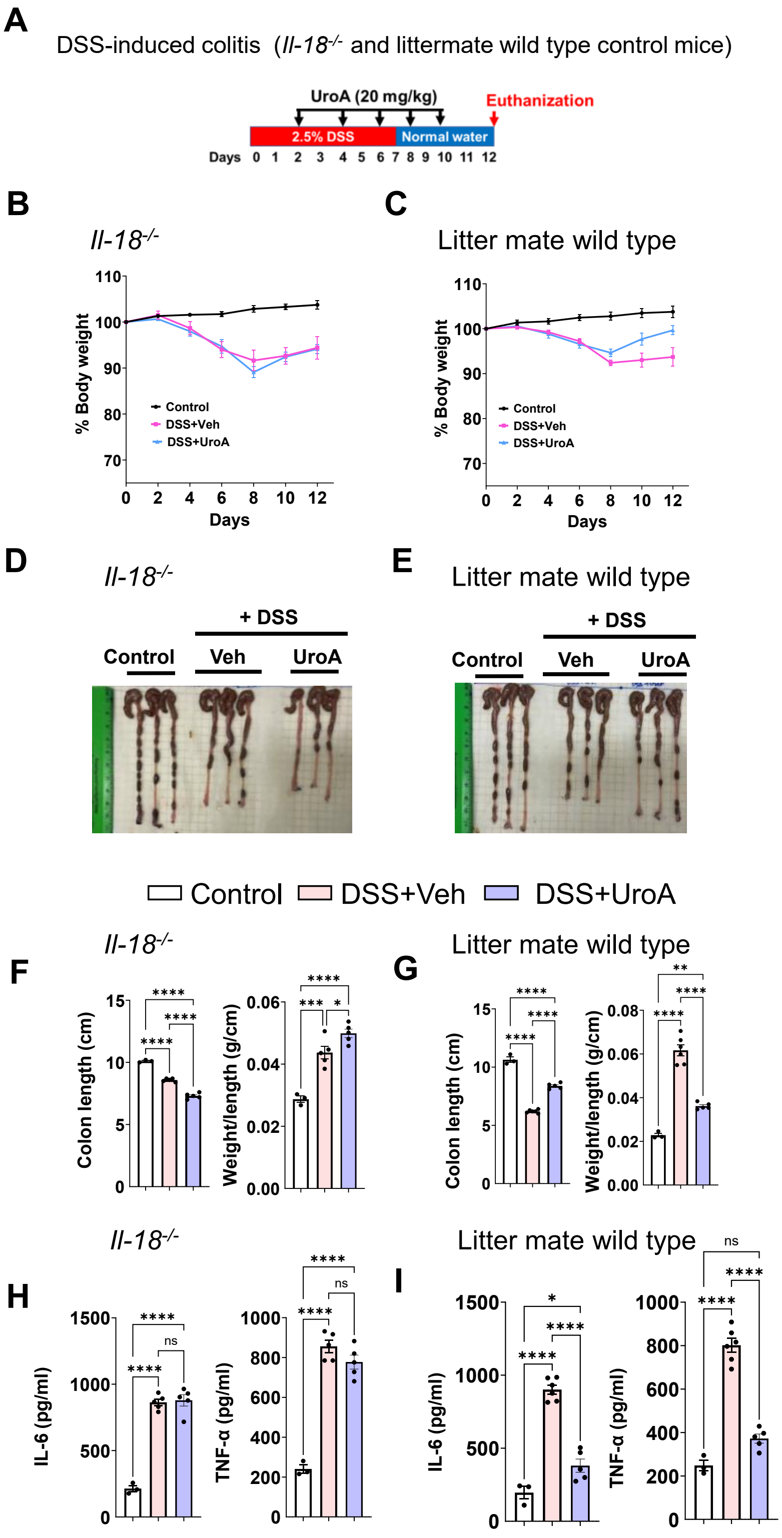

Figure S21. Littermate control experiment for *Il-18*$^{-/-}$ mice (Figure 7). IL-18 is required for UroA-mediated protective activities against DSS-induced colitis. (A) *Il-18*$^{+/+}$ *and Il-18*$^{-/-}$ mice (6–8-week age old, n=4-5 consisting of male (n=2) and female (n=2-3) mice per group) were either

on normal water (control group) or subjected to 2.5% DSS in drinking water for 7 days followed by 5 days of regular water. Both control and DSS-subjected mice were treated with either vehicle (1% CMC+0.1% Tween 80) or UroA (20 mg/kg) orally every 48 h of intervals. **(B-C)** Percent body weight loss of *Il-18*$^{-/-}$ and *Il-18*$^{+/+}$ mice. **(D-E)** Gross images of colons of *Il-18*$^{-/-}$ and *Il-18*$^{+/+}$ mice are represented. **(F-G)** Colon lengths and ratio of colon weight/length of *Il-18*$^{-/-}$ mice are shown. **(H-I)** Serum IL-6 and TNF-α levels of *Il-18*$^{-/-}$ and *Il-18*$^{+/+}$ mice were measured using standard ELISA. Statistics were performed using one-way ANOVA. Error bars, ±SEM; ns: Not significant, **p<0.01; ***p<0.001, ****p<0.0001. The actual p-values are provided in source data file. All experiments were repeated at least three times using biologically independent replicates, yielding similar results. Source data are provided as a Source data file.

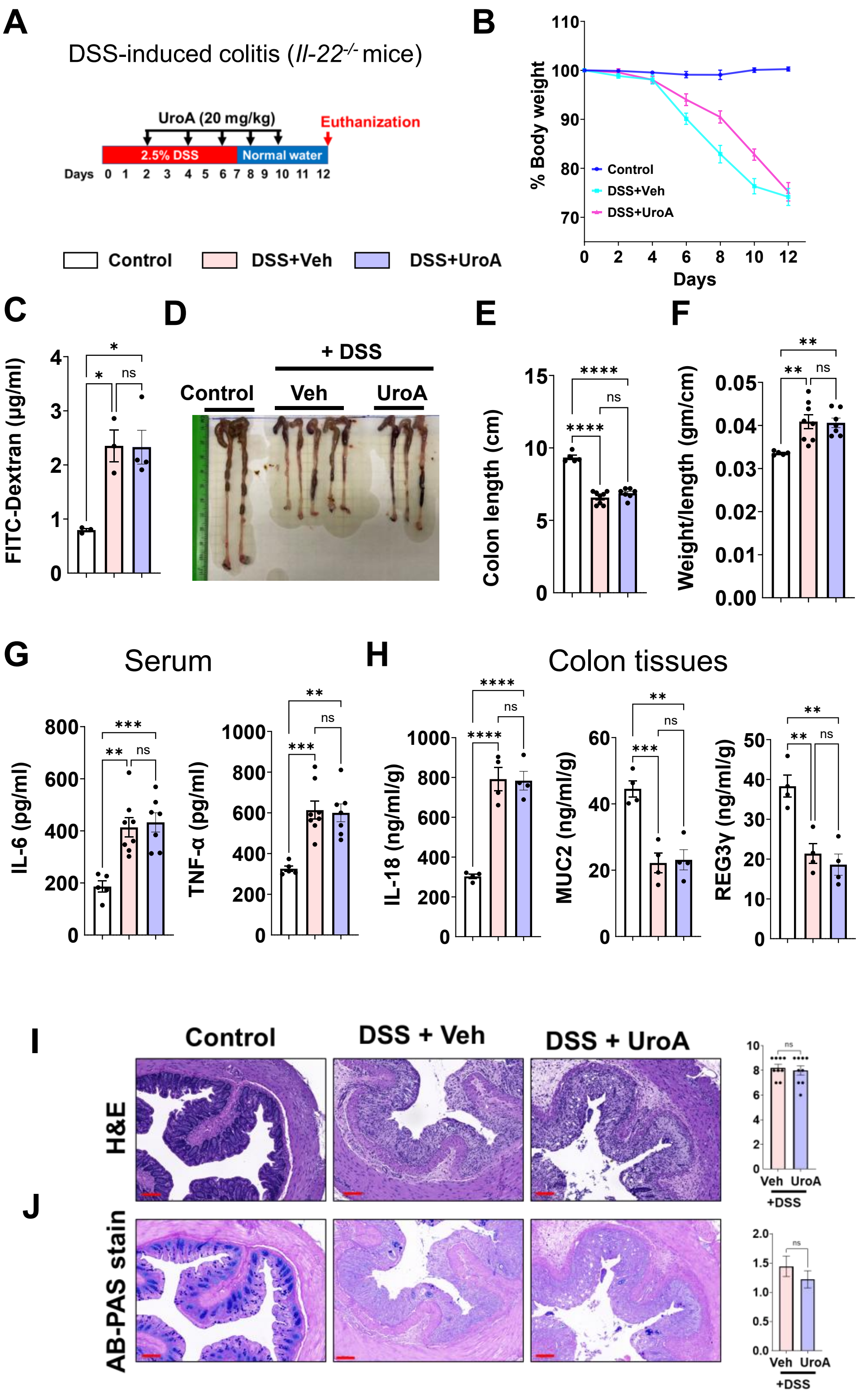

Figure S22. UroA requires IL-22 to protect against DSS-induced colitis. **(A)** *Il-22*$^{-/-}$ mice (6–8-week age old, n=5-8 consisting of male (n=3-4) and female (n=2-4) mice per group) were either

on normal water (control group) or subjected to 2.5% DSS in drinking water for 7 days followed by 5 days of regular water. Both control and DSS-subjected mice were treated with either vehicle (1% CMC+0.1% Tween 80) or UroA (20 mg/kg) orally every 48 h of intervals. **(B)** Percent body weight loss of *Il-22*$^{-/-}$ mice. **(C)** Intestinal permeability was assessed by using FITC-dextran intestinal permeability assay as described in methods. **(D)** Gross images of colons are represented. **(E-F)** Colon lengths **(E)** and ratio of colon weight/length **(F)** of *Il-22*$^{-/-}$ mice are shown. **(G)** Serum IL-6 and TNF-α levels and **(H)** colonic IL-18, MUC2 and REG3γ were measured using standard ELISA. Microphotographs of hematoxylin and eosin (H&E) stained **(I)** and Alcian Blue-Periodic Acid Schiff (AB-PAS) stained **(J)** sections of colons are shown. Inflammation and AB-PAS scores were shown. Scale bar indicates 100 µm. Statistics were performed using one-way ANOVA. Error bars, ±SEM; ns: Not significant, **p<0.01; ***p<0.001, ****p<0.0001. The actual p-values are provided in source data file. All experiments were repeated at least three times using biologically independent replicates, yielding similar results. Source data are provided as a Source data file.

Littermate wildtype for *Il-22*$^{-/-}$ mice

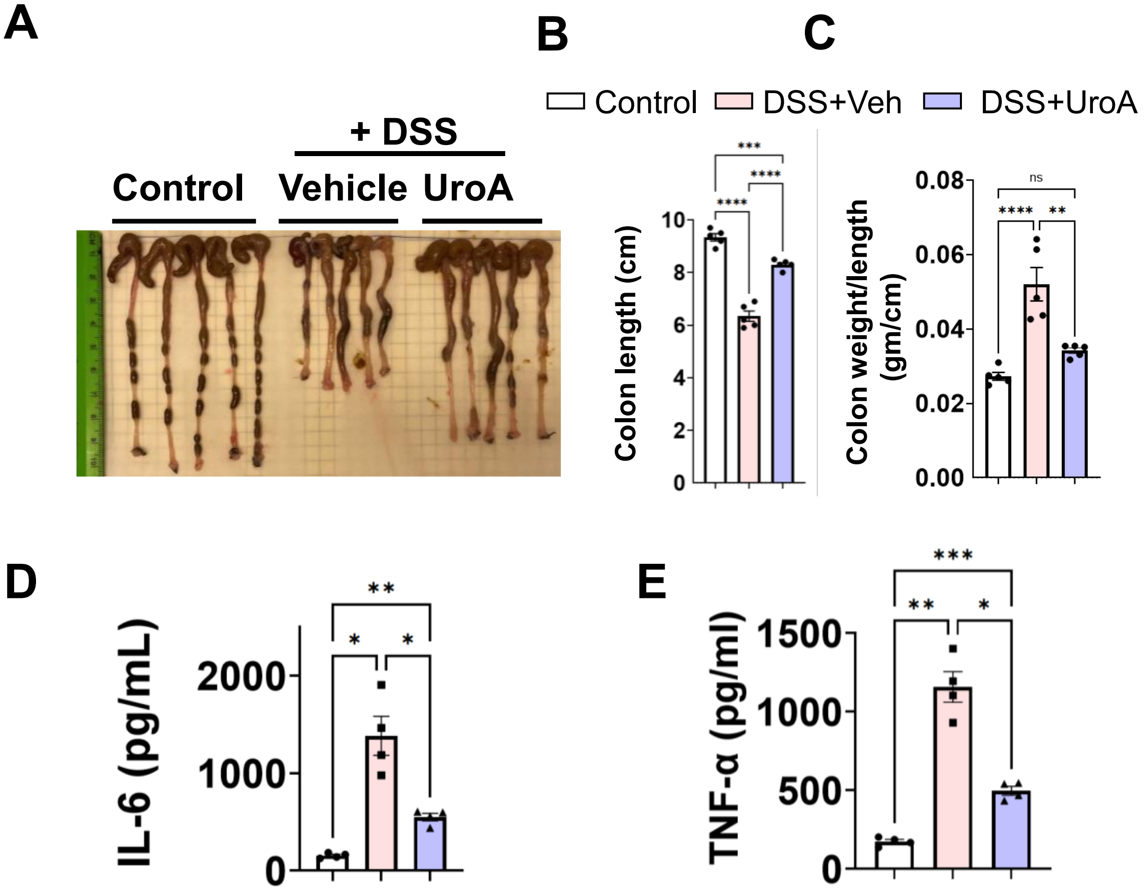

Figure S23. Littermate wild type (*Il-22*$^{+/+}$) control for *Il-22*$^{-/-}$ mice. (A) Littermate wild type *Il-22*$^{++}$ mice (6–8-week age old, n=5 consisting of male (n=2) and female (n=3) mice per group) were either on normal water (control group) or subjected to 2.5% DSS in drinking water for 7 days followed by 5 days of regular water. Both control and DSS-subjected mice were treated with either vehicle (1% CMC+0.1% Tween 80) or UroA (20 mg/kg) orally every 48 h of intervals. **(A)** Gross images of colons are represented. **(B-C)** Colon lengths **(B)** and ratio of colon weight/length **(C)** of *Il-22*$^{+/+}$ mice are shown. **(D-E)** Serum IL-6 and TNF-α levels are measured by standard ELISA. Statistics were performed using one-way ANOVA. Error bars, ±SEM; ns: Not significant, **$p<0.01$; ***$p<0.001$, ****$p<0.0001$. The actual p-values are provided in source data file. All experiments were repeated at least three times using biologically independent replicates, yielding similar results. Source data are provided as a Source data file.

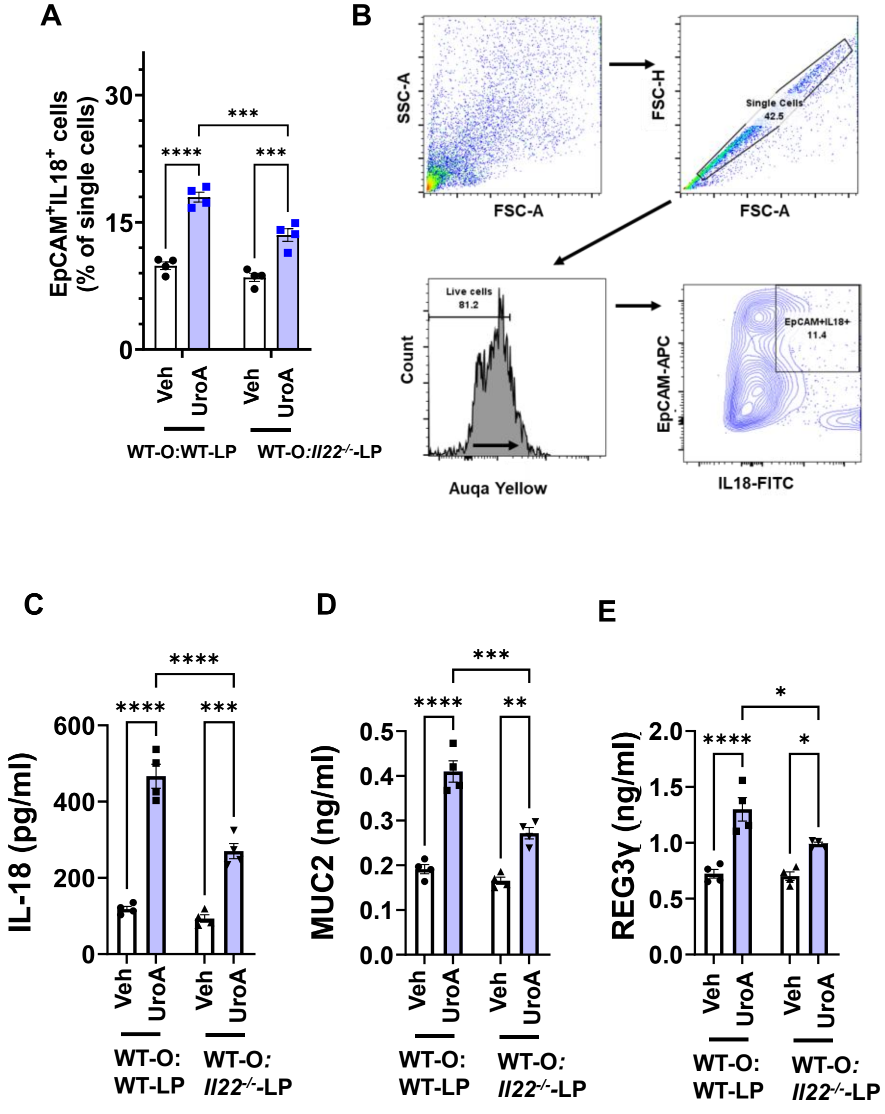

Figure S24. UroA induced IL-18 from epithelial cells independent of IL-22 (A-B) Co-cultures of WT intestinal organoids (WT-O) (n= 100 organoids) with WT or *Il22*$^{-/-}$ lamina propria (LP) cells (1X10^{5}) were treated with vehicle (0.05% DMSO) or UroA (25 μM) for 72 h. Expression of IL-18 levels in epithelial cells (EPCAM^{+}) was measured using flow cytometry as described in methods. (C) The supernatants from above co-cultures were collected and measure the levels of (C) IL-18, (D) MUC2 and (E) REG3γ by standard ELISA. Statistics were performed using one-way ANOVA. Error bars, ±SEM; ns: Not significant, *P<0.05; **p<0.01; ***p<0.001, ****p<0.0001. The actual p-values are provided in source data file. All experiments were repeated at least three times using biologically independent replicates, yielding similar results. Source data are provided as a Source data file.

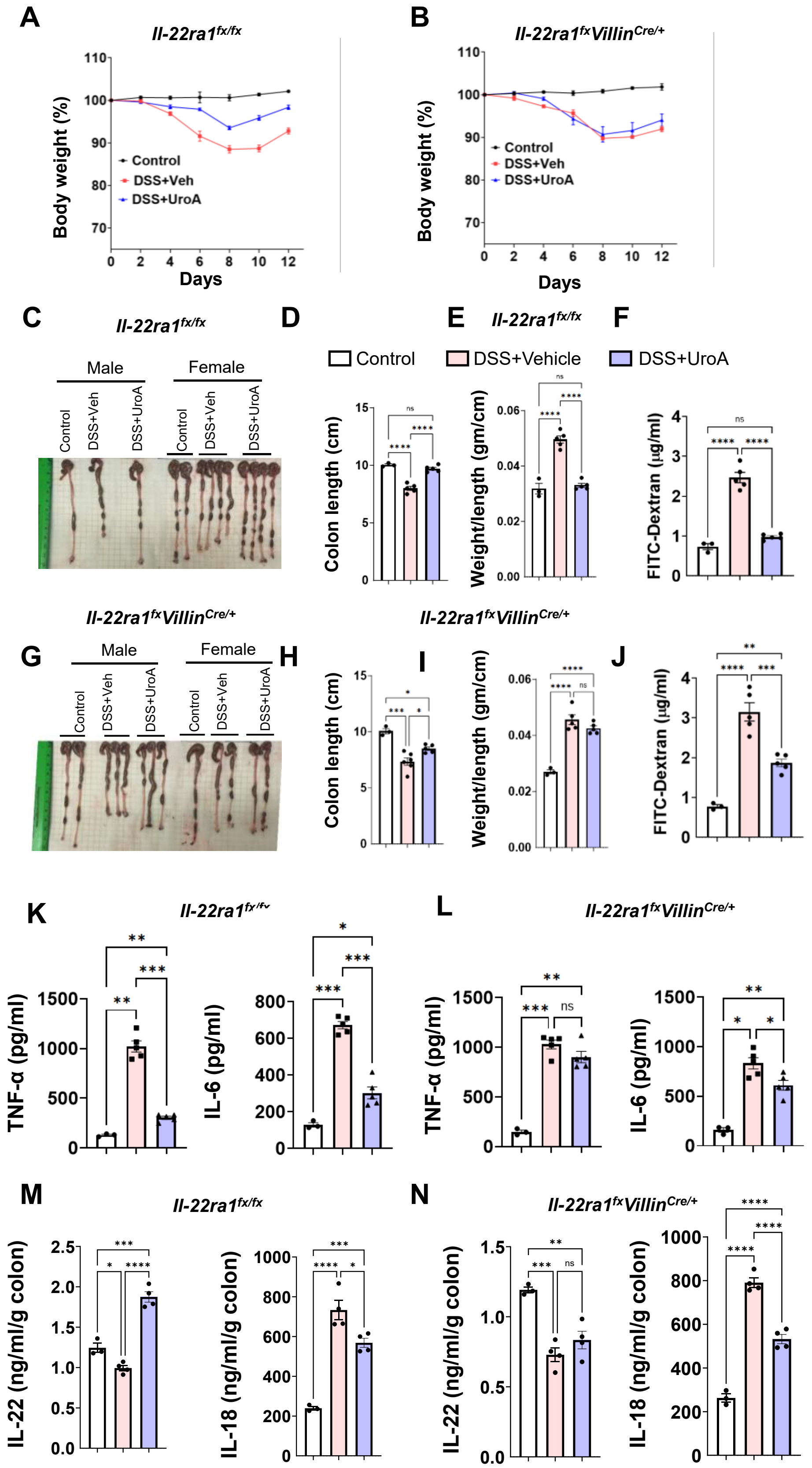
A
Il-22ra1fx/fx
Body weight (%)
Days
Control
DSS+Veh
DSS+UroA
B
Il-22ra1fxVillinCre/+
Body weight (%)
Days
Control
DSS+Veh
DSS+UroA
C
Il-22ra1fx/fx
Male
Female
Control
DSS+Veh
DSS+UroA
D
E
Il-22ra1fx/fx
F
Control
DSS+Vehicle
DSS+UroA
Colon length (cm)
Weight/length (gm/cm)
FITC-Dextran (µg/ml)
Il-22ra1fxVillinCre/+
G
Male
Female
Control
DSS+Veh
DSS+UroA
H
I
J
Colon length (cm)
Weight/length (gm/cm)
FITC-Dextran (µg/ml)
K
Il-22ra1fx/fx
TNF-α (pg/ml)
IL-6 (pg/ml)
L
Il-22ra1fxVillinCre/+
TNF-α (pg/ml)
IL-6 (pg/ml)
M
Il-22ra1fx/fx
IL-22 (ng/ml/g colon)
IL-18 (ng/ml/g colon)
N
Il-22ra1fxVillinCre/+
IL-22 (ng/ml/g colon)
IL-18 (ng/ml/g colon)

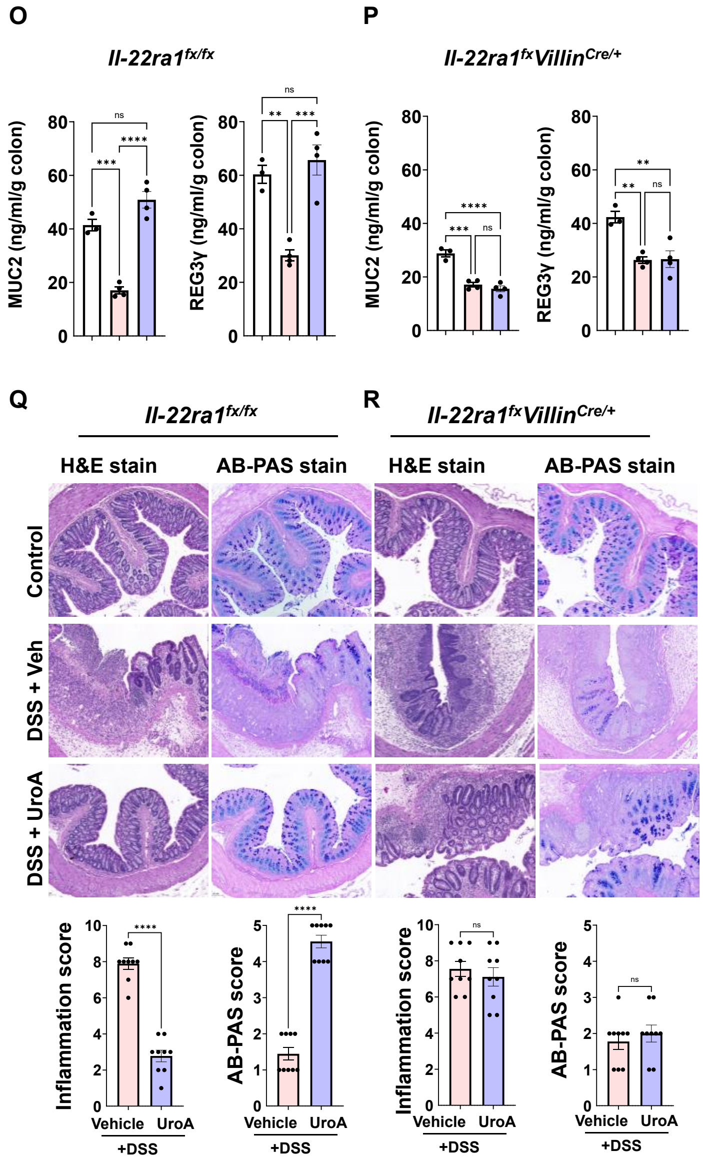

Figure S25. UroA partially protects *Il-22ra1^{fx}Villin$^{Cre/+}$* mice against DSS-induced colitis. *Il-22ra1^{fx}Villin$^{Cre/+}$* and littermate mice *Il-22ra1$^{fx/fx}$* (6–8-week age old, n=5-6 consisting of male (n=2-3) and female (n=3) mice per group) were either on normal water (control group) or subjected to 2.5% DSS in drinking water for 7 days followed by 5 days of regular water. Both control and DSS-subjected mice were treated with either vehicle (1% CMC+0.1% Tween 80) or UroA (20 mg/kg) orally every 48 h of intervals. **(A-B)** Percent body weight loss of *Il-22ra1$^{fx/fx}$* and *Il-22ra1^{fx}Villin$^{Cre/+}$* mice are shown. Gross images of colons **(C)**, colon lengths **(D)**, weight/length ratio **(E)** and intestinal permeability **(F)** of *Il-22ra1$^{fx/fx}$* mice are shown. Gross images of colons **(G)**, colon lengths **(H)**, weight/length ratio **(I)** and intestinal permeability **(J)** of *Il-22ra1^{fx}Villin$^{Cre/+}$* mice are shown. **(K)** Serum IL-6 and TNF-α levels *Il-22ra1$^{fx/fx}$* mice are shown. **(L)** Serum IL-6 and TNF-α levels of *Il-22ra1^{fx}Villin$^{Cre/+}$* mice are shown. **(M)** IL-22 and IL-18 in colons of *Il-22ra1$^{fx/fx}$* mice were measured using standard ELISA. **(N)** IL-22 and IL-18 in colons of *Il-22ra1^{fx}Villin$^{Cre/+}$* mice were measured using standard ELISA**. (O)** MUC2 and REG3γ in colons of

Il-22ra1$^{fx/fx}$ mice were measured using standard ELISA. **(P)** MUC2 and REG3γ in colons of *Il-22ra1^{fx}Villin$^{Cre/+}$* mice were measured using standard ELISA. Microphotographs of hematoxylin and eosin (H&E) stained **(Q-R left panel)** and Alcian Blue-Periodic Acid Schiff (AB-PAS) stained **(Q-R right panel)** sections of colons are shown. Inflammation and AB-PAS scores were shown. Scale bar indicates 100 µm. Statistics were performed using one-way ANOVA. Error bars, ±SEM; ns: Not significant, *p<0.05; **p<0.01; ***p<0.001, ****p<0.0001. The actual p-values are provided in source data file. All experiments were repeated at least three times using biologically independent replicates, yielding similar results. Source data are provided as a Source data file.

Gating strategy for human biopsies cell samples

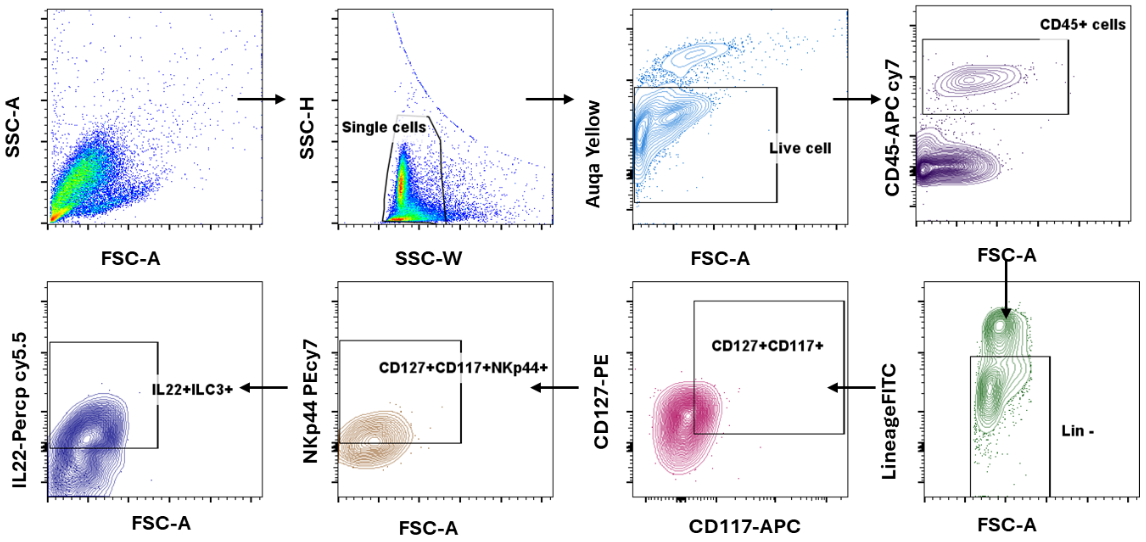

Figure S26. Gating strategy for IL-22^{+}-ILC3 cells from intestinal biopsies. Lamina propria single-cell suspensions of intestinal biopsies were gated with the forward scatter/side scatter (FSC/SSC) strategy. Single and live cells were only considered for the analysis. Live CD45^{+}LIN^{-}CD127^{+}CD117^{+}NKp44^{+} were used to gate populations of human ILC3s. IL-22 expression was analyzed in ILC3 populations. All flow cytometry experiments were performed using a BD canto flow cytometer and the FACS Diva software (BD Biosciences) and analyzed with FlowJo v.10 software (TreeStar).

Table S1. Key resources table

REAGENT or RESOURCE	SOURCE	IDENTIFIER	Dilution
**Antibodies**			
AHR Polyclonal antibody	Proteintech	Cat# 17840-1-AP	1:1000
IL-18 Polyclonal antibody	Proteintech	Cat# 10663-1-AP	1:1000
MUC2 Polyclonal antibody	Proteintech	Cat# 27675-1-AP	1:1000
Reg3G Antibody	Novus Biologicals	Cat# NBP2-93737	1:1000
NLRP6 antibody	Sigma	Cat# SAB1302240	1:1000
Goat anti-rabbit IgG (H + L), HRP conjugate	Proteintech	Cat# SA00001-2	1:5000
HRP-conjugated Beta Actin Monoclonal antibody	Proteintech	Cat# HRP-60008	1:5000
Goat anti-Rabbit IgG (H+L) Cross-Adsorbed Secondary Antibody, Alexa Fluor™ 488	Invitrogen	Cat# A-11008	1:5000
APC/Cyanine7 anti-mouse CD45.2 Antibody	Biolegend	Cat# 109824	1:100
FITC anti-mouse Lineage Cocktail with Isotype Ctrl	Biolegend	Cat# 133302	1:100
PerCP/Cyanine5.5 anti-mouse CD335 (NKp46) Antibody	Biolegend	Cat# 137610	1:100
Alexa Fluor® 647 Mouse Anti-Mouse RORγt	BD Pharmingen	Cat# 562682	1:100
Mouse IL-22 PE-conjugated Antibody	R&D Systems™	Cat# IC582P	1:100
PE anti-mouse IL-22 Antibody	Biolegend	Cat# 516404	1:100
Alexa Fluor® 647 anti-mouse CD326 (Ep-CAM) Antibody	Biolegend	Cat# 118212	1:100
FITC Donkey anti-rabbit IgG (minimal x-reactivity) Antibody	Biolegend	Cat# 406403	1:100
APC/Cyanine7 anti-mouse CD45 Antibody	Biolegend	Cat# 103115	1:100
PE/Cyanine7 anti-mouse CD11c Antibody	Biolegend	Cat# 117317	1:100
PE anti-mouse F4/80 Recombinant Antibody	Biolegend	Cat# 157304	1:100
APC/Cyanine7 anti-human CD45 Antibody	Biolegend	Cat# 304014	1:100
FITC anti-human Lineage Cocktail (CD3, CD14, CD16, CD19, CD20, CD56)	Biolegend	Cat# 348801	1:100

PE/Cyanine7 anti-human CD336 (NKp44) Antibody	Biolegend	Cat# 325115	1:100
PE anti-human CD127 (IL-7Rα) Antibody	Biolegend	Cat# 351340	1:100
APC anti-human CD117 (c-kit) Antibody	Biolegend	Cat# 375307	1:100
PerCP/Cyanine5.5 anti-human IL-22 Antibody	Biolegend	Cat# 366709	1:100
Alexa Fluor® 647 anti-human CD326 (EpCAM) Antibody	Biolegend	Cat# 369819	1:100
Mouse IL-23 p19 Antibody	R&D Systems	Cat# AF1619SP	1:100
IL-22 Monoclonal Antibody	eBioscience	Cat# 16-7222-82	1:100
InVivoMAb anti-mouse/rat IL-1β	Bio X Cell	Cat# BE0246	1:100
InVivoMAb anti-mouse IL-18	Bio X Cell	Cat# BE0237	1:100
BD Pharmingen™ PerCP-Cy™5.5 Mouse Anti-Mouse CD45.2	BD	Cat# 552950	1:100
BD Horizon™ BUV395 Rat Anti-Mouse CD45	BD	565967	1:100
BD Horizon™ BV421 Hamster Anti-Mouse CD3e	BD	Cat# 562600	1:100
BD Horizon™ BV421 Rat Anti-Mouse CD19	BD	Cat# 562701	1:100
BD Horizon™ BV421 Rat Anti-Mouse Ly-6G and Ly-6C	BD	Cat# 562709	1:100
BD Horizon™ BV421 Rat Anti-Mouse F4/80	BD	Cat# 565411	1:100
BD Horizon™ BUV395 Mouse Anti-GATA3	BD	Cat# 565448	1:100
BD Pharmingen™ PE-Cy™7 Mouse anti-GATA3	BD	560405	1:100
Brilliant Violet 650™ anti-mouse CD127 (IL-7Rα) Antibody	Biolegend	Cat# 135043	1:100
APC anti-mouse CD196 (CCR6) Antibody	Biolegend	Cat# 129814	1:100
Brilliant Violet 421™ anti-mouse TCR γ/δ Antibody	Biolegend	Cat# 118120	1:100
Brilliant Violet 421™ anti-mouse TCR β chain Antibody	Biolegend	Cat# 109230	1:100
Brilliant Violet 421™ anti-mouse CD5 Antibody	Biolegend	Cat# 100629	1:100
PE/Cyanine7 anti-mouse TCR γ/δ Antibody	Biolegend	Cat# 118124	1:100
APC/Cyanine7 anti-mouse CD8a Recombinant Antibody	Biolegend	Cat# 155016	1:100

ROR gamma (t) Monoclonal Antibody (B2D), Alexa Fluor™ 488, eBioscience™	Thermo Fisher Scientific	Cat# 53-6981-82	1:100
BD Pharmingen™ PerCP-Cy™5.5 Mouse Anti-Mouse RORγt	BD	562683	1:100
IL-22 Monoclonal Antibody (1H8PWSR), PE, eBioscience™	Thermo Fisher Scientific	Cat# 12-7221-82	1:100
EOMES Monoclonal Antibody (Dan11mag), PE-eFluor™ 610, eBioscience™	Thermo Fisher Scientific	Cat# 61-4875-82	1:100
BD Pharmingen™ Alexa Fluor™ 488 Mouse Anti-EOMES	BD	567169	1:100
KLRG1 Monoclonal Antibody (2F1), APC-eFluor™ 780, eBioscience™	Thermo Fisher Scientific	Cat# 47-5893-82	1:100
CD4 Monoclonal Antibody (GK1.5), Alexa Fluor™ 700, eBioscience™	Thermo Fisher Scientific	Cat# 56-0041-82	1:100
CD335 (NKp46) Monoclonal Antibody (29A1.4), PE-Cyanine7, eBioscience™	Thermo Fisher Scientific	Cat# 25-3351-82	1:100
CD335 (NKp46) Monoclonal Antibody (29A1.4), PE-eFluor™ 610, eBioscience™	Thermo Fisher Scientific	61-3351-82	1:100
CD90.2 (Thy-1.2) Monoclonal Antibody (53-2.1), eFluor™ 506, eBioscience™	Thermo Fisher Scientific	69-0902-82	1:100
LIVE/DEAD™ Fixable Yellow Dead Cell Stain Kit, for 405 nm excitation	Thermo Fisher Scientific	Cat# L34959	1:100
IL-17A Monoclonal Antibody (eBio17B7), APC, eBioscience™	Thermo Fisher Scientific	Cat# 17-7177-81	1:100
**Bacterial and Virus Strains**			
Citrobacter rodentium DBS100	Dr. Gabriel Núñez, University of Michigan, USA	N/A	
**Chemicals, Peptides, and Recombinant Proteins**			
Lipopolysaccharides from Escherichia coli O111:B4	Sigma	Cat# L2630	
Phosphate-Buffered Saline	Corning	Cat# 21040CV	

Dextran sulfate sodium salt, colitis grade (36,000 - 50,000)	MP Bio	Cat# 160110-100 g
Sodium carboxymethyl cellulose	Sigma	Cat# 419273
Polysorbate 80	Sigma	Cat# 59924
RPMI medium	Corning	Cat# 10-040-CV
Fetal Bovine Serum	Gibco	Cat# 6140079
Penicillin-Streptomycin, Solution	Sigma	Cat# P4333
RNAlater™ Stabilization Solution	ThermoFisher	Cat# AM7020
Prefilled 10% Neutral Buffered Formalin	VWR	Cat# 16004-115
Fluorescein isothiocyanate–dextran	Sigma	Cat# FD-4
Alcian blue 1% in 3% acetic acid pH 2.5	Electron Microscopy Sciences	Cat# 26026-13
Periodic acid 0,5% in aqueous solution	Electron Microscopy Sciences	Cat# 19324-05
Schiff reagent	Electron Microscopy Sciences	Cat# 100504-432
Nuclear Fast Red solution	Electron Microscopy Sciences	Cat# 26078-05
Xylene Substitute Mountant	Epredia	Cat# 1900231
VECTASHIELD HardSet Antifade mounting medium with DAPI	VectorLaboratories	Cat# H-1500
Recombinant Mouse M-CSF	Biolegend	Cat# 576406
TRIzol™ Reagent	Invitrogen	Cat# 15596026
RNeasy Mini Kit	Qiagen	Cat# 74104
RNase-Free DNase Set	Qiagen	Cat# 79254
Maxwell® 16 LEV simplyRNA Purification Kits	Promega	Cat# AS1270
Maxwell 16 LEV simplyRNA Tissue Ki	Promega	Cat# AS1280
High-Capacity cDNA Reverse Transcription Kit with RNase Inhibitor	Applied Biosystems	Cat# 4374966
PowerUp™ SYBR™ Green Master Mix	Applied Biosystems	Cat# A25742
Nuclease-Free Water	Invitrogen	Cat# AM9937
Lamina Propria Dissociation Kit, mouse	Miltenyi Biotech	Cat# 130-097-410
Recombinant Mouse IL-23	R&D Systems™	Cat# 1887-ML
Mouse Recombinant IL-1β	Stemcell Technologies	Cat# 78035.1
Brefeldin A Solution	Biolegend	Cat# 420601

True-Nuclear™ Transcription Factor Buffer Set	Biolegend	Cat# 424401
IntestiCult™ Organoid Growth Medium, Mouse	Stemcell Technologies	Cat# 06005
Matrigel® Matrix GFR	Corning	Cat# 356231
Cryostor CS10	Stemcell Technologies	Cat# 07941
Gentle Cell Dissociation Reagent	Stemcell Technologies	Cat# 100-0485
Dulbecco's modified Eagle's medium-high glucose	Sigma	Cat# D6429
Sodium pyruvate solution	HyClone	Cat# SH30239.01
2-mercaptoethanol	Gibco	Cat# 21985023
Recombinant Mouse IL-7	Biolegend	Cat# 577802
Recombinant Mouse IL-15	Biolegend	Cat# 566302
TrypLE™ Express Enzyme	Gibco	Cat# 12604013
Cell recovery solution	Corning	Cat# 354253
Ethylenediaminetetraacetic acid disodium salt solution	Sigma	Cat# E7889
Dithiothreitol solution	Sigma	Cat# 43816
Collagenase D	Roche	Cat# 11088866001
Trypsin inhibitor	Gibco	Cat# R007100
Recombinant Human IL-23	Biolegend	Cat# 574102
Recombinant Human IL-1β	Biolegend	Cat# 579402
Fixation Buffer	Biolegend	Cat# 420801
Intracellular Staining Permeabilization Wash Buffer (10X)	Biolegend	Cat# 421002
Dimethyl sulfoxide	Sigma	Cat# D4540
7-AAD Viability Staining Solution	Biolegend	Cat# 420403
Percoll®	Sigma	Cat# P4937-100ML
Cell Activation Cocktail (without Brefeldin A)	Biolegend	Cat# 423301
Cell Staining Buffer	Biolegend	Cat# 423301
Zombie Violet™ Fixable Viability Kit	Biolegend	Cat# 423113
eBioscience™ Foxp3 / Transcription Factor Staining Buffer Set	Invitrogen	Cat# 00-5523-00
FICZ	Sigma	Cat# SML1489
TCDD	AccuStandard	Cat# D-404N
Adenosine 5′-triphosphate disodium salt hydrate	Sigma	Cat# A6419
Taurine	Sigma	Cat# T0625

Nigericin	Tocris Bioscience	Cat# 4312
CH-22319	Sigma	Cat# C8124
MCC950	Invivogen	Cat# INH-MCC
Recombinant Mouse IL-22	Novus Biologicals	Cat# NBP2-35122
Recombinant Mouse IL-18	BioLegend	Cat# 767002
**Critical Commercial Assays**		
ELISA MAX™ Standard Set Mouse TNF-α	BioLegend	Cat# 430901
ELISA MAX™ Standard Set Mouse IL-6	BioLegend	Cat# 431301
Myeloperoxidase (MPO) Activity Assay Kit	Abcam	Cat# ab105136
Pierce™ BCA Protein Assay Kit	Thermo Fisher Scientific	Cat# 23227
Mouse Regenerating islet-derived protein 3-gamma ELISA Kit	MyBioSource	Cat# MBS2882993
Mouse MUC2 (Mucin 2) ELISA Kit	MyBioSource	Cat# MBS8801203
ELISA MAX™ Deluxe Set Mouse IL-22	BioLegend	Cat# 436304
Mouse IL-18 Uncoated ELISA Kit	Invitrogen	Cat# 88-50618-88
ELISA MAX™ Deluxe Set Mouse IL-1β	BioLegend	Cat# 432604
IL-23 ELISA MAX™ Deluxe ELISA Kit	BioLegend	Cat# 433704
Caspase-Glo® 1 Inflammasome Assay	Promega	Cat# G9951
SimpleChIP® Enzymatic Chromatin IP Kit (Magnetic Beads) #9003	Cell Signaling Technology	Cat# 9003S
**Experimental Models: Cell Lines**		
MNK-3 cell	Dr. James Carlyle, University of Toronto, Canada	N/A
WT murine intestinal organoids	This paper	N/A
Ahr$^{-/-}$ murine intestinal organoids	This paper	N/A
Il18$^{-/-}$ murine intestinal organoids	This paper	N/A
Nlrp3$^{-/-}$ murine intestinal organoids	This paper	N/A
Nlrp6$^{-/-}$ murine intestinal organoids	This paper	N/A
**Experimental Models: Organisms/Strains**		

C57BL/6 WT	The Jackson Laboratory	Strain# 000664
C57BL/6-*Ahr*$^{tm1.2Arte}$	Taconic Laboratories	Model# 9166
B6.129P2-*Il18*^{tm1Aki}/J	The Jackson Laboratory	Strain# 004130
Ahr$^{tm3.1Bra}$/J	The Jackson Laboratory	Strain# 006203
B6.Cg-Tg(Vil1-cre)997Gum/J	The Jackson Laboratory	Strain# 004586
B6.129P2-*Lyz2*$^{tm1(cre)Ifo}$/J	The Jackson Laboratory	Strain# 004781
Ahr^{fx}-*Villin*^{Cre}	This paper	N/A
Ahr^{fx}-*LysM*^{Cre}	This paper	N/A
Nlrp6^{fx}	Dr. Daniel Mucida, Rockefeller University	N/A
Nlrp6^{fx}-*Villin*^{Cre}	This paper	N/A
Ill-22$^{-/-}$	Dr. Misty Good , University of North Carolina	N/A
Il22Ra1^{fx}	Dr. Pawan Kumar (Stony Brook University	N/A
Il22Ra1^{fx}-*Villin*^{Cre}	This paper	N/A
**Oligonucleotides**		
Mus musculus Il-22-Forward- 5'-TTG AGG TGT CCA ACT TCC AGC A-3'	IDT	N/A
Mus musculus Il-22-Reverse- 5'-AGC CGG ACG TCT GTG TTG TTA-3'	IDT	N/A
Mus musculus Il-18-Forward- 5'-CAG TGA ACC CCA GAC CAG AC-3'	IDT	N/A
Mus musculus Il-18-Reverse- 5'-GCC AAG CAA GAA AGT GTC CT-3'	IDT	N/A
Mus musculus Nlrp6-Forward- 5'-CAG ACG CTG TGG ACC TTG T-3'	IDT	N/A
Mus musculus Nlrp6-Reverse- 5'-ACG TGC TCG CGG TAC TTC TT-3'	IDT	N/A

Mus musculus Reg3γ-Forward- 5'-GCT CCT ATT GCT ATG CCT TGT TTA G-3'	IDT	N/A
Mus musculus Reg3γ-Reverse- 5'-CAT GGA GGA CAG GAA GGA AGC-3'	IDT	N/A
Mus musculus Muc2	RealTimePrimers.com	N/A
Mus musculus Actb	RealTimePrimers.com	Cat# VMPS-96
Mus musculus Gapdh	RealTimePrimers.com	Cat# VMPS-7317
Mus musculus cyp1a1	RealTimePrimers.com	Cat# VMPS-1512
Mus musculus AhR-NLRP6 -CHIP 1 S -CAT TTG ATA TTT GAG AGC ACG GT	IDT	N/A
Mus musculus AhR-NLRP6 -CHIP 1 AS -TGG AAA GAG AAG AAC TCC ACA A	IDT	N/A
Mus musculus AhR-NLRP6 -CHIP 2 S -TTA GTC TAC GAC AAA CAG TCA CTT A	IDT	N/A
Mus musculus AhR-NLRP6 -CHIP 2 AS-GGG ATA TGG AGT TTC ACT GTG T	IDT	N/A
Mus musculus AhR-NLRP6 -CHIP 3 S-AAC TTA GCC ATG TGG AGG TG	IDT	N/A
Mus musculus AhR-NLRP6 -CHIP 3 AS-GAC TGC AGG GAT ATG GAG TTT	IDT	N/A
Mus musculus AhR-NLRP6 -CHIP 4 S-AGG GCC TAG GGT CAG AAA	IDT	N/A
Mus musculus AhR-NLRP6 -CHIP 4 AS-CTC AGC TCT GCA GTC AGT TAA T	IDT	N/A
Mus musculus AhR-NLRP6 -CHIP 5 S-TCT CTC TCC ACC CTT ATC CC	IDT	N/A
Mus musculus AhR-NLRP6 -CHIP 5 AS-AGG TCT TCA GAA ATG GCC TTA	IDT	N/A
Mus musculus AhR-NLRP6 -CHIP 6 S-AAC CAA AGG TCG CAG TCC	IDT	N/A

Mus musculus AhR-NLRP6 -CHIP 6 AS-CAG CTG GAC TCT TGC TGA C	IDT	N/A
**Software and Algorithms**		
Image Lab software	Bio-Rad	http://www.bio-rad.com/en-us/sku/1709690-image-lab-software
GraphPad Prism 10.0	GraphPad Software	http://www.GraphPad.com/
ImageJ	NIH	https://imagej.nih.gov/ij/index.html
FlowJo software	BD Biosciences	https://www.flowjo.com/solutions/flowjo
JASPAR	JASPAR	https://jaspar.elixir.no/

Table S2

Subject information for human endoscopic specimens related to Figure 8.

CD; Crohn's disease. UC; ulcerative colitis

	**Details**
**Sample1**	UC patient
**Sample2**	Normal 62yr+ male
**Sample3**	Pt 1. 20s female with mildly active ulcerative colitis. (sample 1 active)
**Sample4**	Pt 1. 20s female with mildly active ulcerative colitis. (sample 2 not active) [Same pt 1]
**Sample5**	Sample 3. pt 2. Early 30s female with quiescent UC.
**Sample6**	Pt 3. Mid-70s female with quiescent Crohn's disease, no medications
**Sample7**	Sample 1. Female with Crohn's disease of colon. First set at ulceration
**Sample8**	Sample 2 Female from sample 1 with Crohn's disease of colon. Her normal appearing tissue.
**Sample9**	Sample 3. Male patient., CD active
**Sample10**	Sample 4. Female patient with quiescent ulcerative colitis
**Sample11**	Pt 1. 60 female for colon cancer screening. Normal colon. 1A
**Sample12**	Pt 1. 60 female for colon cancer screening. Normal colon. 1B
**Sample13**	Pt 2. 25 male with Acute mild colitis 2
**Sample14**	pt 1 Ulcerative colitis (Remission) 1
**Sample15**	pt 2 UC patient - Normal 2a
**Sample16**	pt 2 UC patient - inflamed 2b
**Sample17**	pt 3 UC patient - Normal 3a
**Sample18**	pt 3 UC patient - inflamed 3b
**Sample19**	Pt 1. 51 yo female with UC on Entyvio. No active inflammation
**Sample20**	Pt 2. 77 yo female with UC on azathioprine. No active inflammation
