# Supplementary material for: Urolithin A activates aryl hydrocarbon receptor-NLRP6-mediated pathways in intestinal epithelial cells to modulate mucosal immunity and strengthen gut barrier integrity
Source: Nat Commun. 2026 Jun 23;17:5411. doi: 10.1038/s41467-026-73760-3 (PMC13291259; doi:10.1038/s41467-026-73760-3)
Supplement: Supplementary file 3 — Reporting Summary [file 41467_2026_73760_MOESM3_ESM.pdf]

nature portfolio

Corresponding author(s): Venkatakrishna Rao Jala

Last updated by author(s): May 11, 2026

Reporting Summary

Nature Portfolio wishes to improve the reproducibility of the work that we publish. This form provides structure for consistency and transparency in reporting. For further information on Nature Portfolio policies, see our Editorial Policies and the Editorial Policy Checklist.

Statistics

For all statistical analyses, confirm that the following items are present in the figure legend, table legend, main text, or Methods section.

n/a	Confirmed	
☐	☒	The exact sample size (*n*) for each experimental group/condition, given as a discrete number and unit of measurement
☐	☒	A statement on whether measurements were taken from distinct samples or whether the same sample was measured repeatedly
☐	☒	The statistical test(s) used AND whether they are one- or two-sided<br>*Only common tests should be described solely by name; describe more complex techniques in the Methods section.*
☒	☐	A description of all covariates tested
☒	☐	A description of any assumptions or corrections, such as tests of normality and adjustment for multiple comparisons
☐	☒	A full description of the statistical parameters including central tendency (e.g. means) or other basic estimates (e.g. regression coefficient) AND variation (e.g. standard deviation) or associated estimates of uncertainty (e.g. confidence intervals)
☐	☒	For null hypothesis testing, the test statistic (e.g. *F*, *t*, *r*) with confidence intervals, effect sizes, degrees of freedom and *P* value noted<br>*Give P values as exact values whenever suitable.*
☒	☐	For Bayesian analysis, information on the choice of priors and Markov chain Monte Carlo settings
☒	☐	For hierarchical and complex designs, identification of the appropriate level for tests and full reporting of outcomes
☒	☐	Estimates of effect sizes (e.g. Cohen's *d*, Pearson's *r*), indicating how they were calculated

Our web collection on statistics for biologists contains articles on many of the points above.

Software and code

Policy information about availability of computer code

Data collection	FACSDiva v8.0, Image Lab software v4.1,
Data analysis	Graphpad Prism 10, FlowJo 11 , JASPER, Image J, Excel

For manuscripts utilizing custom algorithms or software that are central to the research but not yet described in published literature, software must be made available to editors and reviewers. We strongly encourage code deposition in a community repository (e.g. GitHub). See the Nature Portfolio guidelines for submitting code & software for further information.

Data

Policy information about availability of data

All manuscripts must include a data availability statement. This statement should provide the following information, where applicable:

- Accession codes, unique identifiers, or web links for publicly available datasets
- A description of any restrictions on data availability
- For clinical datasets or third party data, please ensure that the statement adheres to our policy

Data will be available to the public.

Research involving human participants, their data, or biological material

Policy information about studies with human participants or human data. See also policy information about sex, gender (identity/presentation), and sexual orientation and race, ethnicity and racism.

Reporting on sex and gender	We used both sex and findings are applicable to both the genders.
Reporting on race, ethnicity, or other socially relevant groupings	Not biased to any race, ethnicity or any relevant grouping
Population characteristics	Not biased
Recruitment	No
Ethics oversight	For human samples, intestinal biopsies from the active inflamed region of individuals with inflammatory bowel disease (IBD) or controls were obtained following Institutional Ethical Review Board approved protocols from University of Louisville Hospital.

Note that full information on the approval of the study protocol must also be provided in the manuscript.

Field-specific reporting

Please select the one below that is the best fit for your research. If you are not sure, read the appropriate sections before making your selection.

☒ Life sciences ☐ Behavioural & social sciences ☐ Ecological, evolutionary & environmental sciences

For a reference copy of the document with all sections, see nature.com/documents/nr-reporting-summary-flat.pdf

Life sciences study design

All studies must disclose on these points even when the disclosure is negative.

Sample size	Sample size was determined based on the experience of the previous in vivo experiments.
Data exclusions	None
Replication	Experiments were repeated at least twice, usually three times, and data pooled from independent experiments to verify consistent results. We also used different knockout mice along with appropriate litter mates to verify the role of Urolithin A.
Randomization	Randomization was not used. Mice were age/sex matched, where possible littermate controls were used. The repeat experiments were at different time points and different cohorts of mice.
Blinding	All data collected in a blinded fashion.

Reporting for specific materials, systems and methods

We require information from authors about some types of materials, experimental systems and methods used in many studies. Here, indicate whether each material, system or method listed is relevant to your study. If you are not sure if a list item applies to your research, read the appropriate section before selecting a response.

Materials & experimental systems

n/a	Involved in the study	
☐	☒	Antibodies
☐	☒	Eukaryotic cell lines
☒	☐	Palaeontology and archaeology
☐	☒	Animals and other organisms
☒	☐	Clinical data
☒	☐	Dual use research of concern
☒	☐	Plants

Methods

n/a	Involved in the study	
☒	☐	ChIP-seq
☐	☒	Flow cytometry
☒	☐	MRI-based neuroimaging

Antibodies

Antibodies used	

Antibodies used

AHR Polyclonal antibody Proteintech Cat# 17840-1-AP 1:1000
IL-18 Polyclonal antibody Proteintech Cat# 10663-1-AP 1:1000
MUC2 Polyclonal antibody Proteintech Cat# 27675-1-AP 1:1000
Reg3G Antibody Novus Biologicals Cat# NBP2-93737 1:1000
NLRP6 antibody Sigma Cat# SAB1302240 1:1000
Goat anti-rabbit IgG (H+L), HRP conjugate Proteintech Cat# SA00001-2 1:5000
HRP-conjugated Beta Actin Monoclonal antibody Proteintech Cat# HRP-60008 1:5000
Goat anti-Rabbit IgG (H+L) Cross-Adsorbed Secondary Antibody, Alexa Fluor™ 488 Invitrogen Cat# A-11008 1:5000
APC/Cyanine7 anti-mouse CD45.2 Antibody Biolegend Cat# 109824 1:100
FITC anti-mouse Lineage Cocktail with Isotype Ctrl Biolegend Cat# 133302 1:100
PerCP/Cyanine5.5 anti-mouse CD335 (NKp46) Antibody Biolegend Cat# 137610 1:100
Alexa Fluor® 647 Mouse Anti-Mouse RORγt BD Pharmingen Cat# 562682 1:100
Mouse IL-22 PE-conjugated Antibody R&D Systems™ Cat# IC582P 1:100
PE anti-mouse IL-22 Antibody Biolegend Cat# 516404 1:100
Alexa Fluor® 647 anti-mouse CD326 (Ep-CAM) Antibody Biolegend Cat# 118212 1:100
FITC Donkey anti-rabbit IgG (minimal x-reactivity) Antibody Biolegend Cat# 406403 1:100
APC/Cyanine7 anti-mouse CD45 Antibody Biolegend Cat# 103115 1:100
PE/Cyanine7 anti-mouse CD11c Antibody Biolegend Cat# 117317 1:100
PE anti-mouse F4/80 Recombinant Antibody Biolegend Cat# 157304 1:100
APC/Cyanine7 anti-human CD45 Antibody Biolegend Cat# 304014 1:100
FITC anti-human Lineage Cocktail (CD3, CD14, CD16, CD19, CD20, CD56) Biolegend Cat# 348801 1:100
PE/Cyanine7 anti-human CD336 (NKp44) Antibody Biolegend Cat# 325115 1:100
PE anti-human CD127 (IL-7Rα) Antibody Biolegend Cat# 351340 1:100
APC anti-human CD117 (c-kit) Antibody Biolegend Cat# 375307 1:100
PerCP/Cyanine5.5 anti-human IL-22 Antibody Biolegend Cat# 366709 1:100
Alexa Fluor® 647 anti-human CD326 (EpCAM) Antibody Biolegend Cat# 369819 1:100
Mouse IL-23 p19 Antibody R&D Systems Cat# AF1619SP 1:100
IL-22 Monoclonal Antibody eBioscience Cat# 16-7222-82 1:100
InVivoMAb anti-mouse/rat IL-1β Bio X Cell Cat# BE0246 1:100
InVivoMAb anti-mouse IL-18 Bio X Cell Cat# BE0237 1:100
BD Pharmingen™ PerCP-Cy™5.5 Mouse Anti-Mouse CD45.2 BD Cat# 552950 1:100
BD Horizon™ BUV395 Rat Anti-Mouse CD45 BD 565967 1:100
BD Horizon™ BV421 Hamster Anti-Mouse CD3e BD Cat# 562600 1:100
BD Horizon™ BV421 Rat Anti-Mouse CD19 BD Cat# 562701 1:100
BD Horizon™ BV421 Rat Anti-Mouse Ly-6G and Ly-6C BD Cat# 562709 1:100
BD Horizon™ BV421 Rat Anti-Mouse F4/80 BD Cat# 565411 1:100
BD Horizon™ BUV395 Mouse Anti-GATA3 BD Cat# 565448 1:100
BD Pharmingen™ PE-Cy™7 Mouse anti-GATA3 BD 560405 1:100
Brilliant Violet 650™ anti-mouse CD127 (IL-7Rα) Antibody Biolegend Cat# 135043 1:100
APC anti-mouse CD196 (CCR6) Antibody Biolegend Cat# 129814 1:100
Brilliant Violet 421™ anti-mouse TCR γ/δ Antibody Biolegend Cat# 118120 1:100
Brilliant Violet 421™ anti-mouse TCR β chain Antibody Biolegend Cat# 109230 1:100
Brilliant Violet 421™ anti-mouse CD5 Antibody Biolegend Cat# 100629 1:100
PE/Cyanine7 anti-mouse TCR γ/δ Antibody Biolegend Cat# 118124 1:100
APC/Cyanine7 anti-mouse CD8a Recombinant Antibody Biolegend Cat# 155016 1:100
ROR gamma (t) Monoclonal Antibody (B2D), Alexa Fluor™ 488, eBioscience™ Thermo Fisher Scientific Cat# 53-6981-82 1:100

BD Pharmingen™ PerCP-Cy™5.5 Mouse Anti-Mouse RORγt BD 562683 1:100
IL-22 Monoclonal Antibody (1H8PWSR), PE, eBioscience™ Thermo Fisher Scientific Cat# 12-7221-82 1:100
EOMES Monoclonal Antibody (Dan11mag), PE-eFluor™ 610, eBioscience™ Thermo Fisher Scientific Cat# 61-4875-82 1:100
BD Pharmingen™ Alexa Fluor™ 488 Mouse Anti-EOMES BD 567169 1:100
KLRG1 Monoclonal Antibody (2F1), APC-eFluor™ 780, eBioscience™ Thermo Fisher Scientific Cat# 47-5893-82 1:100
CD4 Monoclonal Antibody (GK1.5), Alexa Fluor™ 700, eBioscience™ Thermo Fisher Scientific Cat# 56-0041-82 1:100
CD335 (NKp46) Monoclonal Antibody (29A1.4), PE-Cyanine7, eBioscience™ Thermo Fisher Scientific Cat# 25-3351-82 1:100
CD335 (NKp46) Monoclonal Antibody (29A1.4), PE-eFluor™ 610, eBioscience™ Thermo Fisher Scientific 61-3351-82 1:100
CD90.2 (Thy-1.2) Monoclonal Antibody (53-2.1), eFluor™ 506, eBioscience™ Thermo Fisher Scientific 69-0902-82 1:100
LIVE/DEAD™ Fixable Yellow Dead Cell Stain Kit, for 405 nm excitation Thermo Fisher Scientific Cat# L34959 1:100
IL-17A Monoclonal Antibody (eBio17B7), APC, eBioscience™ Thermo Fisher Scientific Cat# 17-7177-81 1:100

Validation

All antibodies are are validated by manufacturer . Lab also evaluated the efficacy and dilution factors.

Eukaryotic cell lines

Policy information about cell lines and Sex and Gender in Research

Cell line source(s)

MNK-3 cell Dr. James Carlyle, University of Toronto, Canada
WT murine intestinal organoids This paper
Ahr-/- murine intestinal organoids This paper
Il18-/- murine intestinal organoids This paper
Nlrp3-/- murine intestinal organoids This paper
Nlrp6-/- murine intestinal organoids This paper

Authentication

MNK-3 cell authentication was done by the inventors and organoids were authenticated by gene expression analysis by lab.

Mycoplasma contamination	No contamination
Commonly misidentified lines (See ICLAC register)	N/A

Animals and other research organisms

Policy information about studies involving animals; ARRIVE guidelines recommended for reporting animal research, and Sex and Gender in Research

Laboratory animals	C57BL/6 WT The Jackson Laboratory Strain# 000664<br>C57BL/6-Ahrtm1.2Arte Taconic Laboratories Model# 9166<br>B6.129P2-Il18tm1Aki/J The Jackson Laboratory Strain# 004130<br>Ahrtm3.1Bra/J The Jackson Laboratory Strain# 006203<br>B6.Cg-Tg(Vil1-cre)997Gum/J The Jackson Laboratory Strain# 004586<br>B6.129P2-Lyz2tm1(cre)Ifo/J The Jackson Laboratory Strain# 004781<br>Ahrfx-VillinCre This paper N/A<br>Ahrfx-LysMCre This paper N/A<br>Nlrp6fx/fx gift from Dr. Daniel Mucida, Rockefeller University (Cell 180, 64-78.e16 (2020))<br>Nlrp6fx-VillinCre This paper N/A<br><br>Ill-22-/- Gift from Dr. Misty Good , University of North Carolina (Infect Immun 87, (2019); Cell Reports Medicine 2, 100320 (2021))<br>Il22Ra1fx Dr. Pawan Kumar (Stony Brook University N/A (Infect Immun 84, 782-789 (2016); Nature Communications 15, 1597 (2024))<br>Il22Ra1fx-VillinCre This paper N/A
Wild animals	N/A
Reporting on sex	Study involves both the sex
Field-collected samples	N/A
Ethics oversight	All the experimental studies were performed under approved protocols from Institutional Animal Care and Use Committee (IACUC), University of Louisville, Louisville, KY, USA and adhered to guidelines in the Guide for the Care and Use of Laboratory Animals of the National Institutes of Health (NIH).

Note that full information on the approval of the study protocol must also be provided in the manuscript.

Plants

Seed stocks	n/a
Novel plant genotypes	n/a
Authentication	n/a

Flow Cytometry

Plots

Confirm that:

☒ The axis labels state the marker and fluorochrome used (e.g. CD4-FITC).

☒ The axis scales are clearly visible. Include numbers along axes only for bottom left plot of group (a 'group' is an analysis of identical markers).

☒ All plots are contour plots with outliers or pseudocolor plots.

☒ A numerical value for number of cells or percentage (with statistics) is provided.

Methodology

Sample preparation	Cells from mice lamina propria lymphocytes were isolated using mouse Lamina Propria Dissociation Kit (Miltenyi biotec, USA) following manufacture's protocol with help of gentleMACS™ Octo Dissociator with Heaters (Miltenyi biotec, USA). Cells were further isolated by Percoll gradient described.

For flowcytometric analysis of organoids and organoid-lamina propria cell coculture, organoids were collected and washed with cold medium and centrifuged at 200g for 5 min followed by resuspension in 1 mL of pre-warmed 1X TrypLE™ Express Enzyme (Thermofisher). Organoids were pipetted using a narrowed Pasteur pipette for10 times and incubated for 5 min at 37 °C to make single cells. After incubation, the cells were pipetted 10 times and checked for single cells. Digestion was stopped by adding cold medium and the digest was filtered through a 40 µm pore nylon cell strainer (Falcon) to remove doublets and further processed for flowcytometric analysis.
Single-cell suspensions from ihuman ntestinal tissues were obtained by incubating tissues for 30 min at 37 °C with shaking in stripping buffer (1 mM EDTA, 1 mM DTT and 5% FBS) to separate the epithelial layer. The supernatants containing epithelial layer were kept separately, and rest tissues were then mechanically dissociated with a sterile scalpel. The lamina propria fraction from the tissue was obtained by incubating the dissociated tissues for 1 h at 37 °C with shaking in 2 mg/ml collagenase D (Roche), 0.1 mg/ml DNase I (Sigma Aldrich) and 1 mg/ml of trypsin inhibitor (Gibco) digestion solution. Further, the remaining tissues were then filtered through a 70-µm cell strainer and cells were further combined with epithelial layer fraction to analyze as the whole tissue single cells.

Instrument

BD LSRFortessa

Software

FACSDiva, FlowJo

Cell population abundance

No sorting experiments were performed.

Gating strategy

Gating strategy was determined based on prior literature. We used all appropriate Fluorescence Minus One (FMO) controls, Knock mouse cells controls, positive controls in our flow experiments.

☒ Tick this box to confirm that a figure exemplifying the gating strategy is provided in the Supplementary Information.
