# Supplementary material for: Urolithin A activates aryl hydrocarbon receptor-NLRP6-mediated pathways in intestinal epithelial cells to modulate mucosal immunity and strengthen gut barrier integrity
Source: Nat Commun. 2026 Jun 23;17:5411. doi: 10.1038/s41467-026-73760-3 (PMC13291259; doi:10.1038/s41467-026-73760-3)
Supplement: Supplementary file 4 — Source Data [file 41467_2026_73760_MOESM4_ESM.zip › Source data/Full western blot images.pdf]

Original full Western blots:

All the antibodies were tested against mouse colon tissue extracts. 40 μg of the protein was loaded into the wells. The full gel – Western blots are shown below. In some of the experiments, the transferred membranes were cut according to their molecular weights and probed with indicated respective antibodies. Molecular weights are represented as kDa (kilodaltons)

Figure 4G and H – NLRP6
β actin

Figure 5A– NLRP6
β actin

Figure 6A and B– MUC2
Reg3γ
β actin

Supplementary Figure 1B- AHR
β actin

Supplementary Figure 10C and 10D – IL-18
β actin

4G and 4H

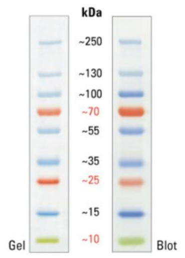

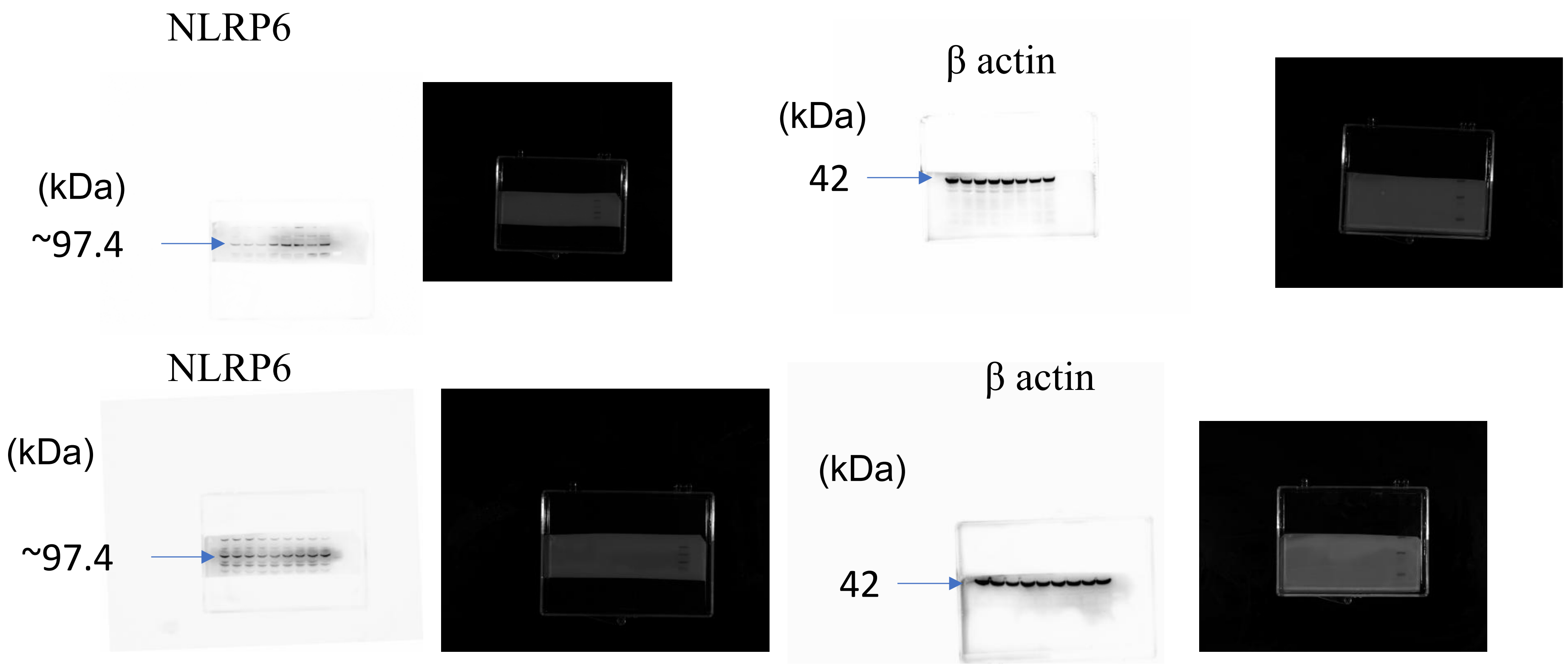

Molecular weights are represented as kDa (kilodaltons)

Figure 5A

NLRP6

(kDa)

~97.4

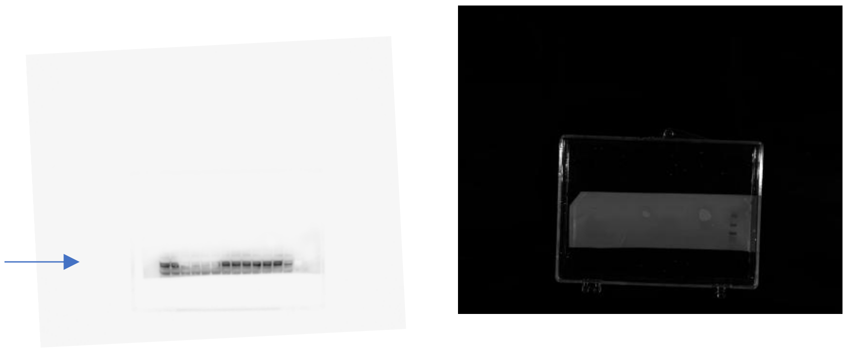

β actin

(kDa)

42

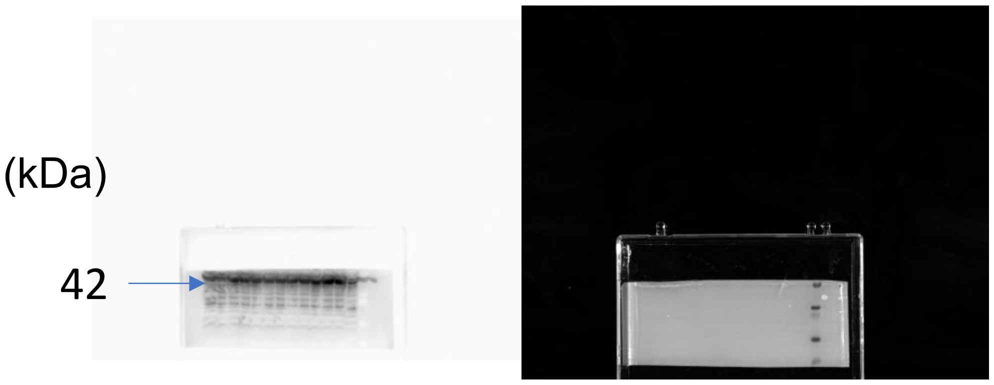

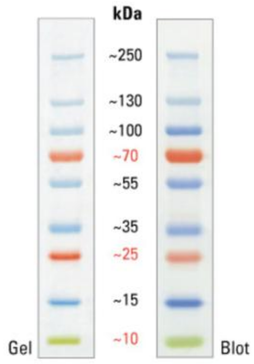

Figure 6A

MUC2

(kDa)

110

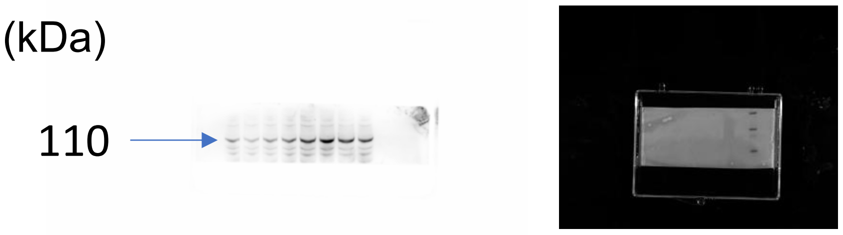

REG3γ

(kDa)

19

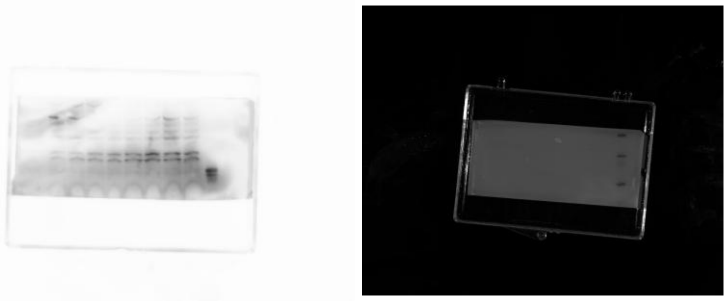

β actin

(kDa)

42

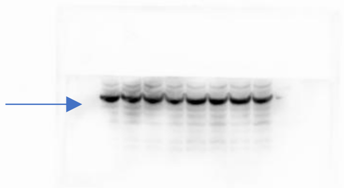

Figure 6B

MUC2

(kDa)

110

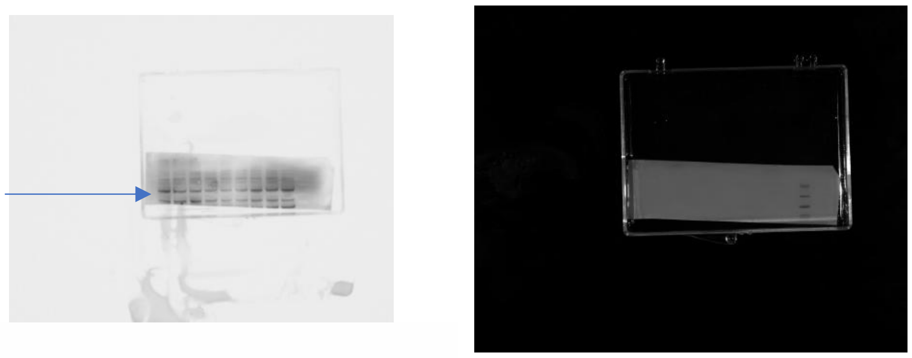

REG3γ

(kDa)

19

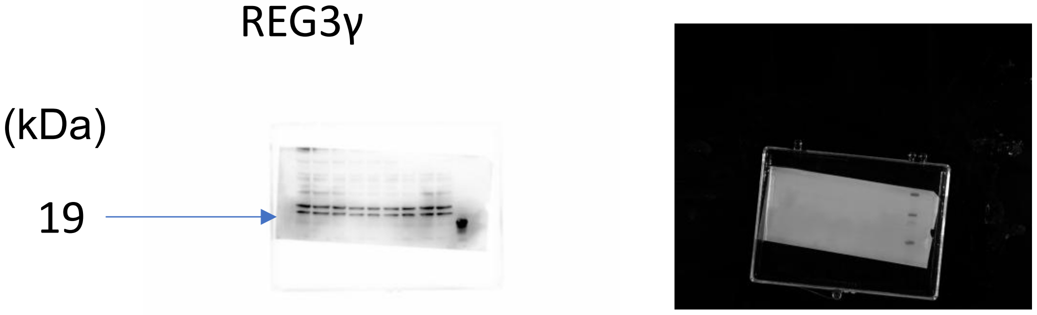

β actin

(kDa)

42

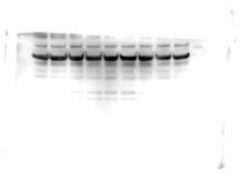

Molecular weights are represented as kDa (kilodaltons)

Figure S1B

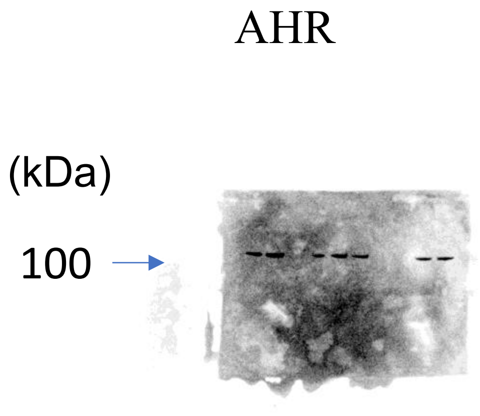

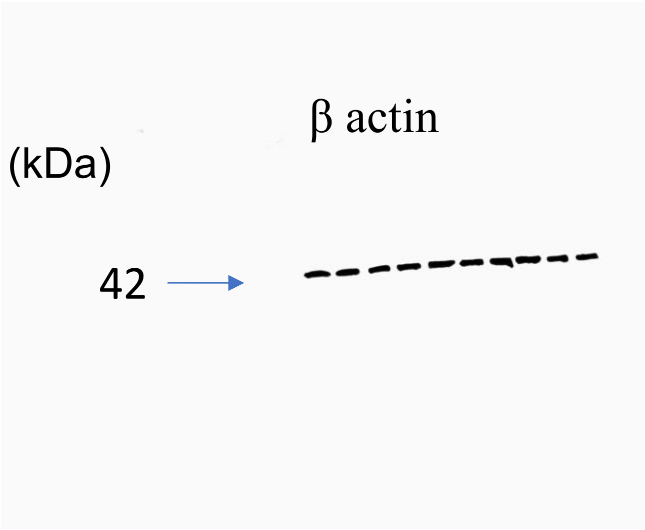

Molecular weights are represented as kDa (kilodaltons)

Figure S10 C

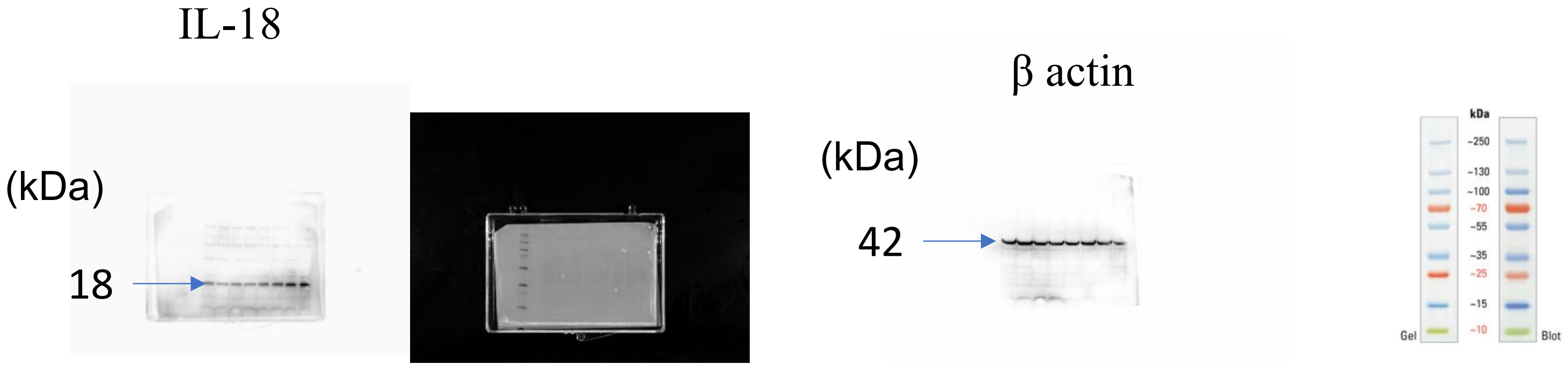

Figure S10 D

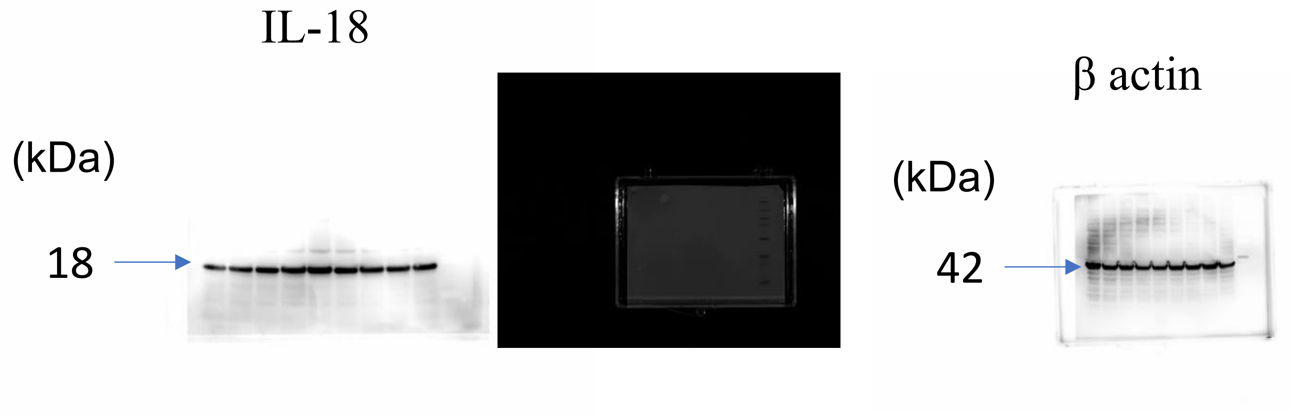

Molecular weights are represented as kDa (kilodaltons)
