# Supplementary material for: Urolithin A activates aryl hydrocarbon receptor-NLRP6-mediated pathways in intestinal epithelial cells to modulate mucosal immunity and strengthen gut barrier integrity
Source: Nat Commun. 2026 Jun 23;17:5411. doi: 10.1038/s41467-026-73760-3 (PMC13291259; doi:10.1038/s41467-026-73760-3)

nature portfolio

Peer Review File

Urolithin A activates aryl hydrocarbon receptor-NLRP6-mediated pathways in intestinal epithelial cells to modulate mucosal immunity and strengthen gut barrier integrity

Corresponding Author: Dr Venkatakrishna Jala

This file contains all reviewer reports in order by version, followed by all author rebuttals in order by version. Parts of this Peer Review File have been redacted as indicated to maintain the confidentiality of unpublished data.

Version 0:

Reviewer comments:

Reviewer #1

(Remarks to the Author)
In the current report by Ghosh et al, studies were conducted to assess underlying mechanisms of urolithin A (UroA) in protection against murine models of colitis (mainly DSS, but also infection model). A current literature search for UroA and IBD research shows only 8 hits, including previous publication from the research team (PMID 39533380). Therefore, this work on the effects of UroA in IBD and related research is still quite novel, suggesting need for further investigation. In addition, this study is interesting in that it highlights a potential well-described and defined mechanistic pathway, specifically through AhR-NLRP6-IL18 in intestinal epithelial cells (e.g. UroA increases IL-18 in IECs that promote IL-22 production in ILC3s). These findings are interesting due to the independent nature from other factors (e.g. AhR activation in other immune cells) or cell types (e.g. macrophages, other IL-22 producers), which was well-characterized in the current report with the use of various controls and genetic KOs. The research design was sophisticated and comprehensive, leading to the results being very convincing and exciting. Overall, this is a well-designed and executed study, with significant and important findings that impact areas of mucosal immunology and IBD research.

Some minor to moderate concerns that could be addressed include the following:

1. The study states that both sexes/genders were used, but it was not clear in the methods/figure legends/supplementary material how this was distributed. What was the ratio of male to female? In particular, DSS colitis is more severe in males than females. Was this taken into consideration or does this impact pooling together data as was done. In addition, AhR is know to have sex differences, therefore, it should be stated clearly if the results in the current study are in fact representative of both sexes equally, or if more dominant (e.g. male bias).

2. In the results section (page 12), it states that Figure 3F-G show both WT and AhR-/- organoids. It does not appear that AhR-/- are shown. Was this stated in error?

3. The manuscript could use additional proofing. There were several instances of grammatical mistakes. Examples of this include:
-Abbreviations are not correctly used. There were instances of an abbreviation being established early in the paper and then repeated writing out and showing abbreviation in parentheses. Suggested to correct (e.g. IBD and DSS are written out and abbreviated numerous times in text; eg. page 20)
-It should be "flow cytometry", not "flowcytometry" (e.g. as one word). This should be corrected. Examples include page 11 second line,

4. References cited in the text do not conform with journal guidelines, particularly with how they should be numbered (e.g. page 7; instead of 10,19, 20, 21, 22, 23, 24, 25, 26 - should be 10, 19-26) - suggest correcting

Reviewer #2

(Remarks to the Author)

It has been reported by this group previously that UroA protects mice from gut inflammation in a Ahr-dependent manner. The manuscript by Ghosh et al is trying to identify the downstream cellular and molecular mechanisms of UroA-mediated protection. By depleting Ahr in epithelial cells or macrophages, the authors find that epithelial-Ahr is essential. Next, the authors use in vitro organoid-immune cell co-culture assay to determine the molecular mechanism, and identified that NLRP6-IL-18-IL-22 can be trigger by UroA. However, further in vivo mechanistic analyses are required, as some of the in vivo findings have been controversial.

Major points:
1. ILC3s decreases significantly in inflamed tissue. In Fig 2, the observation that IL-22+ ILC3s decreases in Ahr-/- or Villin-Cre Ahr-floxed mice could be due to more severe gut inflammation in these mice which leads to ILC3 loss. Further in vivo evidence is needed to show that UroA mediates IL-22 production in ILC3s. Does UroA treatment in naïve mice induce IL-22+ ILC3s and IL-22 production?
2. The authors claim that epithelial-Ahr is the downstream signal of UroA to produce IL-22. However, previous report (PMID 30119997) and Fig 2 of this manuscript showed that loss of epithelial-Ahr has no impact on ILC3 and IL-22 production during inflammation. Could the author explain this?
3. Ahr signaling regulates epithelial stem cell differentiation. If UroA activate Ahr signaling in gut epithelial cells, does it impact epithelial stem cell differentiation, or repair after injury?
4. In Fig 3, could the authors show IL22 production in ILC3s with or without anti-IL-18 treatment?
5. The authors suggest that Nlrp6 is responsible for IL-18 production in epithelial cells which is the trigger of IL-22 to protect the gut. Fig 5 shows that there is an IL-18 increase in the colon tissue in Villin-Cre Nlrp6-floxed mice after DSS but no IL-22 increase. Instead, in Figure S14, UroA decreased IL-18 in Nlrp6-floxed mice and IL-22 is increased. These in vivo data suggests that Epithelial-Nlrp6 is not essential for IL-18 production and IL-18 level does not corelates with IL-22 production after DSS. Could the author explain this?

Minor points:
1. Fig 1b, statistically analysis is needed (also for all body weight quantifications). Please consider to change the colors of each group, especially for groups with similar kinetics. It is difficult to differentiate one from the other with the current color.
2. The immune cell viability should be reported for all co-culture assays and the human samples after 24 hour treatment, as usually the viability of long-time cultured primary cells is very low, making it difficult to interpret downstream functional readout.

Reviewer #3

(Remarks to the Author)
This is a well written paper, describing a series of well controlled experiments that, collectively, have mechanistically advanced understanding of Urolithin A, a food borne microbiota generated flavonoid ameliorate colitis. Specifically, they show that Uro A, but not 2 other Ahr ligands drive an Ahr-dependent, activation of NLRP6 that results in IL-18 production that activates ILC3-mediated IL-22, which is well known to protect against acute colitis. This is a solid advance towards understanding the bioactions of this important molecule. I have but a couple of minor comments.

1. The figure legends are too long and highly repetitive. It is not necessary for every figure to note use of GraphPad Prism. This and many other statements in legends should be moved to Methods.

2. Some of the statements re Uro A eliciting specific responses (not shared by other Ahr ligands) are a bit strong. They clearly showed this to be true for IL-18 but they use the term select pretty liberally.

Version 1:

Reviewer comments:

Reviewer #1

(Remarks to the Author)
In the current report by Ghosh et al (revision), studies were conducted to assess underlying mechanisms of urolithinA (UroA) in protection against murine models of colitis (mainly DSS, but also infection model). UroA and IBD research remains novel, including previous publication from the research team (PMID 39533380). The report includes a number of techniques and models to focus on the AhR-NLRP6-IL18 pathway in intestinal epithelial cells (e.g. UroA increases IL-18 in IECs that promote IL-22 production inILC3s). Overall, this is a well-designed and executed study, with significant and important findings that impact areas of mucosal immunology and IBD research.

For the revision, authors addressed all issues raised in detail in the rebuttal letter. In addition, changes/modifications to address issues raised were highlighted/clearly defined in revision. The report has been improved overall and still remains significant to the research field. The methods are detailed and the conclusions claims are for the most part justified by the data and results, or otherwise noted in the discussion.

Reviewer #2

(Remarks to the Author)
The authors addressed all of my comments sufficiently.

Reviewer #3

(Remarks to the Author)
My minor concerns have been alleviated resulting in a high-quality report.

Open Access This Peer Review File is licensed under a Creative Commons Attribution 4.0 International License, which permits use, sharing, adaptation, distribution and reproduction in any medium or format, as long as you give appropriate credit to the original author(s) and the source, provide a link to the Creative Commons license, and indicate if changes were made.
In cases where reviewers are anonymous, credit should be given to 'Anonymous Referee' and the source.
The images or other third party material in this Peer Review File are included in the article's Creative Commons license, unless indicated otherwise in a credit line to the material. If material is not included in the article's Creative Commons license and your intended use is not permitted by statutory regulation or exceeds the permitted use, you will need to obtain permission directly from the copyright holder.
To view a copy of this license, visit https://creativecommons.org/licenses/by/4.0/

RESPONSE TO REVIEWERS' COMMENTS

Reviewer #1 (Remarks to the Author):

In the current report by Ghosh et al, studies were conducted to assess underlying mechanisms of urolithin A (UroA) in protection against murine models of colitis (mainly DSS, but also infection model). A current literature search for UroA and IBD research shows only 8 hits, including previous publication from the research team (PMID 39533380). Therefore, this work on the effects of UroA in IBD and related research is still quite novel, suggesting need for further investigation. In addition, this study is interesting in that it highlights a potential well-described and defined mechanistic pathway, specifically through AhR-NLRP6-IL18 in intestinal epithelial cells (e.g. UroA increases IL-18 in IECs that promote IL-22 production in ILC3s). These findings are interesting due to the independent nature from other factors (e.g. AhR activation in other immune cells) or cell types (e.g. macrophages, other IL-22 producers), which was well-characterized in the current report with the use of various controls and genetic KOs. The research design was sophisticated and comprehensive, leading to the results being very convincing and exciting. Overall, this is a well-designed and executed study, with significant and important findings that impact areas of mucosal immunology and IBD research.

Some minor to moderate concerns that could be addressed include the following:

1. The study states that both sexes/genders were used, but it was not clear in the methods/figure legends/supplementary material how this was distributed. What was the ratio of male to female? In particular, DSS colitis is more severe in males than females. Was this taken into consideration or does this impact pooling together data as was done. In addition, AhR is know to have sex differences, therefore, it should be stated clearly if the results in the current study are in fact representative of both sexes equally, or if more dominant (e.g. male bias).

Response: We are grateful to the reviewer for highlighting this important point. We have now clarified this in the Materials and Methods section of the revised manuscript. For each genotype, we aimed to maintain an approximately equal ratio of male and female mice within each experimental group whenever possible. Additional details are provided in the Methods section and corresponding figure legends wherever necessary. Importantly, in our facility, 2.5% DSS caused only minor sex-specific differences in disease severity, and Urolithin A provided comparable protection in both males and females. Therefore, we pooled the data, and the results presented represent both sexes

2. In the results section (page 12), it states that Figure 3F-G shows both WT and AhR-/- organoids. It does not appear that AhR-/- are shown. Was this stated in error?

Response: We thank the reviewer for carefully examining the figures and bringing this issue to our attention. We apologize for the oversight. The *Ahr*$^{-/-}$ reference has now been removed from results section and from the corresponding figure legends in the revised manuscript.

3. The manuscript could use additional proofing. There were several instances of grammatical mistakes. Examples of this include:

-Abbreviations are not correctly used. There were instances of an abbreviation being established early in the paper and then repeated writing out and showing abbreviation in parentheses. Suggested to correct (e.g. IBD and DSS are written out and abbreviated numerous times in text; eg. page 20)

-It should be "flow cytometry", not "flowcytometry" (e.g. as one word). This should be corrected. Examples include page 11 second line,

Response: We thank the reviewer for this helpful comment and apologize for the grammatical and typographical errors in the original submission. We have corrected the mistakes, and the manuscript has now been thoroughly proofread and revised.

4. References cited in the text do not conform with journal guidelines, particularly with how they should be numbered (e.g. page 7; instead of 10,19, 20, 21, 22, 23, 24, 25, 26 - should be 10, 19-26) - suggest correcting

Response: We have corrected the reference citations according to journal's guidelines.

We appreciate the reviewer's careful reading and all insightful comments, which has helped improve the accuracy of the manuscript.

Reviewer #2 (Remarks to the Author):

It has been reported by this group previously that UroA protects mice from gut inflammation in a Ahr-dependent manner. The manuscript by Ghosh et al is trying to identify the downstream cellular and molecular mechanisms of UroA-mediated protection. By depleting Ahr in epithelial cells or macrophages, the authors find that epithelial-Ahr is essential. Next, the authors use in vitro organoid-immune cell co-culture assay to determine the molecular mechanism, and identified that NLRP6-IL-18-IL-22 can be trigger by UroA. However, further in vivo mechanistic analyses are required, as some of the in vivo findings have been controversial.

Major points:

1. ILC3s decreases significantly in inflamed tissue. In Fig 2, the observation that IL-22+ ILC3s decreases in Ahr-/- or Villin-Cre Ahr-floxed mice could be due to more severe gut inflammation in these mice which leads to ILC3 loss. Further in vivo evidence is needed to show that UroA mediates IL-22 production in ILC3s. Does UroA treatment in naïve mice induce IL-22+ ILC3s and IL-22 production?

Response: We thank the reviewer for raising this important point. We acknowledge that the loss of IL-22^{+} ILC3s and reduced IL-22 production in DSS-induced colitis could be due to increased inflammation. As suggested, we evaluated IL-22^{+} ILC3s and IL-22 production in colons of naïve mice following UroA treatment in WT, *Ahr*$^{-/-}$, *Ahr*$^{fx/fx}$ and *Ahr*^{fx}*Villin*^{Cre} mice. Our analysis showed that UroA treatment increased IL-22 production in naïve WT and *Ahr*$^{fx/fx}$ mice, but not in *Ahr*$^{-/-}$ and *Ahr*^{fx}*Villin*^{Cre} mice. These findings have been included in Supplementary Figure S6 of the revised manuscript. This additional dataset suggest that UroA promotes IL-22 production in ILC3s in an epithelial AHR-dependent manner. The figure legend and corresponding text have been updated accordingly for clarity.

2. The authors claim that epithelial-Ahr is the downstream signal of UroA to produce IL-22. However, previous report (PMID 30119997) and Fig 2 of this manuscript showed that loss of epithelial-Ahr has no impact on ILC3 and IL-22 production during inflammation. Could the author explain this?

Response: We thank the reviewer for bringing up this point. We agree that IEC-AHR may not directly regulate IL-22 production by ILC3s during inflammation, consistent with the findings of Metidji et al (PMID 30119997). One possible explanation is that inflammatory cytokines such as IL-1β, IL-23, and IL-18 produced by immune cells (e.g., macrophages and dendritic cells) during inflammatory period can directly stimulate ILC3s to produce IL-22 in an IEC-AHR–independent manner. We postulate that during the recovery phase (day 8-12), UroA-induced IL-22 production requires intestinal epithelial AHR.

To provide context, Zhang et al. (International Journal of Molecular Medicine, 43, 1657–1668, 2019; PMID: 30816423) examined IL-22 expression at multiple time points in an acute DSS-induced colitis model. In their study, mice received 2.5% (w/v) DSS from day 0 to day 5, after which normal drinking water was restored. Animals were sacrificed on days 0, 2, 4, 6, 8, 10, and 12, and intestinal tissues

were collected for RNA and histological analyses. IL-22 expression increased during the inflammatory phase (up to approximately day 6) and declined during the recovery phase (by day 12).

In our study, mice were exposed to DSS for 7 days, followed by a 5-day recovery period. IL-22 levels were assessed on day 12, at a stage when IL-22 expression in vehicle-treated DSS mice is expected to have returned to near-baseline or slightly reduced levels, consistent with the findings of Zhang et al. Notably, UroA treatment resulted in an increased frequency of IL-22-producing ILC3s and reduced inflammatory cytokines at this same time point. This enhancement aligns with improved epithelial barrier function, supported by increased expression of MUC2 and Reg3γ.

3. Ahr signaling regulates epithelial stem cell differentiation. If UroA activate Ahr signaling in gut epithelial cells, does it impact epithelial stem cell differentiation, or repair after injury?

Response: We appreciate the reviewer's suggestion to investigate UroA's potential role in epithelial stem cell differentiation. We conducted preliminary experiments to evaluate whether UroA activates Ahr signaling in gut epithelial cells to have an impact on epithelial stem cell differentiation. The pilot data is provided for the reviewer's reference, and a separate manuscript is under preparation to include complete story. Additional studies are ongoing in our laboratory to further define the role of UroA in regulating epithelial stem cell differentiation and injury-induced regeneration; however, these investigations are beyond the scope of the current manuscript.

UroA enhances the proliferation of intestinal stem cells. As a microbial metabolite produced in close proximity to the gut epithelium, we hypothesized that UroA may regulate intestinal stem cell activity and epithelial regeneration. To test this, we cultured intestinal organoids with UroA or vehicle for three days. As shown in **Fig. 1A–D,** UroA treatment increased the number, size, and proliferation of wild-type organoids (EdU^+ cells). In contrast, organoids derived from $Ahr^{-/-}$ mice did not respond to UroA, indicating that its effects are

REDACTED

AHR-dependent. We further examined stem cell proliferation using organoids prepared from $Lgr5^+$-GFP mice. As shown in **Fig. 1E–G**, UroA treatment increased the proportion of $Lgr5^+$-GFP cells compared with vehicle-treated controls. Together, these findings indicate that UroA promotes the proliferation of intestinal stem cells in an AHR-dependent manner.

4. In Fig 3, could the authors show IL22 production in ILC3s with or without anti-IL-18 treatment?

Response: We thank the reviewer for the suggestion. We have added the IL-22 production analysis in ILC3s with and without anti-IL-18 treatment in Figure 3J. This includes flow cytometry analysis from both coculture experiments and only LP cells from WT mice. The induction of IL-22 from ILC3 by UroA was significantly reduced cocultures treated with an IL-18 neutralizing antibody. In only LP cells, effect of UroA with or without anti-IL-18 treatment has no significance. As observed in LP cells alone, UroA treatment does not induce significant IL-22 production by itself, and therefore we did not perform additional knockout experiments in this context. The findings resemble the corresponding coculture data for RNA expression and ELISA of IL-22 in Figure 3I and 3K. The Figures and legends have been updated accordingly in revised manuscript.

5. The authors suggest that Nlrp6 is responsible for IL-18 production in epithelial cells which is the trigger of IL-22 to protect the gut. Fig 5 shows that there is an IL-18 increase in the colon tissue in Villin-Cre Nlrp6-floxed mice after DSS but no IL-22 increase. Instead, in Figure S14, UroA decreased IL-18 in Nlrp6-floxed mice and IL-22 is increased. These in vivo data suggests that Epithelial-Nlrp6 is not essential for IL-18 production and IL-18 level does not corelates with IL-22 production after DSS. Could the author explain this?

Response:

We appreciate the reviewer's comment. Based on our intestinal organoid and LP co-culture studies, along with cell-specific knockout mouse experiments, we propose that UroA induces IL-18 in intestinal epithelial cells (IECs) in an NLRP6-dependent manner, which may in turn promote IL-22 production by ILC3s. The increased IL-18 observed in DSS-treated colons of $Nlrp6^{fx}$-$Villin^{Cre+}$ *and* $Nlrp6^{fx/fx}$ mice (Figure 5I and S16H) aligns with prior findings showing that IL-18 levels rise sharply during DSS-induced colitis, largely due to infiltrating immune cells producing IL-18 through NLRP3-dependent pathways.

The following text is added to the discussion to explain the observations.

'Interestingly, our data that despite elevated levels of IL-18 in colons of $Nlrp6^{fx}$-$Villin^{Cre+}$ *and* $Nlrp6^{fx/fx}$ mice subjected to DSS, IL-22 remained low at day 12 during recovery in vehicle-treated animals. It is possible that increased inflammatory IL-18 seen in DSS+vehicle-treated mice likely originates from infiltrating immune cells through NLRP3-dependent pathways[92], which may drive goblet cell depletion and hinder epithelial barrier recovery/repair[93] and reduce IL-22. However, UroA treatment reduced inflammatory IL-18 and increased IL-22 during recovery in $Nlrp6^{fx/fx}$ mice, but not in $Nlrp6^{fx}$-$Villin^{Cre+}$.

UroA appears to suppress immune-cell–derived inflammatory IL-18 in NLRP3-dependent manner while promoting NLRP6-dependent IL-18 from IEC. Previous studies support this idea, showing that UroA treatment reduces LPS-induced inflammatory cytokines in BMDMs[22] and inhibits NLRP3 inflammasome activation in LPS-primed BV2 cells[94,95]. Furthermore, unpublished data (**Reviewer only Figure 2)** indicate that UroA treatment decreases LPS+ATP-induced IL-18 and IL-1β in BMDMs in an AHR-dependent manner. Together, these findings suggest that the high IL-18 observed in DSS-treated colons potentially reflects NLRP3-driven inflammation in immune cells, whereas UroA promotes NLRP6-dependent IL-18 in IECs, while suppressing NLRP3-mediated IL-18 in immune cells. This dual action positions UroA as a selective metabolite that enhances IL-18/IL-22–mediated epithelial repair. Future studies are warranted to distinguish the roles of epithelial- versus immune-cell–derived IL-18 across inflammation and recovery using cell-specific IL-18 knockout models. It is also important to measure IL-22 levels at multiple time points (during DSS exposure and recovery) and correlate them with IL-18, other cytokines (such as IL-1β and IL-23), and the extent of tissue damage and recovery in both vehicle- and UroA-treated groups'.

REDACTED

Our pilot studies (**unpublished-Reviewer only Fig. 3**) also show that UroA does not protect $Il18^{fx}$-$Villin^{Cre+}$ mice from DSS-induced colitis, indicating that IEC-derived IL-18 is essential for UroA-mediated protection (based on recovery of colon length). Currently, we are characterizing the phenotype and it will be reported separately. Additional studies are needed to distinguish the roles of epithelial-derived versus immune-cell–derived IL-18 during inflammation and steady-state conditions, using both wild-type mice and cell-specific IL-18 knockout models across disease and recovery time points. It is also important to measure IL-22 levels at multiple time points and correlate them with IL-18, other cytokines (such as IL-1β and IL-23), and the extent of tissue damage and recovery in both vehicle- and UroA-treated groups. These will be part of our future studies.

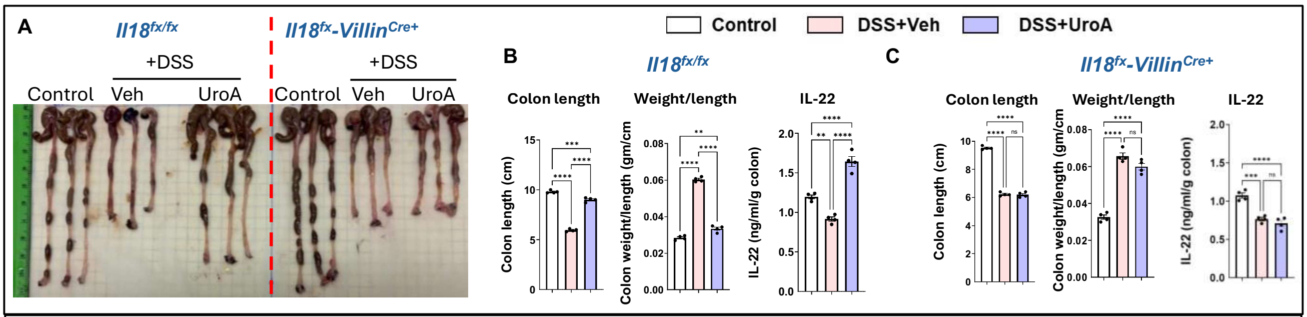

Reviewer only Fig. 3: Intestinal epithelial IL-18 is required for UroA-mediated protective activities against DSS-induced colitis. *Il-18*$^{fx/fx}$ and *Il-18*^{fx}*-Villin*^{Cre} mice (n=5-7, 6–8-week age old) were either on normal water (control group) or subjected to 2.5% DSS in drinking water for 7 days followed by 5 days of regular water. Both control and DSS-subjected mice were treated with either vehicle (1% CMC+0.1% Tween 80) or UroA (20 mg/kg) orally every 48 h of intervals. (A) Gross images of colons are represented. (B) Colon lengths, colon ration of weight/length and colon tissue levels of -22 of *Il-18*$^{fx/fx}$ mice are shown. (C) Colon lengths, colon ration of weight/length and colon tissue levels of -22 of *Il-18*^{fx}*- Villin*^{Cre} mice are shown. Statistics were performed using one-way ANOVA. Error bars, ±SEM; ns: Not significant, **p<0.01; ***p<0.001, ****p<0.0001.

Minor points:

1. Fig 1b, statistically analysis is needed (also for all body weight quantifications). Please consider to change the colors of each group, especially for groups with similar kinetics. It is difficult to differentiate one from the other with the current color.

Response: We agree with reviewer and we have provided the statistical analysis and revised the color scheme for all groups to improve clarity. We have also provided the induvial genotype graphs in Supplementary Figure S2 for better understanding.

2. The immune cell viability should be reported for all co-culture assays and the human samples after 24 hour treatment, as usually the viability of long-time cultured primary cells is very low, making it difficult to interpret downstream functional readout.

Response: Thank you for this important comment. Immune cell viability was rigorously assessed in all co-culture experiments and human primary samples after 24-hour treatment using either trypan blue exclusion or flow cytometry–based live/dead staining. Viability remained high (> 90%) and comparable across all conditions, indicating that the functional readouts are not influenced by differential cell death. These assessments are described in the Materials and Methods section.

Reviewer #3 (Remarks to the Author):

This is a well written paper, describing a series of well controlled experiments that, collectively, have mechanistically advanced understanding of Urolithin A, a food borne microbiota generated flavonoid ameliorate colitis. Specifically, they show that Uro A, but not 2 other Ahr ligands drive an Ahr-dependent, activation of NLRP6 that results in IL-18 production that activates ILC3-mediated IL-22, which is well known to protect against acute colitis. This is a solid advance towards understanding the bioactions of this important molecule. I have but a couple of minor comments.

1. The figure legends are too long and highly repetitive. It is not necessary for every figure to note use of GraphPad Prism. This and many other statements in legends should be moved to Methods. **Response:** We thank the reviewer for these helpful suggestions. We have revised all figure legends to make them more concise and removed repetitive information, including references to GraphPad Prism and other methodological details. These details have now been moved to the Methods section for clarity and consistency.

2. Some of the statements re Uro A eliciting specific responses (not shared by other Ahr ligands) are a bit strong. They clearly showed this to be true for IL-18 but they use the term select pretty liberally. **Response:** We thank the reviewer for this important comment. While our data suggests a specific effect of UroA on IL-18, we have revised the text to moderate statements regarding other responses, specifying where effects are clearly supported by the data and avoiding overgeneralization about selectivity relative to other AhR ligands. We have removed ‘selective activation’ in several places.

We sincerely thank reviewers for their valuable suggestions to strengthen the quality of the manuscript. We are pleased to learn about their approval for publication.

RESPONSE TO REVIEWERS' COMMENTS

Reviewer #1 (Remarks to the Author):
In the current report by Ghosh et al (revision), studies were conducted to assess underlying mechanisms of urolithinA (UroA) in protection against murine models of colitis (mainly DSS, but also infection model). UroA and IBD research remains novel, including previous publication from the research team (PMID 39533380). The report includes a number of techniques and models to focus on the AhR-NLRP6-IL18 pathway in intestinal epithelial cells (e.g. UroA increases IL-18 in IECs that promote IL-22 production inILC3s). Overall, this is a well-designed and executed study, with significant and important findings that impact areas of mucosal immunology and IBD research.
For the revision, authors addressed all issues raised in detail in the rebuttal letter. In addition, changes/modifications to address issues raised were highlighted/clearly defined in revision. The report has been improved overall and still remains significant to the research field. The methods are detailed and the conclusions claims are for the most part justified by the data and results, or otherwise noted in the discussion.
Response: We truly appreciate kind words and thank you for valuable suggestions to improve our MS.

Reviewer #2 (Remarks to the Author):
The authors addressed all of my comments sufficiently.
Response: We truly appreciate kind words and thank you for valuable suggestions to improve our MS.

Reviewer #3 (Remarks to the Author):
My minor concerns have been alleviated resulting in a high-quality report.
Response: We truly appreciate kind words and thank you for valuable suggestions to improve our MS.

Response to Editor suggestions:

We sincerely thank the Editor for the continued support throughout the review process of this manuscript and for the valuable feedback that has significantly strengthened its quality.

We greatly appreciate this constructive and rewarding experience. All suggestions have been carefully addressed, and the required documentation has been provided.

We would also like to inform you that, during the review period of this manuscript, our group published a study confirming AHR expression patterns across different tissues in *Ahr*$^{fx/}$*Villin*$^{Cre+}$ mice using Western blot analysis (PMID: 41635923; *Frontiers in Pharmacology*, January 19, 2026; 16:1706111). In that study, these mice were subjected to alcohol-induced liver disease and no-overlap with current study. Accordingly, this reference has now been incorporated for mouse genotype characterization into the current manuscript. To avoid redundancy, the corresponding confirmatory Western blot previously included in Supplementary Figure S1B of this *Nature Communications* submission has been removed. For completeness and transparency, we are providing the full blot here for your reference.

Figure S1B

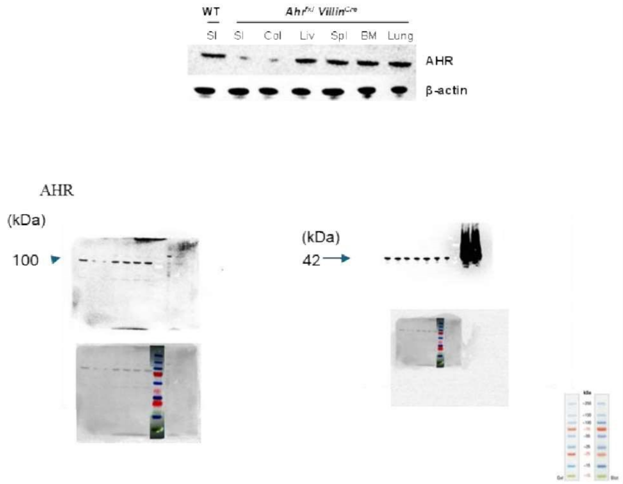

Supplement: Supplementary file 2 — Transparent Peer Review file [file 41467_2026_73760_MOESM2_ESM.pdf]
